# The Research of G–Motif Construction and Chirality in Deoxyguanosine Monophosphate Nucleotide Complexes

**DOI:** 10.3389/fchem.2021.709777

**Published:** 2021-06-30

**Authors:** Yanhong Zhu, Zhongkui Li, Pengfei Wang, Qi–Ming Qiu, Hongwei Ma, Hui Li

**Affiliations:** ^1^ Key Laboratory of Cluster Science of Ministry of Education, School of Chemistry and Chemical Engineering, Beijing Institute of Technology, Beijing, China; ^2^ Analytical and Testing Centre, Beijing Institute of Technology, Beijing, China

**Keywords:** base-pair mismatch, g–motif, self-assembly, chirality, crystal structure

## Abstract

A detailed understanding of the mismatched base-pairing interactions in DNA will help reveal genetic diseases and provide a theoretical basis for the development of targeted drugs. Here, we utilized mononucleotide fragment to simulate mismatch DNA interactions in a local hydrophobic microenvironment. The bipyridyl-type bridging ligands were employed as a mild stabilizer to stabilize the GG mismatch containing complexes, allowing mismatch to be visualized based on X-ray crystallography. Five single crystals of 2′-deoxyguanosine–5′–monophosphate (dGMP) metal complexes were designed and obtained *via* the process of self-assembly. Crystallographic studies clearly reveal the details of the supramolecular interaction between mononucleotides and guest intercalators. A novel guanine–guanine base mismatch pattern with unusual (*high anti*)–(*high anti*) type of arrangement around the glycosidic angle conformations was successfully constructed. The solution state ^1^H–NMR, ESI–MS spectrum studies, and UV titration experiments emphasize the robustness of this g–motif in solution. Additionally, we combined the methods of single-crystal and solution-, solid-state CD spectrum together to discuss the chirality of the complexes. The complexes containing the g–motif structure, which reduces the energy of the system, following the solid-state CD signals, generally move in the long-wave direction. These results provided a new mismatched base pairing, that is g–motif. The interaction mode and full characterizations of g–motif will contribute to the study of the mismatched DNA interaction.

## Introduction

The non–B-DNA secondary structures (Afek et al., 2020; Xiong et al., 2021), which are folded in a different manner from B-DNA or form unnatural base pairs that are not used for Watson–Crick (G≡C and A = T) base pairing (Watson and Crick, 1953; Brovarets’ et al., 2018), can induce genetic instability and cause a variety of human diseases (Brovarets’ et al., 2019; Li et al., 2020). However, the research of mismatched base-pairing interactions has great significance because they play an important role in various processes related to the biological function of nucleic acids (Iyer et al., 2006; Granzhan et al., 2014; Mondal et al., 2016), helping to reveal genetic diseases caused by the non–B-DNA structures. In biological systems, for example, aberrant amplification of the hexanucleotide GGGGCC (G4C2) repeated in the human *C9ORF72* gene is the most common genetic factor found behind frontotemporal dementia (FTD) and amyotrophic lateral sclerosis (ALS) (DeJesus-Hernandez et al., 2011). Recently, Tuân Phan group have shown the structures of DNA and RNA duplexes formed by G4C2 repeats, which alternately contain two types of GG mismatched base pair (Maity et al., 2021). Based on the structural analysis of mismatched base pairs in a variety of sequence contexts, it is shown that mismatches are highly polymorphic in nature; many of the mismatched base pairs can exist as protonated bases, such as bifurcated hydrogen bonds, wobble pairs, and various pairing conformations involving *syn*–*syn*, *anti*–*ant*, and *anti*–*syn* isomerization (Faibis et al., 1996; Varani and McClain, 2000; Ghosh et al., 2014).

How to construct and describe mismatched base-pairing interactions in structural details is a key issue for understanding the mechanism of the formation of non–B-DNA and finding effective ways to treat some genetic diseases. Numerous studies have shown that many small-molecule intercalators with pharmaceutical and/or diagnostic potential (Satange et al., 2018; Pages et al., 2019) can recognize mismatched DNA or RNA duplexes and induce various degrees of structural deformations. The existence of unstable mismatches in nucleic acid sequences may cause nucleobases flipping into additional helical positions, which itself is a significant phenomenon observed during the binding of small-molecule ligands to DNA or RNA duplexes (Jourdan et al., 2012; Tseng et al., 2017). DNA bending is also considered to be an important consequence of the action of those small molecules inserted into the DNA duplexes (Hou et al., 2002; Hall et al., 2011; Hall et al., 2014). Significantly, many structural features of small-molecule–DNA complexes have also been found in DNA–protein complexes, which indicate that in some cases, they may share similar interaction mechanisms (Chen et al., 2018; Da and Yu, 2018).

The non–B-DNA secondary structures known as G-quadruplex (G4), formed by alternative GG Hoogsteen mismatches at physiological temperature and potassium concentration, found in oncogene promoters and telomeres are often used as antitumor and antibacterial targets (Musumeci et al., 2016; Hansel-Hertsch et al., 2017; Hansel-Hertsch et al., 2018; Punt et al., 2020). The appealing possibility to treat cancers without impairing normal cells stimulated the synthesis of large libraries of putative selective G-quadruplex targeted ligands. For example, naphthalene diimides (NDIs) have a remarkable potential as anticancer drugs because of their well-proven ability to strongly interact with G-quadruplex (Salvati et al., 2016; Pirota et al., 2019; Platella et al., 2020a; Platella et al., 2020b). Encouraged by these results, many scientists use various optical methods (Gómez-González et al., 2018; Zhang and Wang, 2019; Lim and Hohng, 2020; Chen et al., 2021) and DFT theoretical calculations (Wu et al., 2012; Yao et al., 2013; Shi et al., 2015; Wang et al., 2019) to undertake an in-depth research on the interaction of intercalators and G-quadruplex, to reveal the structural details of this strong and specific binding. As we know, the visualization of these “host–guest” interactions based on X-ray crystallography will help understand the details of interactions between functional intercalations and targets, as well as the nucleotide conformational polymorphism changes. Although some related researches have been reported by single-crystal XRD (Monestier et al., 2017; Chu et al., 2019; Geng et al., 2019; Satange et al., 2019; Mao et al., 2020), the well-defined structure of “host–guest” interaction about intercalator-mismatched DNA remains as a fundamental and challenging issue for comprehensive understanding of the biological processes in genomic DNA. A proper design and synthesis of molecular building blocks is an effective methodological strategy to obtain innovative functional materials, which is an ultimate target in supramolecular chemistry. Based on the research of nucleotide–metal complexes, the diversities of coordination and supramolecular self-assembly inspired us to design and investigate the materials with programmable functions, by taking advantage of their unique properties including specific recognition ability, tunable conformations, and biocompatibility.

**SCHEME 1:**
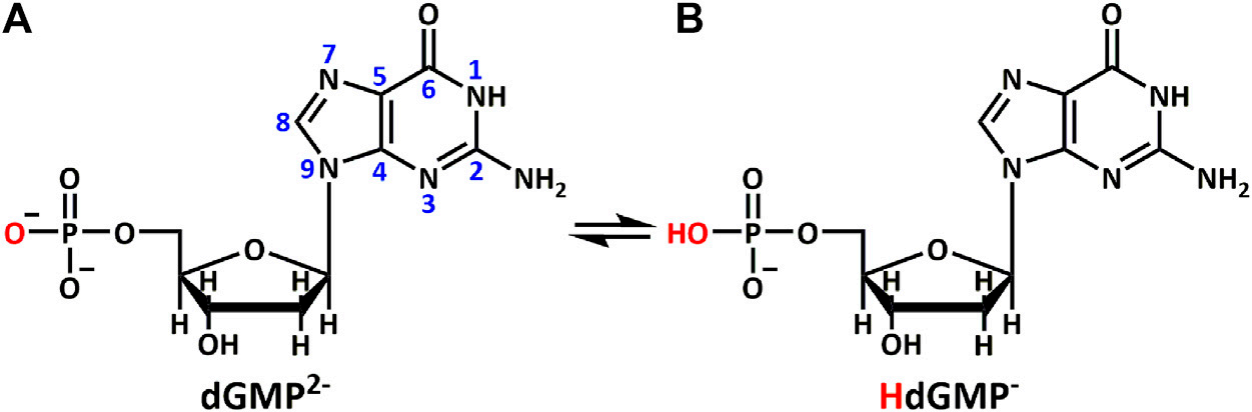
Different protonation states of **(A)** dGMP^2–^ and **(B)** HdGMP^–^ in aqueous solution.

In the current study, 2′–deoxyguanosine–5′–monophosphate (dGMP) fragment was used to simulate the interaction between small-molecule intercalators and mismatched DNA in a local hydrophobic microenvironment, in order to understand its structural consequences for DNA duplexes. We use bipyridine bridged ligands as a mild stabilizer in order to stabilize and enable crystallization of the GG mismatch containing complexes. Five complexes of dGMP with transition metals (Scheme 1), [Co(HdGMP)_2_(H_2_O)_4_]·(4,4′-bipy)·3H_2_O (1), [Co(HdGMP)_2_(H_2_O)_4_]·(bpe)·4H_2_O (2), [Co(HdGMP)_2_ (H_2_O)_4_]·(bpa)·4H_2_O (3), and {[Zn (bpda) (H_2_O)_4_]·(HdGMP)·4H_2_O}_n_ (4), [Mn (dGMP) (H_2_O)_5_]·3H_2_O (5), [4,4′–bipy = 4,4′–bipyridine, bpe = 1,2-*bis*(4-pyridyl)ethene, bpa = 1,2–*bis*(4-pyridyl)ethane, and bpda = 1,4-*bis*(4-pyridy)–2,3-diaza-1,3-butadiene] were successfully designed and obtained *via* the process of self-assembly. We use X-ray crystallography to observe the supramolecular interactions between small guest molecules and isolated mismatched base pair. A novel guanine–guanine base mismatch pattern with unusual (*high anti*)—(*high anti*) type of arrangement around the glycosidic angle conformations was successfully constructed. This base pair is different from GG Hoogsteen base pairs and reverse Watson–Crick GG mismatched base pairing (Mondal et al., 2016)^7^, but is similar to hemiprotonated CC+ and AA base pairs in i–motif and A–motif, respectively. So, we named it g–motif in this work. Precise structures indicate that the shorter auxiliary ligands produce the g–motif structure in which guanine is involved in the coordination at N7 site due to their smaller space; the longer bridging ligand provides a bigger hydrophobic microenvironment for guanine bases; thus, a perfect g–motif structure is presented. Certainly, the g–motif is not produced in the absence of auxiliary ligand. These results will be meaningful for revealing the genetic instability, and provide insights on intercalator–mismatch DNA interactions and a rationale. Obviously, it also provides a certain theoretical basis for the development of targeted therapeutic drugs.

## Results and Discussion

### Design and Synthesis

In biological systems, short nucleotide fragments (such as single nucleotides and dinucleotides) usually cannot form stable hydrogen-bonded base pairs or duplexes in aqueous environments until higher order oligomers are used (Philp and Stoddart, 1996), because water molecules are constant competitors. Hence, a majority of studies involve hydrogen bonds, which typically exploit additional weak interactions [aromatic stacking (Kato et al., 1995; Westover et al., 2004; Korostelev et al., 2006) or the hydrophobic effect (Jourdan et al., 1999; Hirschberg et al., 2000)] or employ noncompetitive organic solvents to shield from the competing water molecules.

In our strategy, the bipyridyl-type bridging ligands with different sizes were chosen as a multifunctional auxiliary ligand; on the one hand, they can precisely adjust the orientation of purine bases through stacking interactions; on the other hand, they can provide an ideal flat, hydrophobic microenvironment with different interplanar distances for the binding of single planar aromatic molecules (nucleobase or nucleotide). Additionally, they can prevent the nonenzymatic hydrolysis of nucleotide phosphate groups and increase the crystallization of nucleotide complexes, because the bridging ligands would coordinate to metal ions by competing with dGMP as a structure modifier. As a result, under the co-control of auxiliary ligands and solvent based on the above design idea, we successfully synthesized and obtained 1–4 in water–ethanol solution (the ratio is 2:1) and 5 in water solvent.

### Crystal Structure and G–motif

All of the complexes were obtained at a slightly acidic condition and studied by the X-ray single crystal diffraction method. Complexes 1–3 and 5 are mononuclear nucleotide complexes, but 4 is a 1D coordination polymer. The nucleotides in 1–3 are coordinated with metal ions, and the auxiliary ligands exist as guest molecules. However, 4 is opposite to 1–3, which is a 1D coordination polymer linked by the auxiliary ligand as a bridge ligand and the nucleotide is uncoordinated. Complex 5 is a binary complex without the participation of auxiliary ligands. The protonated nucleotide HdGMP can be determined by the length of the longer uncoordinated P–O bond *via* X-ray diffraction at a good accuracy. The crystallographic data for complexes 1‒5 are summarized in Table 1. Interestingly, ternary nucleotide complexes (1–4) with the auxiliary ligand can be obtained only in organic solvents, while binary nucleotide complex (5) can be obtained just in pure water environment. Clearly, the solvent effect also plays an important and effective role to control the formation of nucleotide complexes.

**TABLE 1:** Crystallographic data for complexes 1‒5. Bold values are CCDC number of Crystals.

Complex	1	2	3	4	5
Formula	C_30_H_50_CoN_12_O_22_P_2_	C_32_H_52_CoN_12_O_22_P_2_	C_32_H_54_CoN_12_O_22_P_2_	C_32_H_52_N_14_O_22_P_2_Zn	C_10_H_28_MnN_5_O_15_P
*Mr*	1,051.69	1,077.72	1,079.74	1,112.18	544.28
Crystal system	Orthorhombic	Monoclinic	Monoclinic	Orthorhombic	Monoclinic
Space group	*P*2_1_2_1_2	*P*2_1_	*P*2_1_	*P*2_1_2_1_2	*C*2
*a* (Å)	15.2094(9)	6.9901(7)	7.0917(2)	15.7257 (5)	27.735 (2)
*b* (Å)	20.6549(12)	20.7406(19)	20.6519(7)	21.0314 (7)	11.2485 (11)
*c* (Å)	7.0696(4)	15.2603(14)	15.2533(5)	6.9203 (2)	6.7722 (6)
*α* (°)	90	90	90	90	90
β (°)	90	92.454(3)	92.7700(10)	90	92.021 (8)
γ (°)	90	90	90	90	90
*V* (Å^3^)	2,220.9(2)	2,210.4(4)	2,231.34(12)	2,288.78 (12)	2,111.4 (3)
*Z*	2	2	2	2	4
F (000)	1,094.0	1,122.0	1,126.0	1,156.0	1,132.0
Reflections collected	24,804	30,266	21,882	22,389	8,544
Independent reflections	4,711	12,673	7,887	4,037	3,994
Goodness–of–fit on *F* ^2^	1.093	0.976	1.024	1.052	1.027
Completeness to 2θ	53.46,100.0%	60.08,99.9%	50.04,99.9%	50.03, 99.9%	53.46, 99.5%
*R*_int_	0.0341	0.0564	0.0263	0.0270	0.0461
*R*_1_ [*I* > 2σ(*І*)]	0.0760	0.0516	0.0319	0.0813	0.0379
*wR*_2_ [*I* > 2σ(*І*)]	0.2264	0.0865	0.0799	0.2224	0.0806
*R*_1_ (all data)	0.0855	0.1052	0.0371	0.0903	0.0450
*wR*_2_ (all data)	0.2372	0.1029	0.0826	0.2319	0.0844
Flack parameter	0.02(5)	0.012(18)	0.011(15)	0.015 (7)	–0.02 (2)
CCDC Number	**2,064,323**	**2,064,324**	**2,064,325**	**2,064,326**	**2,064,327**

Complexes 1–3 are essentially isomorphous, except for the size of the bridging ligand (Supplementary Figure S3). Since the size of the auxiliary ligand in 2 (bpe = 9.4 Å) is greater than that of 1 (bipy = 7.1 Å) and the stiffness is stronger than that of 3, we take 2 as an example for structural analysis. In addition, the molecular structure diagrams and related hydrogen bond information of 1 and 3 are given for comparison (Supplementary Figures S4–S8). The single crystal X-ray diffraction analysis reveals that Co(II) central in 2 is six-coordinated, showing a N_2_O_4_ octahedral coordination mode with two imidazole N atoms (N2 and N7) from HdGMP at the axial positions and four oxygen atoms from water molecules at the equatorial positions (Supplementary Figure S1). Another four water molecules and one bpe molecule exist as guest molecules and play an important role in the geometry and conformation of the nucleoside moiety. The terminal P–O bond lengths are 1.565(4) Å for *p* (1)–O (4) and 1.561(4) Å for *p* (2)–O (10) in 2, whereas the mean value of P–O is 1.517(3) Å for nonprotonated nucleotides (Supplementary Tables S3, S6), so the longer bond length indicates that only those oxygen atoms (O4 and O10) are protonated.

**FIGURE 1:**
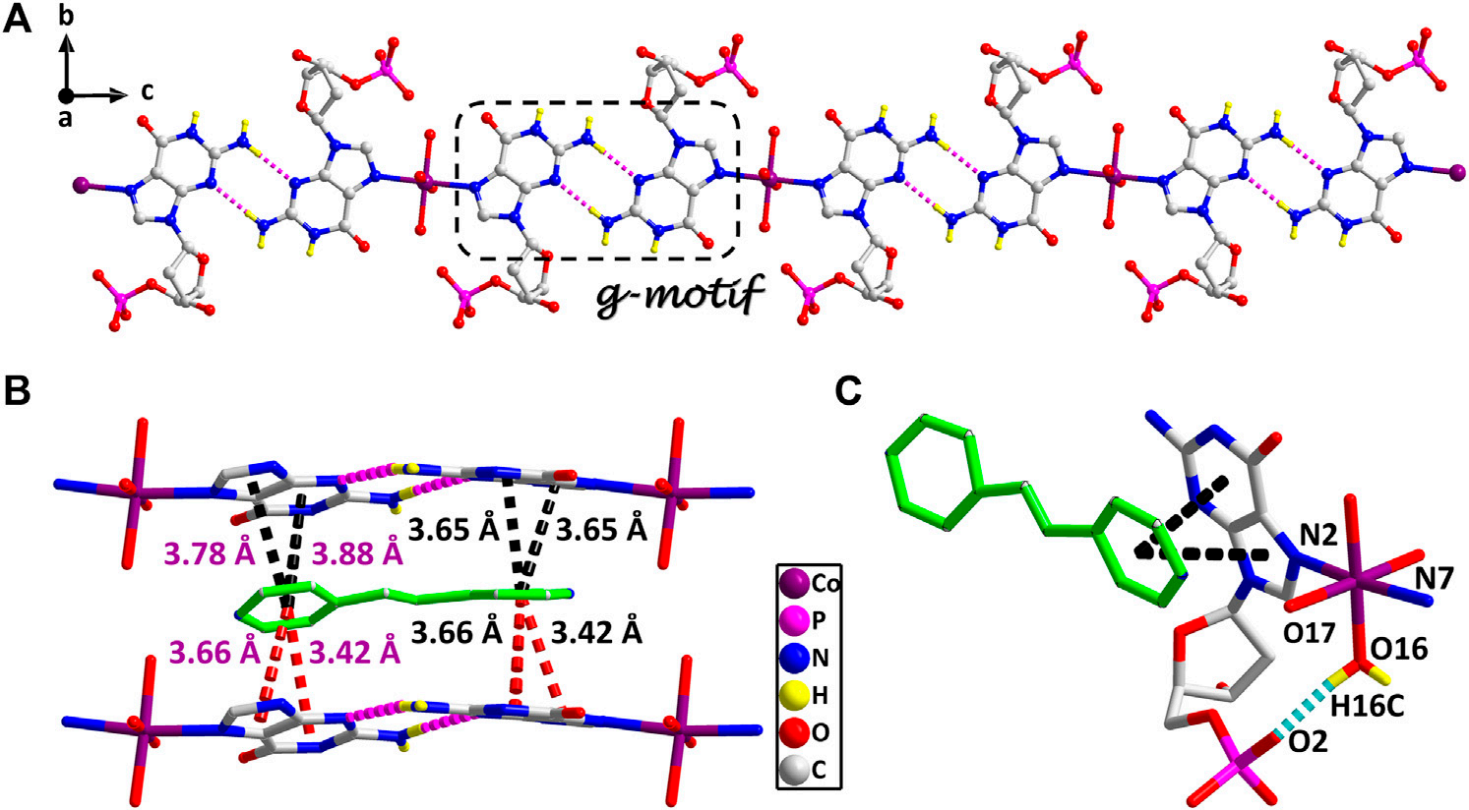
**(A)** The 1D supramolecular structure of two viewed from *a* axis. **(B)** The π–π stacking interactions in the 2D structure of two. **(A)** Various noncovalent interactions in high *trans* conformation (O16–H16C···O2, 0.84 Å, 1.90 Å, 2.70 Å, 159°). Partially shaded area of **(A)** is the g–motif structure (N5–H5A···N9, 0.86 Å, 2.11 Å, 2.96 Å, 171°; N10–H10 A···N4, 0.86 Å, 2.13 Å, 2.99 Å, 175°) (cobalt: violet, carbon: gray, hydrogen: yellow, oxygen: red, nitrogen: blue, and phosphorus: pink).

The stereochemistry around the glycosidic bond C (1′)–N bond angle χ for the purine bases in two is unusual. They are measured by the C (8)–N (1)–C (5)–O (5) and C (18)–N (6)–C (15)–O (12) (χ) torsion angle, which is –76 (1)° for the N (1)–bound Co(II) central and –82 (1)°for the N (6)–bound Co(II) central, respectively. This special conformation can be described as high *anti*, –*sc* (Cini and Pifferi, 1999; Asami et al., 2012) for the coordinated nucleotide (Supplementary Figure S2). To the best of our knowledge, this extreme position described as high *anti* is not frequent for nucleoside and has never been found before for the solid-state structures of dGMP nucleotides. Intriguingly, upon studying the H-bonding of 2, we found that the complex has a novel guanine–guanine base mismatch pattern (Figure 1A). Because of very limited examples in the GG mismatch base pairs with the structural details and in order to specify the mismatch base pair study as well, the g–motif has been named for the first time in this manuscript. Compared with the hydrogen bonds of G–quadruplex, the g–motif base pair of two has a slightly longer bond length and a larger angle except for the absence of N–H···O-type hydrogen bonds (Complex 2: N–H···N, 2.96–2.99 Å, 171°–175°; G–quadruplex: N–H···N, 2.88–2.91 Å, 166–170°, N–H···O, 2.86–2.92 Å, 159–165°). Simultaneously, this kind of H-bonding can expand the structure of this complex from 0D into a 1D linear chain (Figure 1A).

These observations lead us to suspect that bridging ligands play a key role in relating the stereochemistry of the base with respect to the sugar and the formation of the g–motif structure. Further studies indicated that the deviations of the Co(II) central from the plane of the purine bases are obviously smaller (0.18 Å and 0.18 Å) in 2, and the N(2)–Co–N(7) angle is close to 180° (172°). However, compared with other complexes (Poojary and Manohar, 1988), the coordination geometry and mode of metal central are the same as that of 2, except for the guest molecule, which forms a sharp contrast with 2. In those complexes, the Co(II) central is significantly out of the plane of the purine rings, and the deviations were 0.67–0.68 Å. Even though the N–Co–N angles are about 82.3–84.5°, the dihedral angle between the two purine bases is only 40–50°. Therefore, the noncovalent interactions between purine bases and auxiliary ligands in 2 can fix and stabilize this extreme conformation of purine bases, and further induce it to form an almost perfect plane along *a* axis (Figure 1A). This hypothesis was confirmed by a crystal structure analysis, in which multiple π–π stacking interaction was formed between the auxiliary ligands and the nucleobases (Figure 1B; Supplementary Figure S4). Another reason is the strong intramolecular hydrogen bonds among the coordinated water molecules (O15 and O16) with the phosphate oxygen atoms (O2 and O9) and carbonyl oxygen atoms of the purine base (O7 and O14), which can also limit the flexible conformation of guanine nucleotides (Figure 1C). It is worth noting that this extreme conformation high *anti* is caused by the cooperation of those above noncovalent interactions.

Furthermore, the 1D linear chains are combined into a 2D supramolecular sheet *via* N–H···O hydrogen bonds between pyrimidine nitrogen atoms (N3 and N8) and the phosphate oxygen atoms (O3 and O11) in the adjacent chains (Supplementary Figure S6). Based on hydrogen bonds formed by the hydroxy group of the pentose ring with carbonyl oxygen atom of the base, these 2D structures can be further assembled into a 3D sandwiched framework (Supplementary Figure S7). Although the bridging ligand bpe does not participate in the coordination, it plays a decisive role in the formation of the g–motif structure. As a Lewis base, bpe can not only act as a buffer regulator of solution pH but also induce the formation of g–motif through stacking interaction. The shorter bridging ligand 4,4′-bipy and the more flexible auxiliary ligand bpa can also still induce the formation of the g–motif structure. The structural details of their g–motif can be seen in Supplementary Figure S5. Due to the narrow hydrophobic microenvironment of 4,4′-bipy in 1, the π–π stacking interaction between the pyridine ring and the purine base is significantly weakened. So the corresponding hydrogen bond interaction in 1 is slightly weaker than that in 2. The length of bpa is close to bpe, so the hydrogen bond strength of g–motif in 2 and 3 is almost the same. However, the dihedral angle of g–motif is less than that of 2 for the flexibility of bpa, which makes it more adaptable to the orientation of purine bases.

**FIGURE 2:**
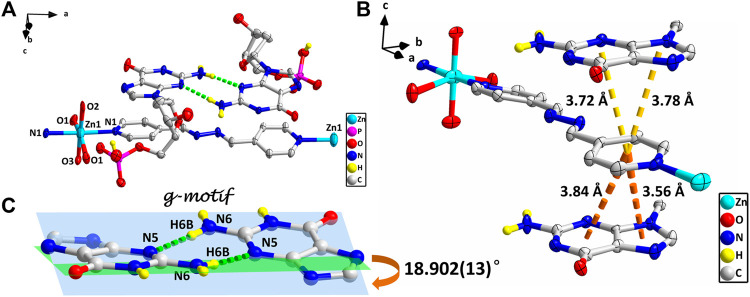
**(A)** Molecular structure of four. The uncoordinated water molecules and part of hydrogen atoms are omitted for clarity. **(B)** The π–π stacking interactions in four. **(C)** g–motif and dihedral angle in four (N6–H6A···N5, 0.86 Å, 2.18 Å, 3.04 Å, 177°). The protonation of phosphate is highlighted by yellow hydrogen atoms. (zinc: turquoise, carbon: gray, hydrogen: yellow, oxygen: red, nitrogen: blue, and phosphorus: pink).

However, studies have shown that due to blockage of N7 of the purine ring, the same hydrogen bonding patterns occur on the minor groove binding face in this nucleobase (Sigel et al., 2002). To further demonstrate the universality of this strategy, we chose bpda (11.2 Å) with larger molecular length as the bridging ligand, and fortunately obtained the single crystal structure of 4. X-ray diffraction shows that 4 is made up of 1D linear [Zn (bpda) (H_2_O)_4_]^+^ cationic chains, uncoordinated partially protonated [HdGMP]^−^ anions, and guest water molecules. The protonated nucleotide [HdGMP]^−^ can still be determined by the relatively longer uncoordinated P‒O bond length (Supplementary Table S5; Supplementary Figure S9). The coordination geometry of the Zn(II) central in the 1D Zn (bpda) (H_2_O)_4_^2+^ chain is presently a deformed octahedral geometry with two nitrogen atoms from two different bridging molecules at the axial positions and four coordinated water molecules in the equatorial sites (Supplementary Figure S9). It is remarkable that the guest partial protonation [HdGMP]^–^ anions also have g–motifs, and the dihedral angle of the base rings is 18.9 (2)° (Figures 2A,C). Compared with 1–3, the N7 site in 4 does not coordinate with the metal ion, but still produced the same base-pair mismatch, which well demonstrated the effectiveness of our strategy. There are multiple π–π stacking interactions between the coordinated bridging ligand bpda and the guest [HdGMP]^−^ anions (Figure 2B). The Zn···Zn distance of four bridged by the bpda is 15.702 (1) Å. Each pyridine ring of bpda is basically directly opposite to the purine base part of [HdGMP]^−^ (Figure 2B), and the distance of the –C=N–N=C– part of bpda exactly matches the space required to form a guanine–guanine base pair, so that the prospective g–motif structure has been obtained. If the bpda ligands are ignored, these dimers, which were connected by g–motif, obtain a 2D H-bonding layer through hydrogen bonds between the base and the phosphate oxygen (Supplementary Figure S10). Then, the same layers are further linked into the 3D supramolecular structure *via* the hydrogen bonds between the sugar ring hydroxyl and the carbonyl group on the base (Supplementary Figure S11A). While there are electrostatic interaction and hydrogen bonds between the 1D [Zn (bpda) (H_2_O)_4_]^+^ cationic chain and the nucleotide anion in this 3D structure (Supplementary Figure S11B). It is worth noting that the value of the glycosyl torsion angles χ C (4)–N (9)–C (1′)–O (1′) is –83.4°, which was also corresponding to the high *anti* conformation. This discovery proves the importance of the conformation of nucleoside moiety once again.

**FIGURE 3:**
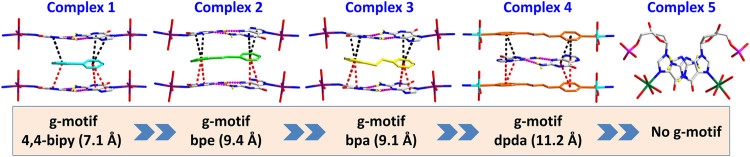
Summary of crystal structures presenting g–motif and π–π stacking interaction in complexes 1–5.

Complex 5 was obtained in pure water, and the Mn(II) center is also present as a slightly distorted octahedral geometry with five coordinated water molecules and one dGMP^2–^ ligand by oxygen atoms and imidazole N atoms, respectively (Figure 3; Supplementary Figure S12). The mean value of bond length Mn–O_*water*_ is 2.201 (2) Å, which is in accordance with that in the previous reports (Lin et al., 2018). In this complex, the symmetry-related nucleotides coordinate in the *cis* position. Although the central metal Mn(II) is significantly in the plane of the purine rings, no base pairing was found in its crystal structure. Why? Compared with 1–4, this may be attributed to the lack of participation and induction of auxiliary ligands, thus lacking the local environment of the hydrophobic required for base pairing. Additionally, the value of the glycosyl torsion angles χ is –163° corresponding to *anti* conformation. This counterexample also confirms the key role of the high *trans* conformation in the formation of g–motif.

### Studies of G–Motif in Solution

**FIGURE 4:**
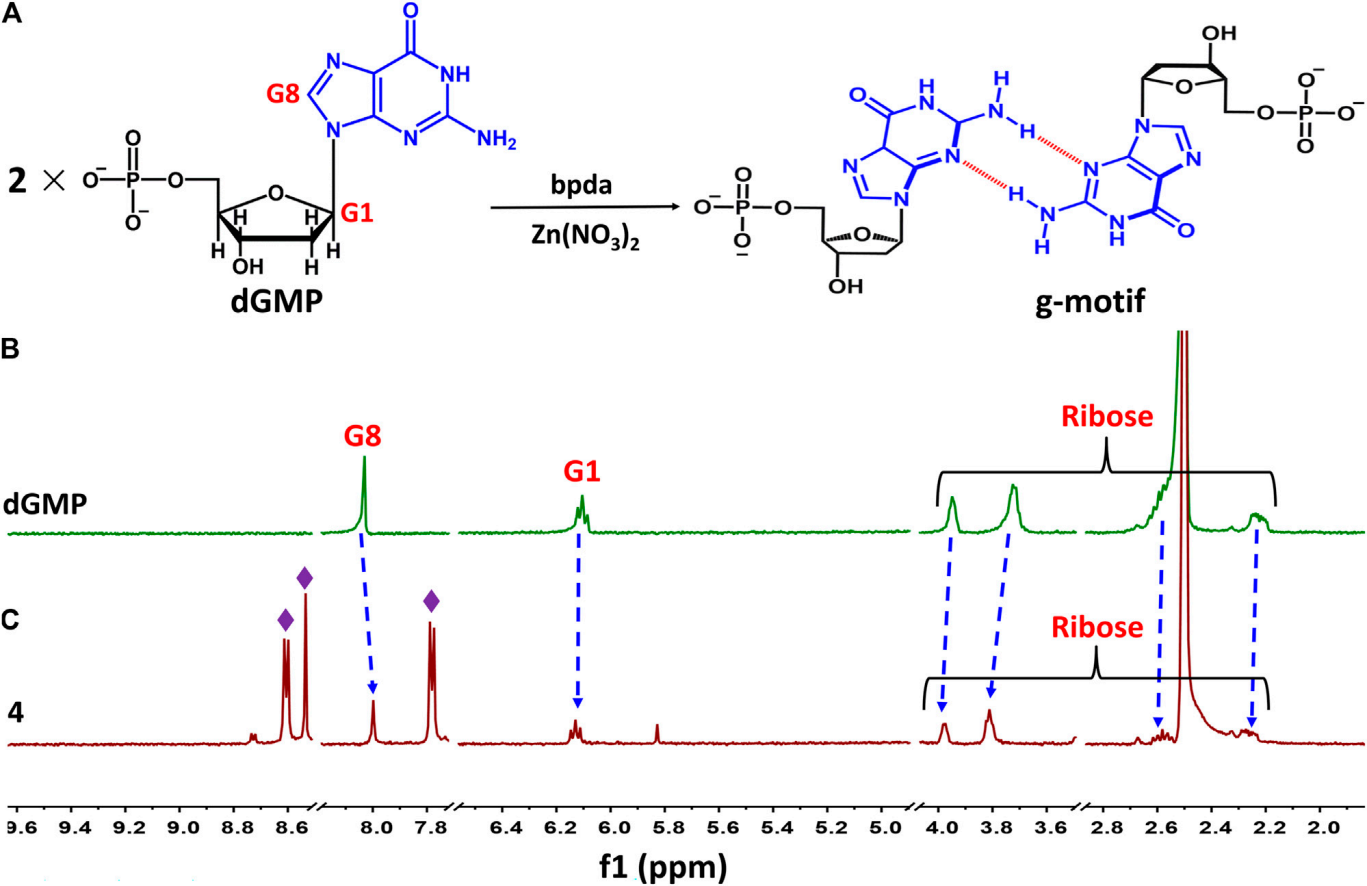
**(A)** Schematic representation of g–motif formation. **(B)** ^1^H–NMR spectrum of dGMP ligand in solution (D_2_O: DMSO–*d*_*6*_ = 2:1) at room temperature. **(C)** ^1^H–NMR spectrum of 4 in solution (D_2_O: DMSO–*d*_*6*_ = 2:1) at room temperature.

Taking 4 as an example, we illustrate the formation of the g–motif structure in a solution. Stirring an aqueous solution of 2′-deoxyguanosine-5′-monophosphate (dGMP, 0.05 mmol) and Zn(NO_3_)_2_ (0.05 mmol) in the presence of bpda bridging ligand (0.05 mmol) resulted in the formation of four, as evidenced by ^1^H–NMR spectroscopy (Figure 4A). In order to increase the solubility of the complex, we mixed a small amount of DMSO organic solvent with a ratio of 2:1.

The nucleobase protons were shifted upfield and downfield (Δδ = 8.03→8.00 ppm and 6.11→6.13 ppm) for guanosine H8 and H1 of dGMP (Figures 4B,C; Supplementary Figure S23), respectively. The ribose protons signal around 2.24–3.94 ppm shift slightly, indicating the existence of little changes in the environment. The ratio of peak area of four indicates that the molecular number ratio of dGMP to bpda is 2:1 (Supplementary Figure S24). These observations may suggest that the nucleobase moieties are located on the inner side of the bpda *via* stacking interaction, and that the ribose part also undergoes conformational changes. The three aromatic signals derived from bpda (8.75, 8.70, and 7.83 ppm, Supplementary Figure S23) are much sharper, presumably owing to fast formation processes of the g–motif structure in 4 (Sawada et al., 2009; Sawada and Fujita, 2010). Additionally, we successfully captured g–motif structural fragments in ESI-MS spectrometry (Supplementary Figure S25), which further proved that g–motif can form in a solution.

In order to study the interactions of bridging ligands (4,4′–bipy, bpe, bpa, bpda) with M–dGMP (M = Co^2+^, Zn^2+^) in an aqueous medium, UV titrations were carried out by adding an auxiliary ligand to the solution of M-dGMP. UV absorbance was measured as a function of concentration of bridging ligands. The measurement results are shown in Supplementary Figure S21. Isosbestic points were not found in all titration curves, indicating that the auxiliary ligand does not directly interact with the M-dGMP system. The high-quality crystal data of the complexes show that only the host–guest interaction existed between the auxiliary ligand and dGMP. With increased auxiliary ligand concentration, the apparent intensity increased at 250–300 nm and broadened the main absorption at 265 nm. Those changes come from the strong π–π* transition induced by the intermolecular interaction between auxiliary ligands and dGMP based on UV-*vis* absorption spectra of individual dGMP, bridging ligands and complexes (5 × 10^−5^ mol/L, Supplementary Figure S20), rather than a simple superposition of the auxiliary ligand and the absorption spectrum of M-dGMP, and the intermolecular interaction can also be proved by the single crystal structure of the complexes.

### Circular Dichroism Spectroscopy

The Flack absolute parameters clearly indicate that all these coordination complexes exhibit homochirality. There are two kinds of chirality sources: 1) the intrinsic chirality of the pentose ring of nucleotides and 2) supramolecular helical chirality formed by noncovalent bonds (H-bonding and π–π stacking). All the chiral sources of these complexes were investigated in detail based on their crystal structures and circular dichroic spectrums.

**FIGURE 5:**
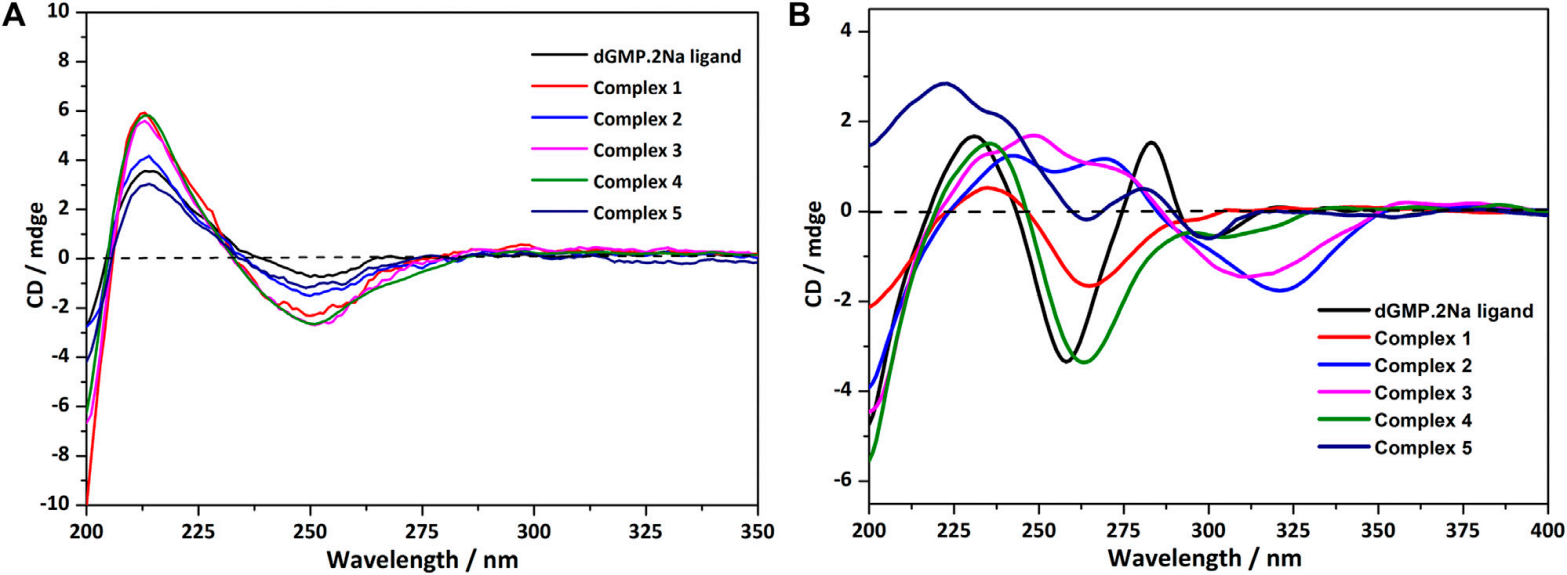
**(A)** The CD spectrum of the solution of dGMP and complexes 1–5; the spectrum was obtained by measuring 2.5 × 10^−5^ mol/L solution in a 1-nm cell. **(B)** The crystallized solid-state CD spectrum of dGMP and complexes 1–5 (KBr: (sample) = 200: 1).

In an aqueous solution, the negative CD signal near 250 nm and the positive Cotton effect at 220 nm for dGMP ligand (Figure 5A) correspond to the pentose ring and base n–σ*, n–π* transition absorption, and π–π* transition (Brunner and Maestre, 1975; Holm et al., 2007) between adjacent bases, respectively, which are consistent with its UV absorption spectrum (Supplementary Figure S19). Compared with the dGMP ligand, the UV-*vis* spectra of 5 is basically consistent with the ligand, but the UV-*vis* spectra intensity of 1–4 is significantly increased and obviously broadening, which may be attributed to the formation of conjugate structures between the bridging ligand and the nucleotide. In the CD spectrum, the signal peaks of the complexes and the ligand are almost the same, indicating that the chirality of the complexes depends on the inherent chirality of the ligand (Figure 5A). The negative Cotton effect of 1–5 is slightly enhanced at 250 nm, which is due to the mutarotation of the dGMP molecule in aqueous solution, resulting in α–- and β-type enantiomers, and the coordination of metal ions enhances the advantage of the β-type enantiomer (Ingwall, 1972).

The solid CD spectra of the dGMP ligand and complexes are shown in Figure 5B, and the relevant summary is shown in Supplementary Figure S15. Among them, 1 and 4, and 2 and 3 are similar, which may depend on the same space group (Supplementary Table S1). Complex 5 almost maintains the chiral signals of the ligand. Based on our research on the solid-state CD spectrums and crystal structures of the dGMP and GMP ligands, the chiral signals at 281 nm (–) and 301 nm (+) may be attributed to the pentose ring envelope **(E)** conformation (Supplementary Figures S14–18; Supplementary Tables S13, S14). The Cotton effects centered at 260 nm (–) have a slight redshift, and the signal is almost reversed, which is caused by the widespread π–π stacking interactions and supramolecular helical structure along *b* axis, respectively. Complexes 1 and 4 generally have a slight redshift at the absorption peak at 235 nm (+) relative to the dGMP ligand, which can be explained by the formation of the g–motif. Crystal analysis shows that the formation of N–H···N hydrogen bonds between the purine bases reduces the energy of the system, so n–π* transition does not need to absorb higher energy. On the contrary, due to the lack of the g–motif structure, this absorption peak blue shifted to 222 nm (+) in 5. The negative Cotton effect about 263/264 nm (+) was also redshifted relative to the ligand in 1 and 4, which was related to the extensive and very strong π‒π stacking interaction. Curiously, the chiral signals of 2 and 3 at Cotton effect 258 nm (–) changed, and new peaks are generated at 312–318 nm. This may be attributed to the supramolecular helical structure constructed by hydrogen bonds, which, as a new source of chirality, changes the symmetry of electronic transitions, causing the chiral signal to reverse (Maeda et al., 2006). The new peaks may be attributed to the supramolecular helix chirality of the complexes. Further crystallographic research shows that the 3D supramolecular structure of 2 and 3 contains a quadruple helical structure constructed by hydrogen bonds (O6–H6···O7, 1.91 Å, 2.71 Å, 169°; O13–H13···O14, 2.01 Å, 2.81 Å, 168°, and N3–H3···O3, 1.84 Å, 2.70 Å, 173°; N8–H8···O11, 1.87 Å, 2.73 Å, 176°). The chiral conformation of the quadruple helical in 2 and 3 exhibits *P*-chirality because the dGMP ligand wraps around the *b* axis in a clockwise manner (Supplementary Figure S8). Compared with 3, complex 2 has stronger H-bonding, so a slight redshift occurs (312→318 nm).

## Conclusion

In summary, a rational design and construction of the GG mismatch base pairing has been achieved by dGMP with pyridine derivatives to simulate a local hydrophobic microenvironment in DNA, which was named as the g–motif for the first time, in order to specify the mismatch base pairs. There are fully characterized g–motif both in solution and the crystallized solid state. Especially, single crystal structural analysis for the g–motif in the five nucleotide coordination complexes provides the structural details of the GG mismatch base pairing. In this work, the interaction mode of the g–motif shows an unusual (*high anti*)–(*high anti*) pattern. The shorter auxiliary ligands with different sizes produce a suitable space for the formation of the g–motif, in which guanine is involved in the coordination of N7 donor. However, the g–motif does not appear in the coordination complex without auxiliary ligand. The co-assembly of these DNA intercalators and nucleotides produces supramolecular crystals arranged through a combination of π−π stacking and hydrogen-bond interactions, which overcomes the inherent limitations of self-assembly leading to materials with unprecedented properties. Our research critically expands the breadth of programmable and functional materials attainable by self-assembly. In solution, the g–motif also exists and is confirmed by ^1^H–NMR and ESI-MS spectrum. In addition, the strong π–π stacking interaction between the auxiliary ligand and dGMP can be detected by the UV–*vis* titration. The chirality of the coordination complexes has been studied by the method of solid-state CD spectra combining with X-ray crystal diffraction analysis which has been developed in our laboratory, which is an effective way to help us to understand the g–motif comprehensively, both the structure and the properties. Primarily, the g–motif can be identified in a crystallized state CD spectrum by the redshift coming from the hydrogen bond in g–motif. Additionally, some small-molecule ligands, such as rhodium and platinum metalloinsertors, have been reported to target mismatch DNA, with important applications in the therapy and diagnosis of cancer. However, many of these metalloinsertors are generally highly cytotoxic with many different side effects. The understanding of the structure interaction in current research that can be regarded as a chemical tool for interrogating and detecting mismatch-related diseases is expected to be helpful to guide the development of future generations of more selective targeted drugs.

## Experimental Section

### Materials and Instrumentation

All chemical reagents were commercially available and used without further purification. Co(NO_3_)_2_·6H_2_O, Zn(NO_3_)_2_·6H_2_O, Mn(NO3)2·4H2O, and 4,4′-bipyridine (bipy) were purchased from Adamas, 1,2-bis (4-pyridyl)ethane (bpe) and 1,2-bis (4-pyridyl)ethane (bpa) were purchased from Tci, and 2′-deoxyguanosine-5′-monophosphate disodium salt hydrate (dGMP) was purchased from Alfa Aesar.

Elemental analyses (C, H, and N) were determined on an EA3000 elemental analyzer. FT-IR spectra were recorded on a Nicolet Nexus FT-IR spectrometer using the KBr pellet in the range of 4,000–400 cm^−1^. UV-*vis* spectra were obtained from a TU-1950 spectrophotometer. X-ray powder diffraction studies were performed by a Bruker D8 Advance X-ray diffractometer. The X-ray single crystal data collections were performed on a Bruker APEX-II CCD and Rigaku Saturn724+ (2 × 2 bin mode) diffractometer with graphite monochromatized MoKα radiation (λ = 0.71073 Å). CD measurements were carried out under a constant flow of nitrogen on a JASCO J–810 spectropolarimeter. Thermogravimetric analyses (TGA) were carried out using a DTG-60H thermal analyzer under nitrogen atmosphere from room temperature to 800°C with a heating rate of 5 C/min. The pH of the sample solution was measured using a PHS-3C meter.

### Synthesis and Structural Characterization

The synthesis method of all complexes is that an aqueous solution (5 ml) of 2′-deoxyguanosine-5′-monophosphate disodium salt (dGMP) was added into an aqueous solution (5 ml) of M(NO_3_)_2_ (M = Mn^2+^, Co^2+^, and Zn^2+^). After the mixture was stirred for 10–15 min, a solution of bridging ligand (4,4-bipy, bpe, bpa, bpda) that dissolved in distilled water or ethanol (5 ml) was added. The suspension acidity was adjusted by HNO_3_ (1 M) until the solution became clear. The resulting solution was stirred at room temperature for 20–30 min and then filtered. Single crystals suitable for X-ray diffraction analysis can be obtained by slow evaporation under room temperature. It should be noted that except for 5, all other complexes were obtained in water–ethanol mixed solvent.

Powder X-ray diffraction (PXRD) patterns of polycrystalline samples of the ligands and complexes were all coincident with their theoretical ones (Supplementary Figure S25), confirming the phase purity of the bulk samples and their isostructurality with the crystals selected for single-crystal X-ray diffraction. The water contents of the complexes and thermal stability were estimated by thermogravimetric analysis (TGA) (Supplementary Figure S27). Elemental analysis (C, H, and N) further confirmed the chemical identity of the complexes determined by single-crystal X-ray diffraction. The types of metal–nucleotide interactions have been identified by the FT-IR (Supplementary Figure S26).

## Data Availability

The original contributions presented in the study are included in the article/Supplementary Material; further inquiries can be directed to the corresponding author.
