# Supplementary material for: The Research of G–Motif Construction and Chirality in Deoxyguanosine Monophosphate Nucleotide Complexes
Source: Front Chem. 2021 Jun 30;9:709777. doi: 10.3389/fchem.2021.709777 (PMC8278404; doi:10.3389/fchem.2021.709777)

# checkCIF/PLATON report

You have not supplied any structure factors. As a result the full set of tests cannot be run.

THIS REPORT IS FOR GUIDANCE ONLY. IF USED AS PART OF A REVIEW PROCEDURE FOR PUBLICATION, IT SHOULD NOT REPLACE THE EXPERTISE OF AN EXPERIENCED CRYSTALLOGRAPHIC REFEREE.

No syntax errors found. CIF dictionary Interpreting this report

## Datablock: 2_a

---

```
Bond precision:  C-C = 0.0103 A          Wavelength=0.71073

Cell:            a=15.2094(9)     b=20.6549(12)     c=7.0696(4)
                 alpha=90         beta=90           gamma=90
Temperature:     296 K
```

| | Calculated | Reported |
|---|---|---|
| Volume | 2220.9(2) | 2220.9(2) |
| Space group | P 21 21 2 | P 21 21 2 |
| Hall group | P 2 2ab | P 2 2ab |
| Moiety formula | C20 H34 Co N10 O18 P2, C10 H8 N2, 4(H2 O) | C20 H34 Co N10 O18 P2, 4(H2 O), C10 H8 N2 |
| Sum formula | C30 H50 Co N12 O22 P2 | C30 H50 Co N12 O22 P2 |
| Mr | 1051.69 | 1051.69 |
| Dx,g cm-3 | 1.573 | 1.573 |
| Z | 2 | 2 |
| Mu (mm-1) | 0.554 | 0.554 |
| F000 | 1094.0 | 1094.0 |
| F000’ | 1095.69 | |
| h,k,lmax | 19,26,8 | 19,26,8 |
| Nref | 4708[ 2698] | 4711 |
| Tmin,Tmax | 0.899,0.931 | 0.575,0.746 |
| Tmin’ | 0.866 | |

```
Correction method= # Reported T Limits: Tmin=0.575 Tmax=0.746
AbsCorr = MULTI-SCAN

Data completeness= 1.75/1.00      Theta(max)= 26.728

R(reflections)= 0.0760( 4122)     wR2(reflections)= 0.2372( 4711)

S = 1.093                  Npar= 336
```

---

The following ALERTS were generated. Each ALERT has the format
**test-name_ALERT_alert-type_alert-level**.
Click on the hyperlinks for more details of the test.

---

## Alert level C

```
PLAT094_ALERT_2_C Ratio of Maximum / Minimum Residual Density ....       2.29 Report
PLAT220_ALERT_2_C NonSolvent   Resd 1  O   Ueq(max)/Ueq(min) Range        3.6 Ratio
PLAT222_ALERT_3_C NonSolvent Resd 1  H   Uiso(max)/Uiso(min) Range        5.3 Ratio
PLAT234_ALERT_4_C Large Hirshfeld Difference Co1      --O10      .       0.20 Ang.
PLAT242_ALERT_2_C Low    'MainMol' Ueq as Compared to Neighbors of        Co1 Check
PLAT242_ALERT_2_C Low    'MainMol' Ueq as Compared to Neighbors of         P1 Check
PLAT260_ALERT_2_C Large Average Ueq of Residue Including       O14      0.172 Check
PLAT341_ALERT_3_C Low Bond Precision on  C-C Bonds ...............    0.01033 Ang.
PLAT417_ALERT_2_C Short Inter D-H..H-D       H10      ..H14B     .       2.13 Ang.
                                                 1-x,1-y,z  =      2_665 Check
PLAT420_ALERT_2_C D-H Without Acceptor       N5       --H5B      .     Please Check
PLAT480_ALERT_4_C Long H...A H-Bond Reported H5B      ..O3       .       2.64 Ang.
```

---

## Alert level G

```
PLAT002_ALERT_2_G Number of Distance or Angle Restraints on AtSite          2 Note
PLAT003_ALERT_2_G Number of Uiso or Uij Restrained non-H Atoms ...         15 Report
PLAT007_ALERT_5_G Number of Unrefined Donor-H Atoms ..............         13 Report
PLAT042_ALERT_1_G Calc. and Reported MoietyFormula Strings  Differ     Please Check
PLAT072_ALERT_2_G SHELXL First  Parameter in WGHT  Unusually Large       0.16 Report
PLAT172_ALERT_4_G The CIF-Embedded .res File Contains DFIX Records          1 Report
PLAT177_ALERT_4_G The CIF-Embedded .res File Contains DELU Records          1 Report
PLAT178_ALERT_4_G The CIF-Embedded .res File Contains SIMU Records          1 Report
PLAT186_ALERT_4_G The CIF-Embedded .res File Contains ISOR Records          4 Report
PLAT187_ALERT_4_G The CIF-Embedded .res File Contains RIGU Records          1 Report
PLAT300_ALERT_4_G Atom Site Occupancy of N6        Constrained at         0.5 Check
PLAT300_ALERT_4_G Atom Site Occupancy of N7        Constrained at         0.5 Check
PLAT300_ALERT_4_G Atom Site Occupancy of C11       Constrained at         0.5 Check
PLAT300_ALERT_4_G Atom Site Occupancy of C12       Constrained at         0.5 Check
PLAT300_ALERT_4_G Atom Site Occupancy of C13       Constrained at         0.5 Check
PLAT300_ALERT_4_G Atom Site Occupancy of C14       Constrained at         0.5 Check
PLAT300_ALERT_4_G Atom Site Occupancy of C15       Constrained at         0.5 Check
PLAT300_ALERT_4_G Atom Site Occupancy of C16       Constrained at         0.5 Check
PLAT300_ALERT_4_G Atom Site Occupancy of C17       Constrained at         0.5 Check
PLAT300_ALERT_4_G Atom Site Occupancy of C18       Constrained at         0.5 Check
PLAT300_ALERT_4_G Atom Site Occupancy of C19       Constrained at         0.5 Check
PLAT300_ALERT_4_G Atom Site Occupancy of C20       Constrained at         0.5 Check
PLAT300_ALERT_4_G Atom Site Occupancy of H11       Constrained at         0.5 Check
PLAT300_ALERT_4_G Atom Site Occupancy of H12       Constrained at         0.5 Check
PLAT300_ALERT_4_G Atom Site Occupancy of H14       Constrained at         0.5 Check
PLAT300_ALERT_4_G Atom Site Occupancy of H15       Constrained at         0.5 Check
PLAT300_ALERT_4_G Atom Site Occupancy of H16       Constrained at         0.5 Check
PLAT300_ALERT_4_G Atom Site Occupancy of H17       Constrained at         0.5 Check
PLAT300_ALERT_4_G Atom Site Occupancy of H19       Constrained at         0.5 Check
PLAT300_ALERT_4_G Atom Site Occupancy of H20       Constrained at         0.5 Check
PLAT302_ALERT_4_G Anion/Solvent/Minor-Residue Disorder (Resd  2  )       100% Note
PLAT789_ALERT_4_G Atoms with Negative _atom_site_disorder_group  #         20 Check
PLAT791_ALERT_4_G Model has Chirality at C2         (Sohnke SpGr)           R Verify
PLAT791_ALERT_4_G Model has Chirality at C3         (Sohnke SpGr)           S Verify
PLAT791_ALERT_4_G Model has Chirality at C5         (Sohnke SpGr)           R Verify
PLAT794_ALERT_5_G Tentative Bond Valency for Co1       (II)      .       2.02 Info
PLAT860_ALERT_3_G Number of Least-Squares Restraints .............        285 Note
PLAT870_ALERT_4_G ALERTS Related to Twinning Effects Suppressed ..          ! Info
```

---

```
 0 ALERT level A = Most likely a serious problem - resolve or explain
 0 ALERT level B = A potentially serious problem, consider carefully
11 ALERT level C = Check. Ensure it is not caused by an omission or oversight
38 ALERT level G = General information/check it is not something unexpected

 1 ALERT type 1 CIF construction/syntax error, inconsistent or missing data
10 ALERT type 2 Indicator that the structure model may be wrong or deficient
 3 ALERT type 3 Indicator that the structure quality may be low
33 ALERT type 4 Improvement, methodology, query or suggestion
 2 ALERT type 5 Informative message, check
```

---

It is advisable to attempt to resolve as many as possible of the alerts in all categories. Often the minor alerts point to easily fixed oversights, errors and omissions in your CIF or refinement strategy, so attention to these fine details can be worthwhile. In order to resolve some of the more serious problems it may be necessary to carry out additional measurements or structure refinements. However, the purpose of your study may justify the reported deviations and the more serious of these should normally be commented upon in the discussion or experimental section of a paper or in the "special_details" fields of the CIF. checkCIF was carefully designed to identify outliers and unusual parameters, but every test has its limitations and alerts that are not important in a particular case may appear. Conversely, the absence of alerts does not guarantee there are no aspects of the results needing attention. It is up to the individual to critically assess their own results and, if necessary, seek expert advice.

**Publication of your CIF in IUCr journals**

A basic structural check has been run on your CIF. These basic checks will be run on all CIFs submitted for publication in IUCr journals (*Acta Crystallographica*, *Journal of Applied Crystallography*, *Journal of Synchrotron Radiation*); however, if you intend to submit to *Acta Crystallographica Section C* or *E* or *IUCrData*, you should make sure that full publication checks are run on the final version of your CIF prior to submission.

**Publication of your CIF in other journals**

Please refer to the *Notes for Authors* of the relevant journal for any special instructions relating to CIF submission.

---

**PLATON version of 05/12/2020; check.def file version of 05/12/2020**

**Datablock 2_a** - ellipsoid plot

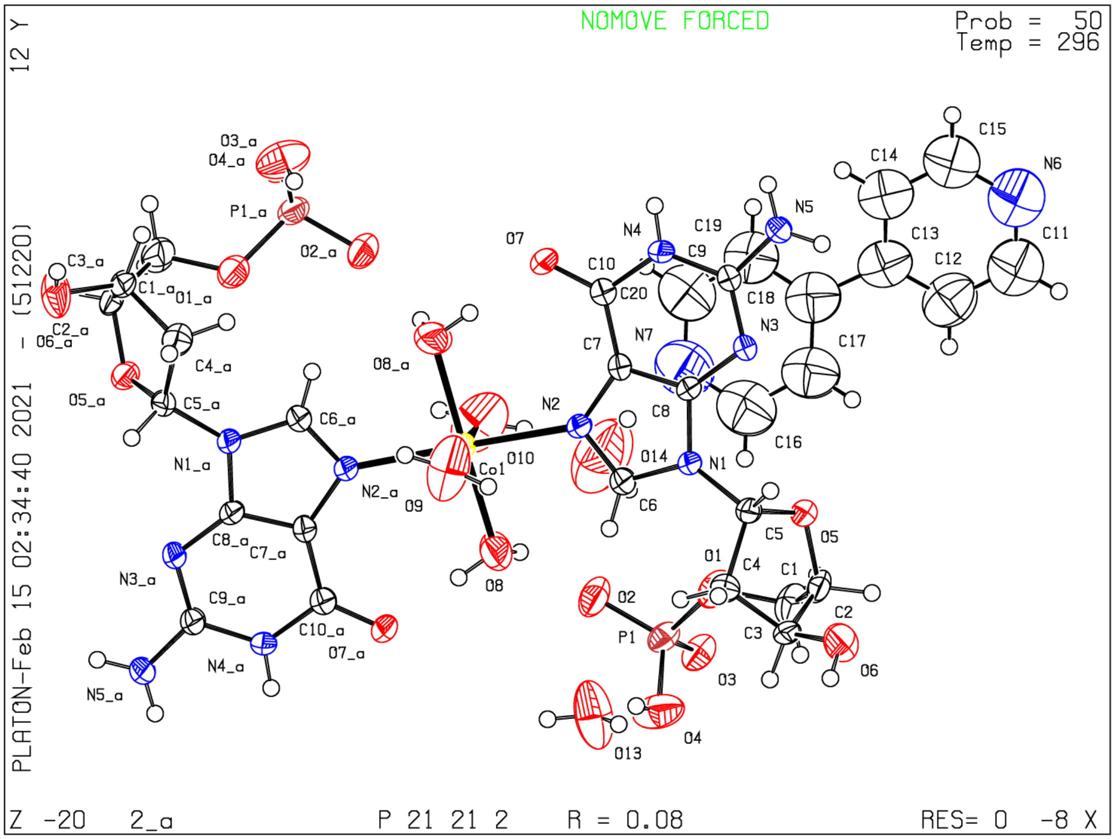

Supplement: Supplementary file 1 [file DataSheet1.ZIP › cif checkcif/Complex 1 checkcif.pdf]
