# Supplementary material for: The Research of G–Motif Construction and Chirality in Deoxyguanosine Monophosphate Nucleotide Complexes
Source: Front Chem. 2021 Jun 30;9:709777. doi: 10.3389/fchem.2021.709777 (PMC8278404; doi:10.3389/fchem.2021.709777)

checkCIF/PLATON report

You have not supplied any structure factors. As a result the full set of tests cannot be run.

THIS REPORT IS FOR GUIDANCE ONLY. IF USED AS PART OF A REVIEW PROCEDURE FOR PUBLICATION, IT SHOULD NOT REPLACE THE EXPERTISE OF AN EXPERIENCED CRYSTALLOGRAPHIC REFEREE.

No syntax errors found.   CIF dictionary   Interpreting this report

Datablock: 3_a

```
Bond precision:  C-C = 0.0081 A          Wavelength=0.71073

Cell:            a=6.9901(7)     b=20.7406(19)      c=15.2603(14)
                 alpha=90        beta=92.454(3)     gamma=90
Temperature:     296 K
```

	Calculated	Reported
Volume	2210.4(4)	2210.4(4)
Space group	P 21	P 1 21 1
Hall group	P 2yb	P 2yb
Moiety formula	C20 H34 Co N10 O18 P2, C12 H10 N2, 4(H2 O)	C20 H34 Co N10 O18 P2, 4(H2 O), C12 H10 N2
Sum formula	C32 H52 Co N12 O22 P2	C32 H52 Co N12 O22 P2
Mr	1077.73	1077.72
Dx,g cm-3	1.619	1.619
Z	2	2
Mu (mm-1)	0.559	0.559
F000	1122.0	1122.0
F000’	1123.70	
h,k,lmax	9,29,21	9,28,21
Nref	12937[6633]	12673
Tmin,Tmax	0.935,0.956	0.655,0.746
Tmin’	0.865	

```
Correction method= # Reported T Limits: Tmin=0.655 Tmax=0.746
AbsCorr = MULTI-SCAN

Data completeness= 1.91/0.98      Theta(max)= 30.040

R(reflections)= 0.0521( 8105)     wR2(reflections)= 0.1046( 12673)

S = 0.989                  Npar= 643
```

The following ALERTS were generated. Each ALERT has the format
test-name_ALERT_alert-type_alert-level.
Click on the hyperlinks for more details of the test.

Alert level C

```
PLAT234_ALERT_4_C Large Hirshfeld Difference C28      --C29      .       0.16 Ang.
PLAT260_ALERT_2_C Large Average Ueq of Residue Including       O22      0.102 Check
PLAT341_ALERT_3_C Low Bond Precision on  C-C Bonds ...............    0.00805 Ang.
PLAT417_ALERT_2_C Short Inter D-H..H-D       H6       ..H19B     .       2.14 Ang.
                                             1-x,-1/2+y,1-z  =      2_646 Check
PLAT420_ALERT_2_C D-H Without Acceptor       N5       --H5B      .     Please Check
PLAT420_ALERT_2_C D-H Without Acceptor       N10      --H10B     .     Please Check
PLAT480_ALERT_4_C Long H...A H-Bond Reported H5B      ..O12      .       2.63 Ang.
PLAT480_ALERT_4_C Long H...A H-Bond Reported H10B     ..O5       .       2.65 Ang.
```

Alert level G

```
PLAT002_ALERT_2_G Number of Distance or Angle Restraints on AtSite          6 Note
PLAT003_ALERT_2_G Number of Uiso or Uij Restrained non-H Atoms ...          7 Report
PLAT007_ALERT_5_G Number of Unrefined Donor-H Atoms ..............         26 Report
PLAT012_ALERT_1_G No      _shelx_res_checksum Found in CIF ......     Please Check
PLAT042_ALERT_1_G Calc. and Reported MoietyFormula Strings  Differ     Please Check
PLAT176_ALERT_4_G The CIF-Embedded .res File Contains SADI Records          2 Report
PLAT186_ALERT_4_G The CIF-Embedded .res File Contains ISOR Records          1 Report
PLAT187_ALERT_4_G The CIF-Embedded .res File Contains RIGU Records          1 Report
PLAT302_ALERT_4_G Anion/Solvent/Minor-Residue Disorder (Resd  2  )        14% Note
PLAT410_ALERT_2_G Short Intra H...H Contact  H22      ..H31'     .       1.95 Ang.
                                                      x,y,z  =      1_555 Check
PLAT410_ALERT_2_G Short Intra H...H Contact  H29      ..H32'     .       1.87 Ang.
                                                      x,y,z  =      1_555 Check
PLAT791_ALERT_4_G Model has Chirality at C2          (Sohnke SpGr)          R Verify
PLAT791_ALERT_4_G Model has Chirality at C3          (Sohnke SpGr)          S Verify
PLAT791_ALERT_4_G Model has Chirality at C5          (Sohnke SpGr)          R Verify
PLAT791_ALERT_4_G Model has Chirality at C12         (Sohnke SpGr)          R Verify
PLAT791_ALERT_4_G Model has Chirality at C13         (Sohnke SpGr)          S Verify
PLAT791_ALERT_4_G Model has Chirality at C15         (Sohnke SpGr)          R Verify
PLAT794_ALERT_5_G Tentative Bond Valency for Co1       (II)      .       1.92 Info
PLAT860_ALERT_3_G Number of Least-Squares Restraints ............         44 Note
PLAT870_ALERT_4_G ALERTS Related to Twinning Effects Suppressed ..          ! Info
PLAT883_ALERT_1_G No Info/Value for _atom_sites_solution_primary .     Please Do !
PLAT933_ALERT_2_G Number of OMIT Records in Embedded .res File ...          4 Note
```

---

0 **ALERT level A** = Most likely a serious problem - resolve or explain
0 **ALERT level B** = A potentially serious problem, consider carefully
8 **ALERT level C** = Check. Ensure it is not caused by an omission or oversight
22 **ALERT level G** = General information/check it is not something unexpected

3 ALERT type 1 CIF construction/syntax error, inconsistent or missing data
9 ALERT type 2 Indicator that the structure model may be wrong or deficient
2 ALERT type 3 Indicator that the structure quality may be low
14 ALERT type 4 Improvement, methodology, query or suggestion
2 ALERT type 5 Informative message, check

It is advisable to attempt to resolve as many as possible of the alerts in all categories. Often the minor alerts point to easily fixed oversights, errors and omissions in your CIF or refinement strategy, so attention to these fine details can be worthwhile. In order to resolve some of the more serious problems it may be necessary to carry out additional measurements or structure refinements. However, the purpose of your study may justify the reported deviations and the more serious of these should normally be commented upon in the discussion or experimental section of a paper or in the "special_details" fields of the CIF. checkCIF was carefully designed to identify outliers and unusual parameters, but every test has its limitations and alerts that are not important in a particular case may appear. Conversely, the absence of alerts does not guarantee there are no aspects of the results needing attention. It is up to the individual to critically assess their own results and, if necessary, seek expert advice.

Publication of your CIF in IUCr journals

A basic structural check has been run on your CIF. These basic checks will be run on all CIFs submitted for publication in IUCr journals (*Acta Crystallographica*, *Journal of Applied Crystallography*, *Journal of Synchrotron Radiation*); however, if you intend to submit to *Acta Crystallographica Section C* or *E* or *IUCrData*, you should make sure that full publication checks are run on the final version of your CIF prior to submission.

Publication of your CIF in other journals

Please refer to the *Notes for Authors* of the relevant journal for any special instructions relating to CIF submission.

PLATON version of 05/12/2020; check.def file version of 05/12/2020

Datablock 3_a - ellipsoid plot

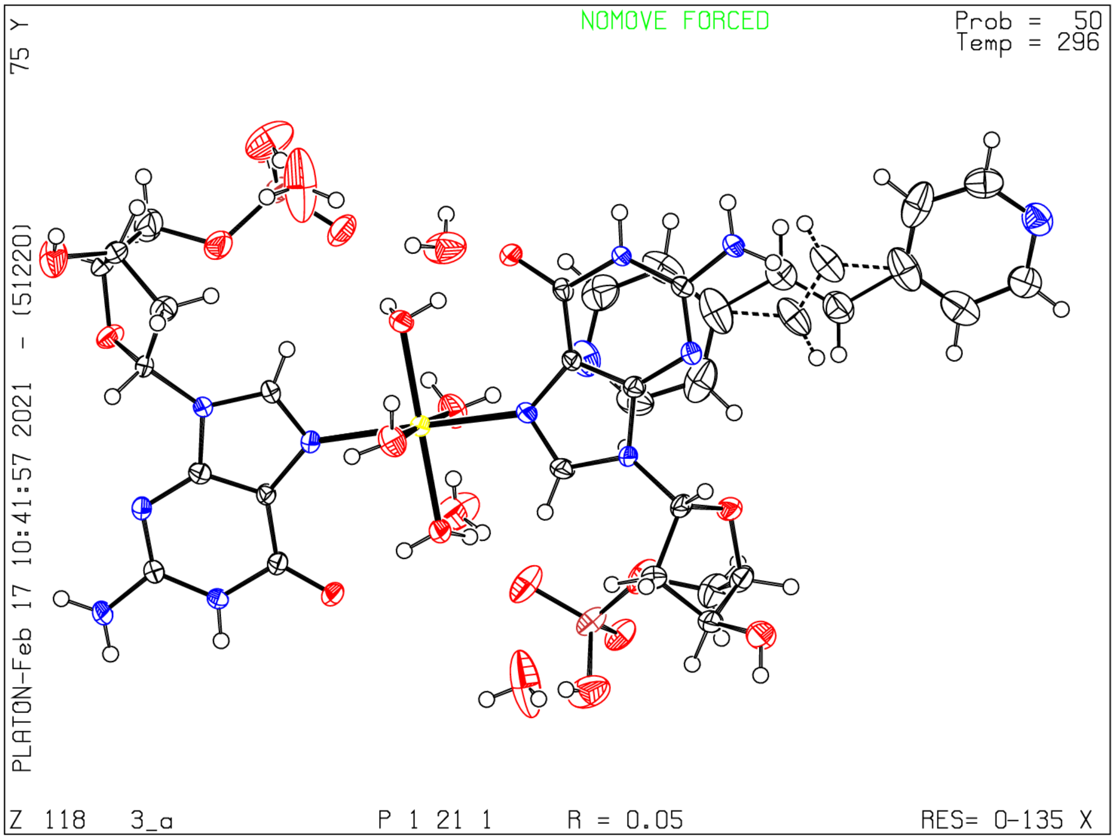

Supplement: Supplementary file 1 [file DataSheet1.ZIP › cif checkcif/Complex 2 checkcif.pdf]
