# Supplementary material for: The Research of G–Motif Construction and Chirality in Deoxyguanosine Monophosphate Nucleotide Complexes
Source: Front Chem. 2021 Jun 30;9:709777. doi: 10.3389/fchem.2021.709777 (PMC8278404; doi:10.3389/fchem.2021.709777)

checkCIF/PLATON report

You have not supplied any structure factors. As a result the full set of tests cannot be run.

THIS REPORT IS FOR GUIDANCE ONLY. IF USED AS PART OF A REVIEW PROCEDURE FOR PUBLICATION, IT SHOULD NOT REPLACE THE EXPERTISE OF AN EXPERIENCED CRYSTALLOGRAPHIC REFEREE.

No syntax errors found. CIF dictionary Interpreting this report

Datablock: 1_a

```
Bond precision:  C-C = 0.0138 A          Wavelength=0.71073

Cell:             a=15.7257(5)      b=21.0314(7)      c=6.9203(2)
                  alpha=90          beta=90           gamma=90
Temperature:      296 K
```

	Calculated	Reported
Volume	2288.78(12)	2288.78(12)
Space group	P 21 21 2	P 21 21 2
Hall group	P 2 2ab	P 2 2ab
Moiety formula	C12 H18 N4 O4 Zn, 2(C10 H13 N5 O7 P), 4(H2 O)	C12 H18 N4 O4 Zn, 2(C10 H13 N5 O7 P), 4(H2 O)
Sum formula	C32 H52 N14 O22 P2 Zn	C32 H52 N14 O22 P2 Zn
Mr	1112.21	1112.18
Dx,g cm-3	1.614	1.614
Z	2	2
Mu (mm-1)	0.705	0.705
F000	1156.0	1156.0
F000’	1157.57	
h,k,lmax	18,25,8	18,25,8
Nref	4042[2335]	4037
Tmin,Tmax	0.873,0.919	0.576,0.746
Tmin’	0.850	

```
Correction method= # Reported T Limits: Tmin=0.576 Tmax=0.746
AbsCorr = MULTI-SCAN

Data completeness= 1.73/1.00      Theta(max)= 25.015

R(reflections)= 0.0813( 3584)     wR2(reflections)= 0.2319( 4037)

S = 1.052                  Npar= 323
```

The following ALERTS were generated. Each ALERT has the format
test-name_ALERT_alert-type_alert-level.
Click on the hyperlinks for more details of the test.

Alert level C

```
PLAT090_ALERT_3_C Poor Data / Parameter Ratio (Zmax > 18) ........       7.23 Note
PLAT234_ALERT_4_C Large Hirshfeld Difference Zn1      --O1       .       0.19 Ang.
PLAT234_ALERT_4_C Large Hirshfeld Difference Zn1      --O2       .       0.19 Ang.
PLAT234_ALERT_4_C Large Hirshfeld Difference Zn1      --O3       .       0.18 Ang.
PLAT241_ALERT_2_C High   'MainMol' Ueq as Compared to Neighbors of        C10 Check
PLAT242_ALERT_2_C Low    'MainMol' Ueq as Compared to Neighbors of        Zn1 Check
PLAT242_ALERT_2_C Low    'MainMol' Ueq as Compared to Neighbors of         P1 Check
PLAT250_ALERT_2_C Large U3/U1 Ratio for Average U(i,j) Tensor ....        2.4 Note
PLAT260_ALERT_2_C Large Average Ueq of Residue Including       O11      0.129 Check
PLAT260_ALERT_2_C Large Average Ueq of Residue Including       O12      0.131 Check
PLAT341_ALERT_3_C Low Bond Precision on  C-C Bonds ..............     0.01382 Ang.
PLAT417_ALERT_2_C Short Inter D-H..H-D       H2       ..H3       .       2.14 Ang.
                                                   x,y,-1+z  =      1_554 Check
PLAT420_ALERT_2_C D-H Without Acceptor       N6       --H6B      .     Please Check
PLAT480_ALERT_4_C Long H...A H-Bond Reported H11A     ..O9       .       2.61 Ang.
PLAT480_ALERT_4_C Long H...A H-Bond Reported H11B     ..O2       .       2.64 Ang.
```

Alert level G

```
PLAT003_ALERT_2_G Number of Uiso or Uij Restrained non-H Atoms ...          3 Report
PLAT004_ALERT_5_G Polymeric Structure Found with Maximum Dimension          1 Info
PLAT007_ALERT_5_G Number of Unrefined Donor-H Atoms .............          13 Report
PLAT012_ALERT_1_G No       _shelx_res_checksum Found in CIF ......     Please Check
PLAT072_ALERT_2_G SHELXL First  Parameter in WGHT  Unusually Large       0.13 Report
PLAT083_ALERT_2_G SHELXL Second Parameter in WGHT  Unusually Large       7.21 Why ?
PLAT186_ALERT_4_G The CIF-Embedded .res File Contains ISOR Records          2 Report
PLAT794_ALERT_5_G Tentative Bond Valency for Zn1       (II)      .       2.11 Info
PLAT860_ALERT_3_G Number of Least-Squares Restraints ............          18 Note
PLAT933_ALERT_2_G Number of OMIT Records in Embedded .res File ...          2 Note
```

---

0 **ALERT level A** = Most likely a serious problem - resolve or explain
0 **ALERT level B** = A potentially serious problem, consider carefully
15 **ALERT level C** = Check. Ensure it is not caused by an omission or oversight
10 **ALERT level G** = General information/check it is not something unexpected

1 ALERT type 1 CIF construction/syntax error, inconsistent or missing data
12 ALERT type 2 Indicator that the structure model may be wrong or deficient
3 ALERT type 3 Indicator that the structure quality may be low
6 ALERT type 4 Improvement, methodology, query or suggestion
3 ALERT type 5 Informative message, check

It is advisable to attempt to resolve as many as possible of the alerts in all categories. Often the minor alerts point to easily fixed oversights, errors and omissions in your CIF or refinement strategy, so attention to these fine details can be worthwhile. In order to resolve some of the more serious problems it may be necessary to carry out additional measurements or structure refinements. However, the purpose of your study may justify the reported deviations and the more serious of these should normally be commented upon in the discussion or experimental section of a paper or in the "special_details" fields of the CIF. checkCIF was carefully designed to identify outliers and unusual parameters, but every test has its limitations and alerts that are not important in a particular case may appear. Conversely, the absence of alerts does not guarantee there are no aspects of the results needing attention. It is up to the individual to critically assess their own results and, if necessary, seek expert advice.

Publication of your CIF in IUCr journals

A basic structural check has been run on your CIF. These basic checks will be run on all CIFs submitted for publication in IUCr journals (*Acta Crystallographica*, *Journal of Applied Crystallography*, *Journal of Synchrotron Radiation*); however, if you intend to submit to *Acta Crystallographica Section C* or *E* or *IUCrData*, you should make sure that full publication checks are run on the final version of your CIF prior to submission.

Publication of your CIF in other journals

Please refer to the *Notes for Authors* of the relevant journal for any special instructions relating to CIF submission.

PLATON version of 05/12/2020; check.def file version of 05/12/2020

Datablock 1_a - ellipsoid plot

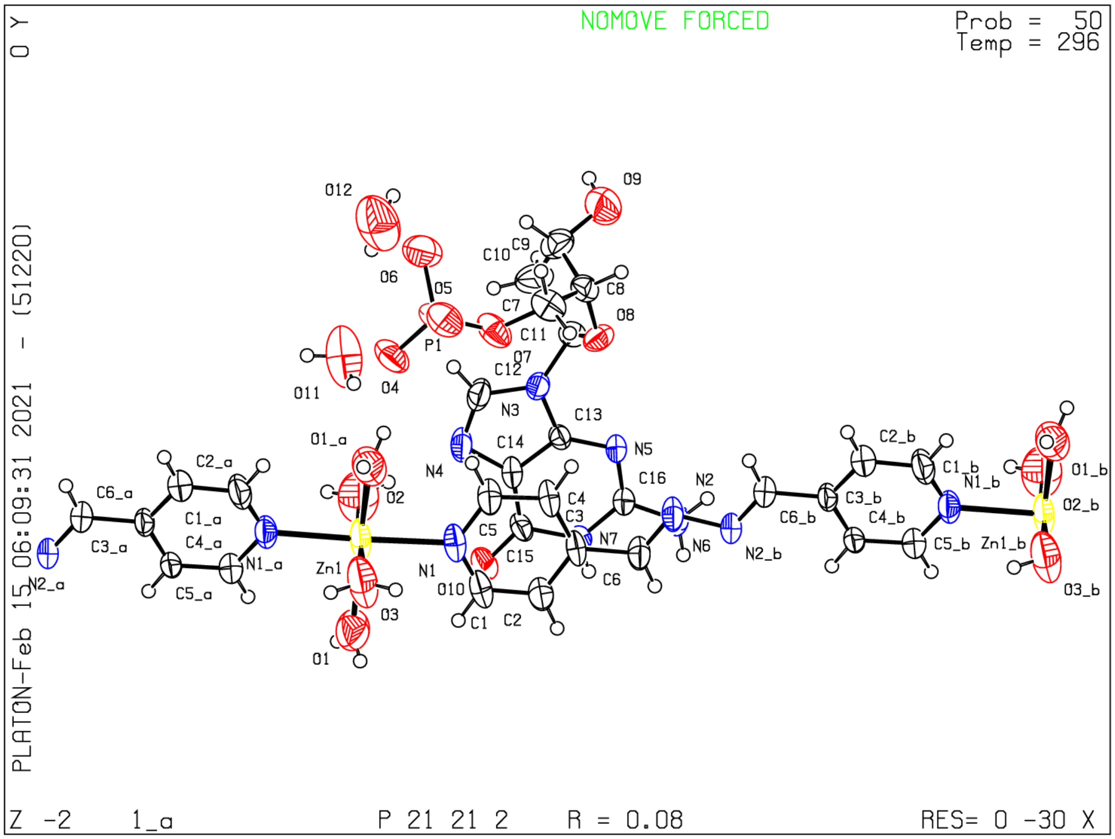

Supplement: Supplementary file 1 [file DataSheet1.ZIP › cif checkcif/Complex 4 checkcif.pdf]
