# Supplementary material for: The Research of G–Motif Construction and Chirality in Deoxyguanosine Monophosphate Nucleotide Complexes
Source: Front Chem. 2021 Jun 30;9:709777. doi: 10.3389/fchem.2021.709777 (PMC8278404; doi:10.3389/fchem.2021.709777)

checkCIF/PLATON report

You have not supplied any structure factors. As a result the full set of tests cannot be run.

THIS REPORT IS FOR GUIDANCE ONLY. IF USED AS PART OF A REVIEW PROCEDURE FOR PUBLICATION, IT SHOULD NOT REPLACE THE EXPERTISE OF AN EXPERIENCED CRYSTALLOGRAPHIC REFEREE.

No syntax errors found.   CIF dictionary   Interpreting this report

Datablock: 1_a

```
Bond precision:   C-C = 0.0065 A          Wavelength=0.71073

Cell:             a=27.735(2)     b=11.2485(11)      c=6.7722(6)
                  alpha=90        beta=92.021(8)     gamma=90
Temperature:      293 K
```

	Calculated	Reported
Volume	2111.5(3)	2111.4(3)
Space group	C 2	C 1 2 1
Hall group	C 2y	C 2y
Moiety formula	C10 H22 Mn N5 O12 P, 3(H2 O)	C10 H22 Mn N5 O12 P, 3(H2 O)
Sum formula	C10 H28 Mn N5 O15 P	C10 H28 Mn N5 O15 P
Mr	544.28	544.28
Dx,g cm-3	1.712	1.712
Z	4	4
Mu (mm-1)	0.786	0.786
F000	1132.0	1132.0
F000’	1134.44	
h,k,lmax	34,14,8	34,14,8
Nref	4511[2373]	3994
Tmin,Tmax	0.868,0.910	0.964,1.000
Tmin’	0.815	

```
Correction method= # Reported T Limits: Tmin=0.964 Tmax=1.000
AbsCorr = MULTI-SCAN

Data completeness= 1.68/0.89      Theta(max)= 26.731

R(reflections)= 0.0379( 3548)     wR2(reflections)= 0.0844( 3994)

S = 1.027                  Npar= 302
```

The following ALERTS were generated. Each ALERT has the format
test-name_ALERT_alert-type_alert-level.
Click on the hyperlinks for more details of the test.

Alert level C

```
ABSTY02_ALERT_1_C  An _exptl_absorpt_correction_type has been given without
            a literature citation. This should be contained in the
            _exptl_absorpt_process_details field.
            Absorption correction given as multi-scan
PLAT090_ALERT_3_C Poor Data / Parameter Ratio (Zmax > 18) ........       7.86 Note
PLAT341_ALERT_3_C Low Bond Precision on  C-C Bonds ...............     0.0065 Ang.
PLAT411_ALERT_2_C Short Inter H...H Contact  H4B      ..H4B      .       2.13 Ang.
                                                  1-x,y,1-z  =      2_656 Check
PLAT420_ALERT_2_C D-H Without Acceptor       N3       --H3A      .     Please Check
PLAT480_ALERT_4_C Long H...A H-Bond Reported H3A      ..O12      .       2.61 Ang.
```

Alert level G

```
PLAT002_ALERT_2_G Number of Distance or Angle Restraints on AtSite          3 Note
PLAT007_ALERT_5_G Number of Unrefined Donor-H Atoms ..............         20 Report
PLAT012_ALERT_1_G No       _shelx_res_checksum Found in CIF ......     Please Check
PLAT176_ALERT_4_G The CIF-Embedded .res File Contains SADI Records          1 Report
PLAT199_ALERT_1_G Reported _cell_measurement_temperature ..... (K)        293 Check
PLAT200_ALERT_1_G Reported   _diffrn_ambient_temperature ..... (K)        293 Check
PLAT790_ALERT_4_G Centre of Gravity not Within Unit Cell: Resd.  #          3 Note
              H2 O
PLAT791_ALERT_4_G Model has Chirality at C2          (Sohnke SpGr)          R Verify
PLAT791_ALERT_4_G Model has Chirality at C3          (Sohnke SpGr)          S Verify
PLAT791_ALERT_4_G Model has Chirality at C5          (Sohnke SpGr)          R Verify
PLAT794_ALERT_5_G Tentative Bond Valency for Mn1       (II)      .       2.03 Info
PLAT860_ALERT_3_G Number of Least-Squares Restraints .............          2 Note
PLAT883_ALERT_1_G No Info/Value for _atom_sites_solution_primary .     Please Do !
PLAT933_ALERT_2_G Number of OMIT Records in Embedded .res File ...          3 Note
```

---

0 **ALERT level A** = Most likely a serious problem - resolve or explain
0 **ALERT level B** = A potentially serious problem, consider carefully
6 **ALERT level C** = Check. Ensure it is not caused by an omission or oversight
14 **ALERT level G** = General information/check it is not something unexpected

5 ALERT type 1 CIF construction/syntax error, inconsistent or missing data
4 ALERT type 2 Indicator that the structure model may be wrong or deficient
3 ALERT type 3 Indicator that the structure quality may be low
6 ALERT type 4 Improvement, methodology, query or suggestion
2 ALERT type 5 Informative message, check

It is advisable to attempt to resolve as many as possible of the alerts in all categories. Often the minor alerts point to easily fixed oversights, errors and omissions in your CIF or refinement strategy, so attention to these fine details can be worthwhile. In order to resolve some of the more serious problems it may be necessary to carry out additional measurements or structure refinements. However, the purpose of your study may justify the reported deviations and the more serious of these should normally be commented upon in the discussion or experimental section of a paper or in the "special_details" fields of the CIF. checkCIF was carefully designed to identify outliers and unusual parameters, but every test has its limitations and alerts that are not important in a particular case may appear. Conversely, the absence of alerts does not guarantee there are no aspects of the results needing attention. It is up to the individual to critically assess their own results and, if necessary, seek expert advice.

Publication of your CIF in IUCr journals

A basic structural check has been run on your CIF. These basic checks will be run on all CIFs submitted for publication in IUCr journals (*Acta Crystallographica*, *Journal of Applied Crystallography*, *Journal of Synchrotron Radiation*); however, if you intend to submit to *Acta Crystallographica Section C* or *E* or *IUCrData*, you should make sure that full publication checks are run on the final version of your CIF prior to submission.

Publication of your CIF in other journals

Please refer to the *Notes for Authors* of the relevant journal for any special instructions relating to CIF submission.

PLATON version of 05/12/2020; check.def file version of 05/12/2020

Datablock 1_a - ellipsoid plot

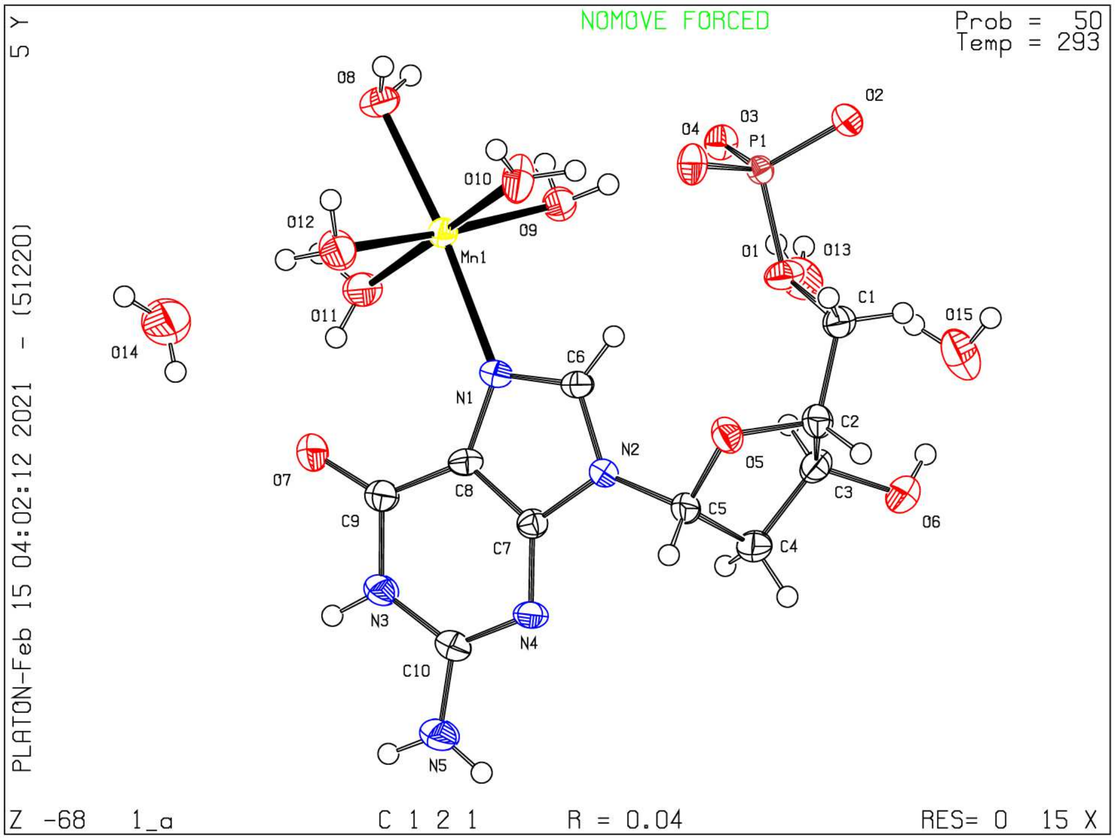

Supplement: Supplementary file 1 [file DataSheet1.ZIP › cif checkcif/Complex 5 checkcif.pdf]
