# Supplementary material for: The Research of G–Motif Construction and Chirality in Deoxyguanosine Monophosphate Nucleotide Complexes
Source: Front Chem. 2021 Jun 30;9:709777. doi: 10.3389/fchem.2021.709777 (PMC8278404; doi:10.3389/fchem.2021.709777)
Supplement: Supplementary file 2 [file DataSheet2.docx]

Supplementary Material

The Research of G–motif Construction and Chirality in Deoxyguanosine Monophosphate Nucleotide Complexes

Yanhong Zhu,^1^ Zhongkui Li,^1^ Pengfei Wang,^1^ Qi–Ming Qiu,^1^ Hongwei Ma,^2^ and Hui Li^1^*

^1^Key Laboratory of Cluster Science of Ministry of Education, School of Chemistry and Chemical Engineering, Beijing Institute of Technology, Beijing 100081, P. R. China

^2^Analytical and Testing Centre, Beijing Institute of Technology, Beijing 100081, P. R. China

*** Correspondence:**
Hui Li
lihui@bit.edu.cn

# General Methods

## Materials:

All chemical reagents were commercially available and used without further purification. Co(NO_3_)_2_·6H_2_O, Zn(NO_3_)_2_·6H_2_O, Mn(NO_3_)_2_·4H_2_O, 4,4’–bipyridine (bipy) were purchased from Adamas, 1,2–*bis*(4–pyridyl)ethane (bpe) and 1,2–*bis*(4–pyridyl)ethane (bpa) were purchased from Tci, 2'–Deoxyguanosine–5'–monophosphate disodium salt hydrate (dGMP.2Na) was purchased from Alfa Aesar.

## Instrumentation:

Elemental analyses (C, O, N, P, Mn, Co, Zn) were determined on an EA3000 elemental analyzer. FT–IR spectra were recorded on a Nicolet Nexus FT–IR spectrometer using the KBr pellet in the range of 4000–400 cm^–1^. UV–*vis* spectra were obtained from a TU–1950 spectrophotometer. Fluorescence spectra were obtained with a Hitachi F–7000 FL Spectrophotometer with a 450 W xenon lamp as the excitation source. X–ray powder diffraction studies were performed by Bruker D8 Advance X–ray diffractometer. The X–ray single crystal data collections were performed on a Bruker SMART CCD diffractometer with graphite monochromatized Mo K*α* radiation (*λ* = 0.71073 Å). CD measurements were carried out under a constant flow of nitrogen on a JASCO J–810 spectropolarimeter. Thermogravimetric analyses (TGA) were carried out using a DTG–60H thermal analyzer under nitrogen atomosphere from room temperature to 800°C with a heating rate of 5°C/min. The pH of the sample solution was measured using a PHS–3C meter.

# Synthesis and Structural Characterization

The synthesis method of all complexes is that an aqueous solution (5mL) of 2'–Deoxyguanosine–5'–monophosphate disodium salt (dGMP.2Na) was added into an aqueous solution (5 mL) of M(NO_3_)_2_ (M= Mn^2+^, Co^2+^, Zn^2+^). After stirring for 10–15 min, bridging ligand (4,4–bipy, bpe, bpa, bpda) in distilled water or ethanol (5 mL) was added to this mixture. The suspension acidity was adjusted by HNO_3_ (1 M) until the solution became clear. The resulting solution was stirred at room temperature for 20–30 min and then filtered. Single crystals suitable for X–ray diffraction analysis can be obtained by slow evaporation under room temperature. It should be noted that except for complex **5**, all other complexes were obtained in water–ethanol mixed solvent.

Powder X–ray diffraction (PXRD) patterns of polycrystalline samples of the ligands and complexes were all coincident with their theoretical ones (Supplementary Figure **25**), confirming the phase purity of the bulk samples and their isostructurality with the crystals selected for single–crystal X–ray diffraction. The water contents of the complexes and thermal stability were estimated by Thermo–Gravimetric Analysis (TGA) (Supplementary Figure **27**). Elemental analysis (C, H, N) further confirmed the chemical identity of the complexes determined by single–crystal X–ray diffraction. The types of Metal–Nucleotides interactions have been identified by the FT–IR (Supplementary Figure **26**).

## 1,4–*bis*(4–pyridyl)–2,3–diaza–1,3–butadiene (bpda)

The synthesis of **bpda** refers to the report of the Eringathodi Suresh group (Bisht et al., 2013). Pyridine–4–carbaldehyde (4.4 mL, 44 mmol) and hydrazine hydrate (2.1 mL, 22 mmol) were dissolved in 12.5 mL of ethanol, and stirred at room temperature for 24 h. The product was filtered, washed with 10 mL of ethanol/ether mixed solvent, and dried to obtain 2.88 g of yellow solid with a yield of 69%. ^1^H NMR (400 MHz, *d_6_*–DMSO) *δ* (ppm): 7.64 (d, 4H), 8.59 (s, 2H), 8.70 (d, 4H). *Anal*. Calc. (%) for C_12_H_10_N_4_: C, 22.06; H, 5.19; N, 12.86. Found (%): C, 22.05; H, 5.22; N, 12.94. IR (KBr pallet, cm^–1^): *ν*: 1683m, 1597s, 1556m, 1409m; 1301m, 1223m, 1197w, 1067w, 1050w, 987s, 963m, 807vs, 668s, 502s, 478m.

## [Co(HdGMP)_2_(H_2_O)_4_]·(4,4′–bipy)·3H_2_O (1)

An aqueous solution (5 mL) of 2'–Deoxyguanosine–5'–monophosphate disodium salt hydrate (dGMP.2Na, 39 mg, 0.10 mmol) was added into an aqueous solution (5 mL) of Co(NO_3_)_2_· 6H_2_O (15 mg, 0.05 mmol). After stirring for 10 min, 4,4’–bipyridine (bipy, 8mg, 0.05 mmol) in ethanol solution (5 mL) was added to this mixture. The suspension acidity was adjusted by HNO_3_ (1 M) until the solution became clear (pH = 5.78). The resulting solution was stirred at room temperature for 30 min and then filtered. The pink stick crystals were obtained by evaporation at room temperature after two days. Yield 82 %. *Anal*. Calc. (%) for C_30_H_48_CoN_12_O_21_P_2_: C, 34.86; H, 4.68; N, 16.25. Found (%): C, 34.98; H, 4.92; N, 15.99. IR (KBr pallet, cm^–1^): *v*: 3381s, 1650vs, 1535w, 1490w, 1411w, 1178w, 1069m, 999w, 980w, 931w, 811w, 784w, 734w, 669w, 617w, 518w, 488w.

## [Co(HdGMP)_2_(H_2_O)_4_]·(bpe)·4H_2_O (2)

Complex **2** was prepared in a manner similar to that described for **1**, just using 1,2–*bis*(4–pyridyl)ethene (bpe, 9 mg, 0.05 mmol) instead of bipy (pH = 5.83). After five days, the pink stick crystals were obtained. Yield 70%. *Anal*. Calc. (%) for C_32_H_52_CoN_12_O_22_P_2_: C, 35.66; H, 4.86; N, 15.59. Found (%): C, 35.58; H, 4.66; N, 15.47. IR (KBr pallet, cm^–1^): *v*: 3447vs, 1697m, 1653s, 1575w, 1534w, 1490w, 1452w, 1409w, 1249w, 1179w, 1070w, 1054w, 1007w, 979w, 931w, 812w, 782w, 733w, 699w, 588w, 516w, 487w.

## [Co(HdGMP)_2_(H_2_O)_4_]·(bpa)·4H_2_O (3)

Complex **3** was prepared in a manner similar to that described for **1**, using 1,2–*bis*(4–pyridyl)ethane (bpa, 9 mg, 0.05 mmol) instead of bipy (pH = 5.76). After two weeks, the pink stick crystals were obtained. Yield 55%. *Anal*. Calc. (%) for C_32_H_54_CoN_12_O_22_P_2_: C, 35.59; H, 5.04; N, 15.66. Found (%): C, 35.61; H, 4.89; N, 15.49. *v*: 3160s, 1649vs, 1600m, 1576w, 1536w, 1491w, 1421w, 1221w, 1071w, 1054w, 1005w, 932w, 917w, 829w, 784w, 735w, 699w, 589w, 517w, 488w.

## {[Zn(bpda)(H_2_O)_4_]·(HdGMP)_2_·4H_2_O}*_n_* (4)

An aqueous solution (5 mL) of 2'–Deoxyguanosine–5'–monophosphate disodium salt hydrate (dGMP.2Na, 39 mg, 0.10 mmol) was added into an aqueous solution (5 mL) of Zn(NO_3_)_2_· 6H_2_O (15 mg, 0.05 mmol). After stirring for 10 min, 1,4–*bis*(4–pyridyl)–2,3–diaza–1,3–butadiene (bpda, 11mg, 0.05 mmol) in ethanol solution (5 mL) was added to this mixture. The suspension acidity was adjusted by HNO_3_ (1 M) until the solution became clear (pH = 5.88). The resulting solution was stirred at room temperature for 30 min and then filtered. The yellow block crystals were obtained by evaporation at room temperature after two days. Yield 80%. *Anal*. Calc. (%) for C_32_H_52_N_14_O_22_P_2_Zn: C, 34.56; H, 4.71; N, 17.62. Found (%): C, 34.47; H, 4.56; N, 17.50. *v*: 3405s, 1695m, 1652vs, 1611w, 1577w, 1534w, 1483w, 1418w, 1374w, 1237w, 1177w, 1064m, 1016w, 989w, 931w, 830w, 782w, 734w, 690w, 512w.

## [Mn(dGMP) (H_2_O)_5_]·3H_2_O (5)

An aqueous solution (5 mL) of 2'–Deoxyguanosine–5'–monophosphate disodium salt hydrate (dGMP.2Na, 20 mg, 0.05 mmol) was added into an aqueous solution (5 mL) of Mn(NO_3_)_2_· 4H_2_O (12 mg, 0.05 mmol). After stirring for 10 min, 1,4–*bis*(4–pyridyl)–2,3–diaza–1,3–butadiene (bpda, 11 mg, 0.05 mmol) in distilled water (5 mL) was added to this mixture. The resulting solution was stirred at room temperature for 30 min and then filtered (pH = 6.03). The colorless block crystals were obtained by evaporation at room temperature after one week. Yield 95 %. Change the auxiliary ligand to 4,4’–bipyridine (bipy), 1,2–*bis*(4–pyridyl)ethene (bpe), 1,2–*bis*(4–pyridyl)ethane (bpa) or no auxiliary ligand, the results are the same. *Anal*. Calc. (%) for C_10_H_28_N_5_O_15_PMn: C, 68.60; H, 4.80; N, 26.70. Found (%): C, 68.46; H, 4.82; N, 26.27. IR (KBr pallet, cm^–1^): *v*: 3421vs, 1688m, 1654s, 1567w, 1533w, 1475w, 1385m, 1271w, 1211w, 1178w, 1080s, 979m, 956w, 894w, 871w, 832w, 801w, 775w, 703w, 668w, 532w.

# Crystal Structure Determination and Refinement

The X–ray single crystal data collections for the five complexes were performed on a Bruker APEX–II CCD and Rigaku Saturn724+ (2 x 2 bin mode) diffractometer with graphite monochromatized MoKα radiation (*λ* = 0.71073 Å). The crystal sizes of the complexes are 0.26 × 0.16 × 0.13 mm^3^ **(1)**, 0.26 × 0.1 × 0.08 mm^3^ **(2)**, 0.26 × 0.15 × 0.12 mm^3^ **(3)**, 0.23 × 0.16 × 0.12 mm^3^ **(4)**, 0.26 × 0.15 × 0.12 mm^3^ **(5)**. The crystals were kept at 296.15 K or 293(2) K during data collection. Using Olex 2 (Dolomanov et al., 2009), the structure was solved with the XT (Sheldrick, 2015). Structure solution program using Intrinsic Phasing and refined with the SHELXL (Sheldrick, 2015) and refinement package using Least Squares minimisation. All non–hydrogen atoms in the complexes were refined anisotropically. The hydrogen atoms bound to carbon, nitrogen or phosphorus located by geometrical calculations, and their positions and thermal parameters were fixed during structure refinement. The hydrogen atoms belonging to water molecules were placed in their geometrically generated positions. Among them, in **1**, the free 4,4’–bipyridine ligand has disordered orientation. The occupancy ratio of each atom (C11, C12, C13, C14, C15, C16, C17, C18, C19, C20, N6, N7) in 4,4’–bipyridine is 0.5; the vinyl groups of the 1,2–*bis*(4–pyridyl)ethane in **2** are disordered, and the ratio of the occupancy rates of C31 and C32 to C31' and C32' is 0.697(13): 0.303(13); the ethyl groups in the 1,2–*bis*(4–pyridyl)ethane (bpa) of the **3** are also disordered, and the occupancy ratio of C31 and C32 to C31' and C32' is 0.642(17): 0.358(17). Further crystallographic data and experimental details for structural analyses of all complexes are summarized in Supplementary Table **1**, and selected bond distances and angles with their estimated standard deviations for complexes **1**–**5** are summarized in Tables S2–S6. The hydrogen bonds of complexes **1–5** are summarized in Supplementary Table **7–11**.

# Crystallographic Data and Structural Information

**Supplementary Table 1.** Crystallographic data for complexes **1‒5**.

| **Complex** | **Complex 1** | **Complex 2** | **Complex 3** | **Complex 4** | **Complex 5** |
| --- | --- | --- | --- | --- | --- |
| Formula | C_30_H_50_CoN_12_O_22_P_2_ | C_32_H_52_CoN_12_O_22_P_2_ | C_32_H_54_CoN_12_O_22_P_2_ | C_32_H_52_N_14_O_22_P_2_Zn | C_10_H_28_MnN_5_O_15_P |
| *Mr* | 1051.69 | 1077.72 | 1079.74 | 1112.18 | 544.28 |
| Crystal system | Orthorhombic | Monoclinic | Monoclinic | Orthorhombic | Monoclinic |
| Space group | *P*2_1_2_1_2 | *P*2_1_ | *P*2_1_ | *P*2_1_2_1_2 | *C*2 |
| *a* (Å) | 15.2094(9) | 6.9901(7) | 7.0917(2) | 15.7257(5) | 27.735(2) |
| *b* (Å) | 20.6549(12) | 20.7406(19) | 20.6519(7) | 21.0314(7) | 11.2485(11) |
| *c* (Å) | 7.0696(4) | 15.2603(14) | 15.2533(5) | 6.9203(2) | 6.7722(6) |
| *α* (°) | 90 | 90 | 90 | 90 | 90 |
| *β* (°) | 90 | 92.454(3) | 92.7700(10) | 90 | 92.021(8) |
| *γ* (°) | 90 | 90 | 90 | 90 | 90 |
| *V*(Å^3^) | 2220.9(2) | 2210.4(4) | 2231.34(12) | 2288.78(12) | 2111.4(3) |
| *Z* | 2 | 2 | 2 | 2 | 4 |
| F(000) | 1094.0 | 1122.0 | 1126.0 | 1156.0 | 1132.0 |
| Reflections collected | 24804 | 30266 | 21882 | 22389 | 8544 |
| Independent reflections | 4711 | 12673 | 7887 | 4037 | 3994 |
| Goodness–of–fit on *F*^2^ | 1.093 | 0.976 | 1.024 | 1.052 | 1.027 |
| Completeness to 2θ | 53.46, 100.0% | 60.08, 99.9% | 50.04, 99.9% | 50.03, 99.9% | 53.46, 99.5% |
| *R*_int_ | 0.0341 | 0.0564 | 0.0263 | 0.0270 | 0.0461 |
| *R*_1_[*I*>2σ(*І*)] | 0.0760 | 0.0516 | 0.0319 | 0.0813 | 0.0379 |
| *wR*_2_[*I*>2σ(*І*)] | 0.2264 | 0.0865 | 0.0799 | 0.2224 | 0.0806 |
| *R*_1_(all data) | 0.0855 | 0.1052 | 0.0371 | 0.0903 | 0.0450 |
| *wR*_2_(all data) | 0.2372 | 0.1029 | 0.0826 | 0.2319 | 0.0844 |
| Flack Parameter | 0.02(5) | 0.012(18) | 0.011(15) | 0.015(7) | –0.02(2) |
| **CCDC Nu.** | **2064323** | **2064324** | **2064325** | **2064326** | **2064327** |

**Supplementary Table 2.** Selected bond distances (Å) and angles (°) for **Complex 1**

| \| Co1– \| O8^1^ \| \| --- \| --- \| | 2.074(7) | \| Co1 \| –O8 \| \| --- \| --- \| | 2.074(7) |
| --- | --- | --- | --- | --- | --- | --- | --- |
| \| Co1– \| O9 \| \| --- \| --- \| | 2.005(12) | \| Co1 \| –O10 \| \| --- \| --- \| | 2.053(16) |
| \| Co1 \| –N2^1^ \| \| --- \| --- \| | 2.226(6) | \| Co1– \| N2 \| \| --- \| --- \| | 2.226(6) |
| \| P1– \| O1 \| \| --- \| --- \| | 1.588(7) | \| P1 \| –O4 \| \| --- \| --- \| | 1.567(8) |
| \| P1 \| –O3 \| \| --- \| --- \| | 1.494(6) | \| P1 \| –O2 \| \| --- \| --- \| | 1.481(6) |
|  |  |  |  |
| \| O8^1^ \| –Co1– \| O8 \| \| --- \| --- \| --- \| | 174.9(6) | \| O8^1^ \| –Co1 \| –N2^1^ \| \| --- \| --- \| --- \| | 85.9(2) |
| \| O8 \| –Co1 \| –N2^1^ \| \| --- \| --- \| --- \| | 94.4(2) | \| O8^1^ \| –Co1 \| –N2 \| \| --- \| --- \| --- \| | 94.4(2) |
| \| O8 \| –Co1 \| –N2 \| \| --- \| --- \| --- \| | 85.9(2) | \| O9 \| –Co1 \| –O8 \| \| --- \| --- \| --- \| | 92.5(3) |
| \| O9 \| –Co1 \| –O8^1^ \| \| --- \| --- \| --- \| | 92.5(3) | \| O9 \| –Co1 \| –O10 \| \| --- \| --- \| --- \| | 180.0 |
| \| O9 \| –Co1 \| –N2 \| \| --- \| --- \| --- \| | 86.5(2) | \| O9 \| –Co1 \| –N2^1^ \| \| --- \| --- \| --- \| | 86.5(2) |
| \| O10 \| –Co1 \| –O8 \| \| --- \| --- \| --- \| | 87.5(3) | \| O10 \| –Co1 \| –O8^1^ \| \| --- \| --- \| --- \| | 87.5(3) |
| \| O10 \| –Co1 \| –N2 \| \| --- \| --- \| --- \| | 93.5(2) | \| O10 \| –Co1 \| –N2^1^ \| \| --- \| --- \| --- \| | 93.5(2) |
| \| N2 \| –Co1 \| –N2^1^ \| \| --- \| --- \| --- \| | 173.0(4) | \| O4 \| –P1 \| –O1 \| \| --- \| --- \| --- \| | 104.4(5) |
| \| O3 \| –P1 \| –O1 \| \| --- \| --- \| --- \| | 111.0(4) | \| O3 \| –P1 \| –O4 \| \| --- \| --- \| --- \| | 106.3(4) |
| \| O2 \| –P1 \| –O1 \| \| --- \| --- \| --- \| | 104.5(4) | \| O2– \| P1 \| –O4 \| \| --- \| --- \| --- \| | 113.3(5) |
| \| O2 \| –P1 \| –O3 \| \| --- \| --- \| --- \| | 116.7(4) |  |  |

Symmetry transformations used to generate equivalent atoms: ^1^1–X, 1–Y, +Z

**Supplementary Table 3.** Selected bond distances (Å) and angles (°) for **Complex 2**

| \| Co1 \| –O15 \| \| --- \| --- \| | 2.092(4) | \| Co1 \| –O16 \| \| --- \| --- \| | 2.102(4) |
| --- | --- | --- | --- | --- | --- | --- | --- |
| \| Co1 \| –O17 \| \| --- \| --- \| | 2.041(3) | \| Co1 \| –O18 \| \| --- \| --- \| | 2.064(3) |
| \| Co1 \| –N2 \| \| --- \| --- \| | 2.242(4) | \| Co1 \| –N7 \| \| --- \| --- \| | 2.226(4) |
| \| P1 \| –O1 \| \| --- \| --- \| | 1.595(4) | \| P1 \| –O2 \| \| --- \| --- \| | 1.481(4) |
| \| P1 \| –O3 \| \| --- \| --- \| | 1.498(4) | \| P1 \| –O4 \| \| --- \| --- \| | 1.565(4) |
| \| P2 \| –O8 \| \| --- \| --- \| | 1.596(4) | \| P2 \| –O9 \| \| --- \| --- \| | 1.487(4) |
| \| P2 \| –O10 \| \| --- \| --- \| | 1.561(4) | \| P2 \| –O11 \| \| --- \| --- \| | 1.495(4) |
|  |  |  |  |
| \| O15 \| –Co1 \| –O16 \| \| --- \| --- \| --- \| | 179.17(16) | \| O15 \| –Co1 \| –N2 \| \| --- \| --- \| --- \| | 91.26(14) |
| \| O15 \| –Co1 \| –N7 \| \| --- \| --- \| --- \| | 88.92(14) | \| O16 \| –Co1 \| –N2 \| \| --- \| --- \| --- \| | 88.76(14) |
| \| O16 \| –Co1 \| –N7 \| \| --- \| --- \| --- \| | 91.18(14) | \| O17 \| –Co1 \| –O15 \| \| --- \| --- \| --- \| | 88.74(16) |
| \| O17 \| –Co1 \| –O16 \| \| --- \| --- \| --- \| | 90.43(16) | \| O17 \| –Co1 \| –O18 \| \| --- \| --- \| --- \| | 177.64(15) |
| \| O17 \| –Co1 \| –N2 \| \| --- \| --- \| --- \| | 91.36(14) | \| O17 \| –Co1 \| –N7 \| \| --- \| --- \| --- \| | 96.85(14) |
| \| O18 \| –Co1 \| –O15 \| \| --- \| --- \| --- \| | 91.00(15) | \| O18 \| –Co1 \| –O16 \| \| --- \| --- \| --- \| | 89.83(15) |
| \| O18 \| –Co1 \| –N2 \| \| --- \| --- \| --- \| | 86.30(14) | \| O18 \| –Co1 \| –N7 \| \| --- \| --- \| --- \| | 85.50(14) |
| \| N7 \| –Co1 \| –N2 \| \| --- \| --- \| --- \| | 171.79(15) | \| O2 \| –P1 \| –O1 \| \| --- \| --- \| --- \| | 104.6(2) |
| \| O2 \| –P1 \| –O3 \| \| --- \| --- \| --- \| | 117.3(2) | \| O2 \| –P1 \| –O4 \| \| --- \| --- \| --- \| | 112.8(2) |
| \| O3 \| –P1 \| –O1 \| \| --- \| --- \| --- \| | 110.1(2) | \| O3 \| –P1 \| –O4 \| \| --- \| --- \| --- \| | 106.5(2) |
| \| O4 \| –P1 \| –O1 \| \| --- \| --- \| --- \| | 104.9(2) | \| O9 \| –P2 \| –O8 \| \| --- \| --- \| --- \| | 104.8(2) |
| \| O9 \| –P2 \| –O10 \| \| --- \| --- \| --- \| | 113.0(2) | \| O9 \| –P2 \| –O11 \| \| --- \| --- \| --- \| | 118.3(2) |
| \| O10 \| –P2 \| –O8 \| \| --- \| --- \| --- \| | 105.6(3) | \| O11 \| –P2 \| –O8 \| \| --- \| --- \| --- \| | 109.5(2) |
| \| O11 \| –P2 \| –O10 \| \| --- \| --- \| --- \| | 104.9(2) |  |  |

**Supplementary Table 4.** Selected bond distances (Å) and angles (°) for **Complex 3**

| \| Co1 \| –O15 \| \| --- \| --- \| | 2.090(3) | \| Co1 \| –O18 \| \| --- \| --- \| | 2.069(3) |
| --- | --- | --- | --- | --- | --- | --- | --- |
| \| Co1 \| –O17 \| \| --- \| --- \| | 2.085(3) | \| Co1 \| –O16 \| \| --- \| --- \| | 2.054(3) |
| \| Co1 \| –N2 \| \| --- \| --- \| | 2.237(3) | \| Co1 \| –N7 \| \| --- \| --- \| | 2.230(3) |
| \| P1 \| –O1 \| \| --- \| --- \| | 1.595(3) | \| P1 \| –O3 \| \| --- \| --- \| | 1.494(3) |
| \| P1 \| –O4 \| \| --- \| --- \| | 1.559(4) | \| P1 \| –O2 \| \| --- \| --- \| | 1.475(3) |
| \| P2 \| –O8 \| \| --- \| --- \| | 1.594(3) | \| P2 \| –O10 \| \| --- \| --- \| | 1.492(3) |
| \| P2 \| –O11 \| \| --- \| --- \| | 1.565(4) | \| O9 \| –P2 \| \| --- \| --- \| | 1.482(3) |
|  |  |  |  |
| \| O15 \| –Co1 \| –N2 \| \| --- \| --- \| --- \| | 88.47(12) | \| O15 \| –Co1 \| –N7 \| \| --- \| --- \| --- \| | 91.83(12) |
| \| O18 \| –Co1 \| –O15 \| \| --- \| --- \| --- \| | 90.20(13) | \| O18 \| –Co1 \| –O17 \| \| --- \| --- \| --- \| | 90.90(13) |
| \| O18 \| –Co1 \| –N2 \| \| --- \| --- \| --- \| | 86.45(12) | \| O18 \| –Co1 \| –N7 \| \| --- \| --- \| --- \| | 85.42(11) |
| \| O17 \| –Co1 \| –O15 \| \| --- \| --- \| --- \| | 178.88(15) | \| O17 \| –Co1 \| –N2 \| \| --- \| --- \| --- \| | 91.39(12) |
| \| O17 \| –Co1 \| –N7 \| \| --- \| --- \| --- \| | 88.47(12) | \| O16 \| –Co1 \| –O15 \| \| --- \| --- \| --- \| | 89.78(14) |
| \| O16 \| –Co1 \| –O18 \| \| --- \| --- \| --- \| | 177.99(12) | \| O16 \| –Co1– \| O17 \| \| --- \| --- \| --- \| | 89.11(14) |
| \| O16 \| –Co1 \| –N2 \| \| --- \| --- \| --- \| | 91.54(12) | \| O16 \| –Co1– \| N7 \| \| --- \| --- \| --- \| | 96.59(12) |
| \| N7 \| –Co1 \| –N2 \| \| --- \| --- \| --- \| | 171.86(13) | \| O3 \| –P1 \| –O1 \| \| --- \| --- \| --- \| | 110.45(19) |
| \| O3 \| –P1 \| –O4 \| \| --- \| --- \| --- \| | 105.8(2) | \| O4 \| –P1– \| O1 \| \| --- \| --- \| --- \| | 104.7(2) |
| \| O2– \| P1– \| O1 \| \| --- \| --- \| --- \| | 104.58(19) | \| O2– \| P1– \| O3 \| \| --- \| --- \| --- \| | 117.42(19) |
| \| O2 \| –P1– \| O4 \| \| --- \| --- \| --- \| | 113.2(2) | \| O9 \| –P2 \| –O8 \| \| --- \| --- \| --- \| | 104.84(18) |
| \| O9 \| –P2 \| –O10 \| \| --- \| --- \| --- \| | 118.45(18) | \| O9– \| P2 \| –O11 \| \| --- \| --- \| --- \| | 112.8(2) |
| \| O10– \| P2 \| –O8 \| \| --- \| --- \| --- \| | 109.71(19) | \| O10 \| –P2 \| –O11 \| \| --- \| --- \| --- \| | 104.9(2) |
| \| O11 \| –P2 \| –O8 \| \| --- \| --- \| --- \| | 105.4(2) |  |  |

**Supplementary Table 5.** Selected bond distances (Å) and angles (°) for **Complex 4**

| \| Zn1 \| –O3 \| \| --- \| --- \| | 1.976(15) | \| Zn1 \| –O1^1^ \| \| --- \| --- \| | 2.221(10) |
| --- | --- | --- | --- | --- | --- | --- | --- |
| \| Zn1 \| –O1 \| \| --- \| --- \| | 2.221(10) | \| Zn1 \| –O2 \| \| --- \| --- \| | 1.925(18) |
| \| Zn1 \| –N1^1^ \| \| --- \| --- \| | 2.183(7) | \| Zn1 \| –N1 \| \| --- \| --- \| | 2.183(7) |
| \| P1 \| –O6 \| \| --- \| --- \| | 1.568(9) | \| P1 \| –O5 \| \| --- \| --- \| | 1.480(9) |
| \| P1 \|  \| –O4 \| \| --- \| --- \| --- \| | 1.470(8) | \| P1 \| –O7 \| \| --- \| --- \| | 1.582(8) |
|  |  |  |  |
| \| O3 \| –Zn1 \| –O1^1^ \| \| --- \| --- \| --- \| | 79.3(4) | \| O3 \| –Zn1 \| –O1 \| \| --- \| --- \| --- \| | 79.3(4) |
| \| O3 \| –Zn1– \| N1^1^ \| \| --- \| --- \| --- \| | 92.0(2) | \| O3 \| –Zn1 \| –N1 \| \| --- \| --- \| --- \| | 92.0(2) |
| \| O1 \| –Zn1 \| –O1^1^ \| \| --- \| --- \| --- \| | 158.6(7) | \| O2 \| –Zn1 \| –O3 \| \| --- \| --- \| --- \| | 180.0 |
| \| O2 \| –Zn1 \| –O1 \| \| --- \| --- \| --- \| | 100.7(4) | \| O2 \| –Zn1 \| –O1^1^ \| \| --- \| --- \| --- \| | 100.7(4) |
| \| O2 \| –Zn1 \| –N1 \| \| --- \| --- \| --- \| | 88.0(2) | \| O2 \| –Zn1 \| –N1^1^ \| \| --- \| --- \| --- \| | 88.0(2) |
| \| N1 \| –Zn1 \| –O1^1^ \| \| --- \| --- \| --- \| | 90.1(3) | \| N1 \| –Zn1 \| –O1 \| \| --- \| --- \| --- \| | 90.7(3) |
| \| N1^1^ \| –Zn1 \| –O1^1^ \| \| --- \| --- \| --- \| | 90.7(3) | \| N1^1^ \| –Zn1 \| –O1 \| \| --- \| --- \| --- \| | 90.1(3) |
| \| N1^1^ \| –Zn1 \| –N1 \| \| --- \| --- \| --- \| | 176.1(5) | \| O6 \| –P1 \| –O7 \| \| --- \| --- \| --- \| | 105.1(5) |
| \| O5 \| –P1 \| –O6 \| \| --- \| --- \| --- \| | 105.7(5) | \| O5 \| –P1 \| –O7 \| \| --- \| --- \| --- \| | 109.9(5) |
| \| O4 \| –P1 \| –O6 \| \| --- \| --- \| --- \| | 112.4(6) | \| O4 \| –P1 \| –O5 \| \| --- \| --- \| --- \| | 117.2(5) |
| \| O4 \| –P1 \| –O7 \| \| --- \| --- \| --- \| | 105.9(5) |  |  |

Symmetry transformations used to generate equivalent atoms: ^1^1–X, 1–Y, +Z

**Supplementary Table 6.** Selected bond distances (Å) and angles (°) for **Complex 5.**

| \| Mn1 \| –N1 \| \| --- \| --- \| | 2.253(4) | \| Mn1 \| –O9 \| \| --- \| --- \| | 2.200(3) |
| --- | --- | --- | --- | --- | --- | --- | --- |
| \| Mn1 \| –O11 \| \| --- \| --- \| | 2.169(3) | \| Mn1 \| –O10 \| \| --- \| --- \| | 2.143(3) |
| \| Mn1 \| –O8 \| \| --- \| --- \| | 2.198(3) | \| Mn1 \| –O12 \| \| --- \| --- \| | 2.254(3) |
| \| P1 \| –O2 \| \| --- \| --- \| | 1.519(3) | \| P1 \| –O3 \| \| --- \| --- \| | 1.515(3) |
| \| P1 \| –O4 \| \| --- \| --- \| | 1.518(3) | \| P1 \| –O1 \| \| --- \| --- \| | 1.608(3) |
|  |  |  |  |
| \| N1 \| –Mn1 \| –O12 \| \| --- \| --- \| --- \| | 91.53(14) | \| O9 \| –Mn1 \| –N1 \| \| --- \| --- \| --- \| | 89.74(13) |
| \| O9 \| –Mn1 \| –O12 \| \| --- \| --- \| --- \| | 170.19(13) | \| O11 \| –Mn1 \| –N1 \| \| --- \| --- \| --- \| | 86.82(13) |
| \| O11 \| –Mn1 \| –O9 \| \| --- \| --- \| --- \| | 93.47(13) | \| O11 \| –Mn1 \| –O8 \| \| --- \| --- \| --- \| | 86.70(13) |
| \| O11 \| –Mn1 \| –O12 \| \| --- \| --- \| --- \| | 96.31(14) | \| O10 \| –Mn1 \| –N1 \| \| --- \| --- \| --- \| | 92.58(15) |
| \| O10 \| –Mn1 \| –O9 \| \| --- \| --- \| --- \| | 88.95(13) | \| O10 \| –Mn1 \| –O11 \| \| --- \| --- \| --- \| | 177.50(15) |
| \| O10 \| –Mn1 \| –O8 \| \| --- \| --- \| --- \| | 93.85(15) | \| O10 \| –Mn1 \| –O12 \| \| --- \| --- \| --- \| | 81.28(13) |
| \| O8 \| –Mn1 \| –N1 \| \| --- \| --- \| --- \| | 173.43(14) | \| O8 \| –Mn1 \| –O9 \| \| --- \| --- \| --- \| | 91.74(12) |
| \| O8 \| –Mn1 \| –O12 \| \| --- \| --- \| --- \| | 88.10(14) | \| O2 \| –P1 \| –O1 \| \| --- \| --- \| --- \| | 108.19(19) |
| \| O3 \| –P1 \| –O2 \| \| --- \| --- \| --- \| | 112.99(18) | \| O3 \| –P1 \| –O4 \| \| --- \| --- \| --- \| | 113.54(19) |
| \| O3 \| –P1 \| –O1 \| \| --- \| --- \| --- \| | 102.46(17) | \| O4 \| –P1 \| –O2 \| \| --- \| --- \| --- \| | 111.35(17) |
| \| O4 \| –P1 \| –O1 \| \| --- \| --- \| --- \| | 107.65(19) |  |  |

**Supplementary Table 7.** Selected H–bonding distances (Å) and angles (°) for **Complex 1**

| **D–H** | **A** | **d(H···A)/Å** | **d(D···A)/Å** | **∠DHA /°** | **Symmetry** |
| --- | --- | --- | --- | --- | --- |
| O6–H6 | O7 | 1.98 | 2.773(9) | 161.1 | [3/2–X,–1/2+Y,2–Z] |
| O4–H4 | O13 | 1.74 | 2.551(12) | 167.1 |  |
| O8–H8A | O7 | 1.72 | 2.572(8) | 176.1 | [1–X,1–Y,+Z] |
| O8–H8B | O2 | 1.93 | 2.703(10) | 148.9 |  |
| O9–H9 | O14 | 1.97 | 2.748(18) | 151.2 | [1–X,1–Y,1+Z] |
| O10–H10 | O14 | 1.88 | 2.673(19) | 154.6 | [1–X,1–Y,+Z] |
| N4–H4A | O3 | 1.85 | 2.707(8) | 172.5 | [3/2–X,1/2+Y,1–Z] |
| N5–H5A | N3 | 2.12 | 2.972(9) | 169.8 | [2–X,1–Y,+Z] |

**Supplementary Table 8.** Selected H–bonding distances (Å) and angles (°) for **Complex 2**

| **D–H** | **A** | **d(H···A))/Å** | **d(D···A)/Å** | **∠DHA /°** | **Symmetry** |
| --- | --- | --- | --- | --- | --- |
| O6–H6 | O7 | 1.91 | 2.708(5) | 169.1 | [2–X,–1/2+Y,1–Z] |
| O13–H13 | O14 | 2.01 | 2.807(5) | 167.8 | [2–X,1/2+Y,2–Z] |
| O15–H15A | O7 | 1.78 | 2.626(5) | 173.2 |  |
| O15–H15B | O9 | 1.97 | 2.710(5) | 145.2 |  |
| O16–H16A | O14 | 1.78 | 2.627(5) | 175.5 |  |
| O16–H16B | O2 | 1.90 | 2.700(5) | 158.6 |  |
| O17–H17B | N11 | 1.97 | 2.806(6) | 164.1 |  |
| O18–H18B | N12 | 2.06 | 2.873(5) | 160.6 | [1+X,+Y,1+Z] |
| N3–H3 | O3 | 1.84 | 2.699(5) | 172.6 | [1–X,1/2+Y,1–Z] |
| N5–H5A | N9 | 2.11 | 2.961(6) | 171.1 | [+X,+Y,–1+Z] |
| N8–H8 | O11 | 1.87 | 2.730(5) | 175.6 | [1–X,–1/2+Y,2–Z] |
| N10–H10A | N4 | 2.13 | 2.988(6) | 174.6 | [+X,+Y,1+Z] |

**Supplementary Table 9.** Selected H–bonding distances (Å) and angles (°) for **Complex 3**

| **D–H** | **A** | **d(H···A))/Å** | **d(D···A)/Å** | **∠DHA /°** | **Symmetry** |
| --- | --- | --- | --- | --- | --- |
| O15–H15A | O14 | 1.77 | 2.622(4) | 177.6 |  |
| O15–H15B | O2 | 1.88 | 2.686(4) | 157.7 |  |
| O17–H17A | O7 | 1.77 | 2.616(4) | 171.1 |  |
| O17–H17B | O9 | 1.95 | 2.710(4) | 148.1 |  |
| O6–H6 | O7 | 1.95 | 2.752(4) | 166.7 | [1–X,–1/2+Y,–Z] |
| N5–H5A | N8 | 2.12 | 2.980(5) | 174.6 | [+X,+Y,–1+Z] |
| N4–H4C | O3 | 1.84 | 2.694(4) | 176.0 | [–X,1/2+Y] |
| N10–H10A | N3 | 2.19 | 3.000(5) | 156.5 | [+X,+Y,1+Z] |
| N9–H9 | O10 | 1.87 | 2.728(5) | 175.2 | [–X,–1/2+Y,1–Z] |

**Supplementary Table 10.** Selected H–bonding distances (Å) and angles (°) for **Complex 4**

| **D–H** | **A** | **d(H···A))/Å** | **d(D···A)/Å** | **∠DHA /°** | **Symmetry** |
| --- | --- | --- | --- | --- | --- |
| O9–H9 | O10 | 2.14 | 2.876(13) | 150.1 | 3/2–X,1/2+Y,–Z |
| O3–H3 | N4 | 1.98 | 2.817(11) | 170.0 | +X,+Y,1+Z |
| O1–H1A | O4 | 1.86 | 2.677(14) | 161.6 | 1–X,1–Y,+Z |
| O2–H2 | N4 | 2.08 | 2.934(13) | 179.8 |  |
| N7–H7 | O5 | 1.88 | 2.740(10) | 176.5 | 3/2–X,–1/2+Y |
| N6–H6A | N5 | 2.18 | 3.042(11) | 176.8 | 2–X,1–Y,+Z |

**Supplementary Table 11.** Selected H–bonding distances (Å) and angles (°) for **Complex 5**

| **D–H** | **A** | **d(H···A))/Å** | **d(D···A)/Å** | **∠DHA /°** | **Symmetry** |
| --- | --- | --- | --- | --- | --- |
| N3–H3A | O12 | 2.61 | 3.322(6) | 140.8 | 1–X,+Y,2–Z |
| N5–H5B | O9^2^ | 2.32 | 3.060(5) | 144.4 | 1–X,+Y,1–Z |
| O9–H9D | O2 | 1.79 | 2.624(4) | 167.2 | 3/2–X,1/2+Y,1–Z |
| O9–H9C | O3 | 1.91 | 2.756(5) | 176.9 |  |
| O11–H11C | O7 | 1.86 | 2.686(5) | 163.5 |  |
| O11–H11D | O6 | 1.95 | 2.739(5) | 152.9 | +X,1+Y,+Z |
| O8–H8A | O4 | 1.94 | 2.764(4) | 163.5 | 3/2–X,1/2+Y,2–Z |
| O8–H8B | O3 | 2.17 | 2.918(4) | 146.4 | 3/2–X,1/2+Y,1–Z |
| O12–H12B | O2 | 2.04 | 2.793(4) | 146.0 | 3/2–X,1/2+Y,2–Z |

**Supplementary Table 12.** The H–bonds information of g–motif in complexes **1–4**

|  | **D–H** | **A** | **d(H···A)/Å** | **d(D···A)/Å** | **∠DHA/°** |
| --- | --- | --- | --- | --- | --- |
| **Complex 1** | N5–H5A | N3 | 2.12 | 2.972(9) | 169.8 |
| **Complex 2** | N5–H5A | N9 | 2.11 | 2.961(6) | 171.1 |
|  | N10–H10A | N4 | 2.13 | 2.988(6) | 174.6 |
| **Complex 3** | N5–H5A | N8 | 2.12 | 2.980(5) | 174.6 |
|  | N10–H10A | N3 | 2.19 | 3.000(5) | 156.5 |
| **Complex 4** | N6–H6A | N5 | 2.18 | 3.042(11) | 176.8 |

# Supporting information of Complexes 1–3

**
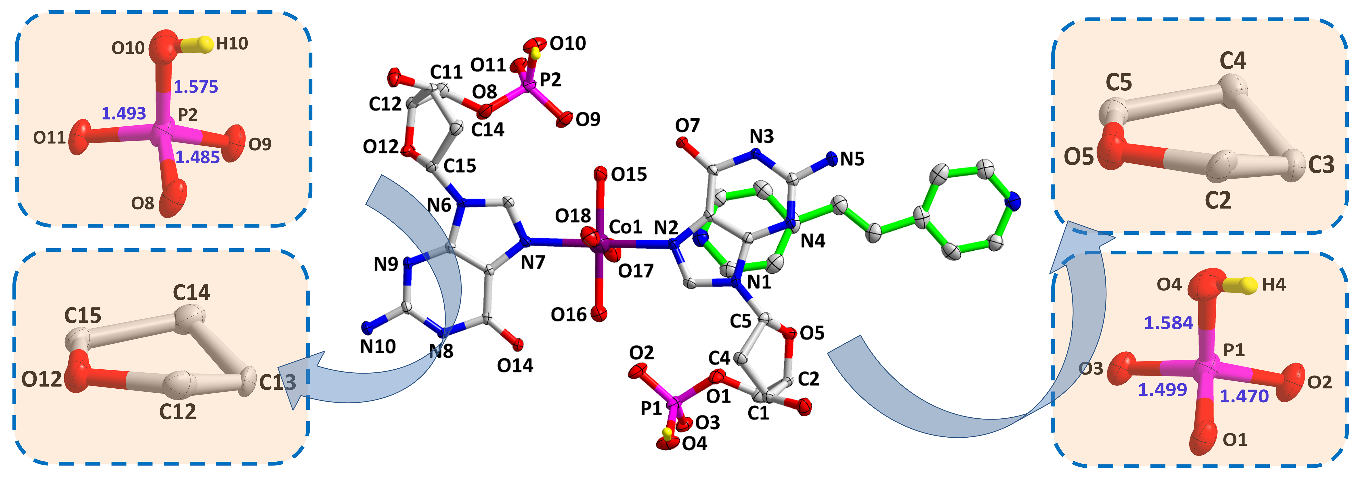
**

**Supplementary Figure 1** Molecular structure of Complex **2**. The hydrogen in Complex **2** are present on O4 and O10 on the phosphate. Both pentose ring conformations are envelope conformation. The bonds of 1,2–*bis*(4–pyridyl)ethane (bpe) are represented by bright green. The uncoordinated water molecules and part of hydrogen atoms are omitted for clarity. (Cobalt: violet, carbon: gray, hydrogen: yellow, oxygen: red, nitrogen: blue and phosphorus: pink)


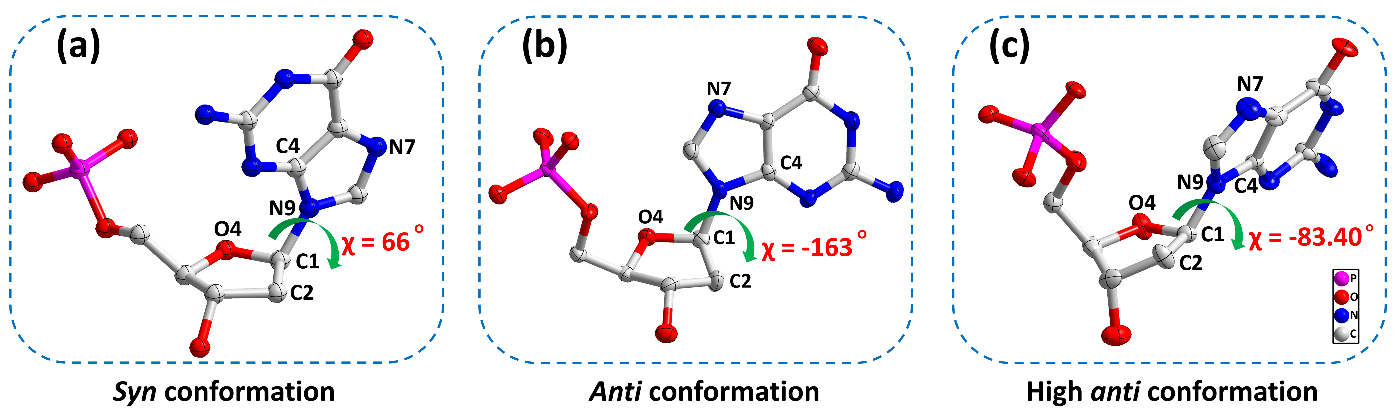


**Supplementary Figure 2 (a)** A guanosine nucleoside with the glycosidic angle angle *χ* set in an *syn* conformation, cif data obtained from the ref. (Kuo et al., 1991) with permission from the American Chemical Society. **(b)** A guanosine nucleoside in an *anti* conformation. **(c)** A guanosine nucleoside in a high *anti* conformation. The sterically preferred ranges for the domains of glycosidic angles are: *Anti*: –120°＞*χ*＞180°; *Syn*: 0＜*χ*＜90°. Values of *χ* in the region of about –90° are often described as “high *anti*”.

**
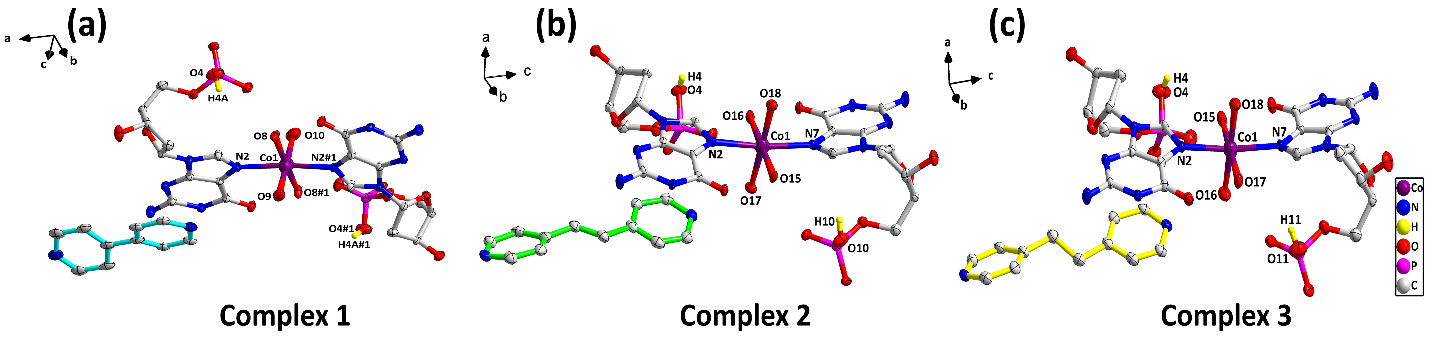
**

**Supplementary Figure 3** Molecular structures of Complexes **1 (a)**, **2 (b) and** **3 (c)**. Cobalt, carbon atoms, hydrogen atoms, oxygen atoms, nitrogen atoms and phosphorus atoms of 2'–Deoxyguanosine–5'–monophosphate disodium salt hydrate (dGMP.2Na) moieties are represented by violet, gray, yellow, red, blue and pink ellipsoids, respectively, whereas the bonds of auxiliary ligands are represented by turquoise (4,4’–bipyridine), bright green (1,2–*bis*(4–pyridyl)ethane), and light yellow (1,2–*bis*(4–pyridyl)ethane). The uncoordinated water molecules and part of hydrogen atoms are omitted for clarity. The protonation of dGMP.2Na is highlight by yellow hydrogen atoms.


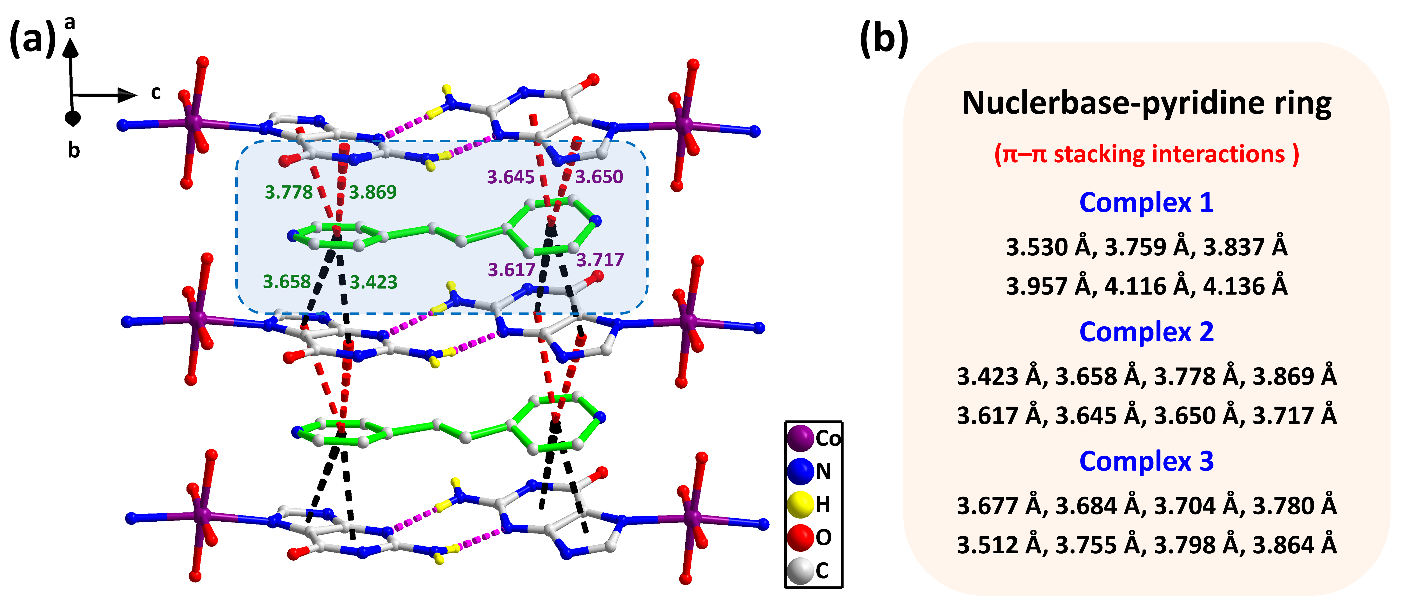


**Supplementary Figure 4** (a) The π–π stacking interactions in Complex **2**. (b) The π–π stacking interactions information of Complexes **1–3**.


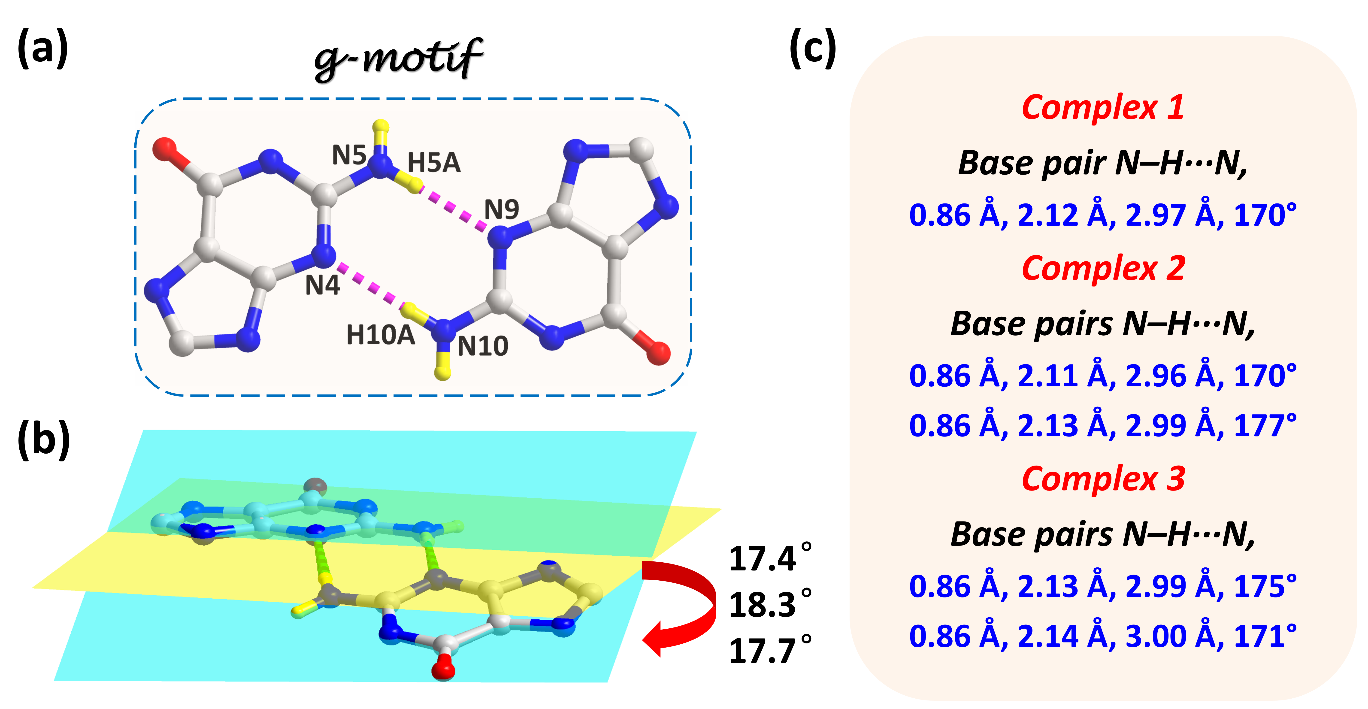


**Supplementary Figure** **5** **(a)** g-motif structure in Complexes **1-3**. **(b)** Dihedral angle between pyridine rings in Complexes **1‒3**. (c) The H-bondings informations of g-motif in Complexes **1‒3**. The uncoordinated water molecules and part of hydrogen atoms are omitted for clarity.

**
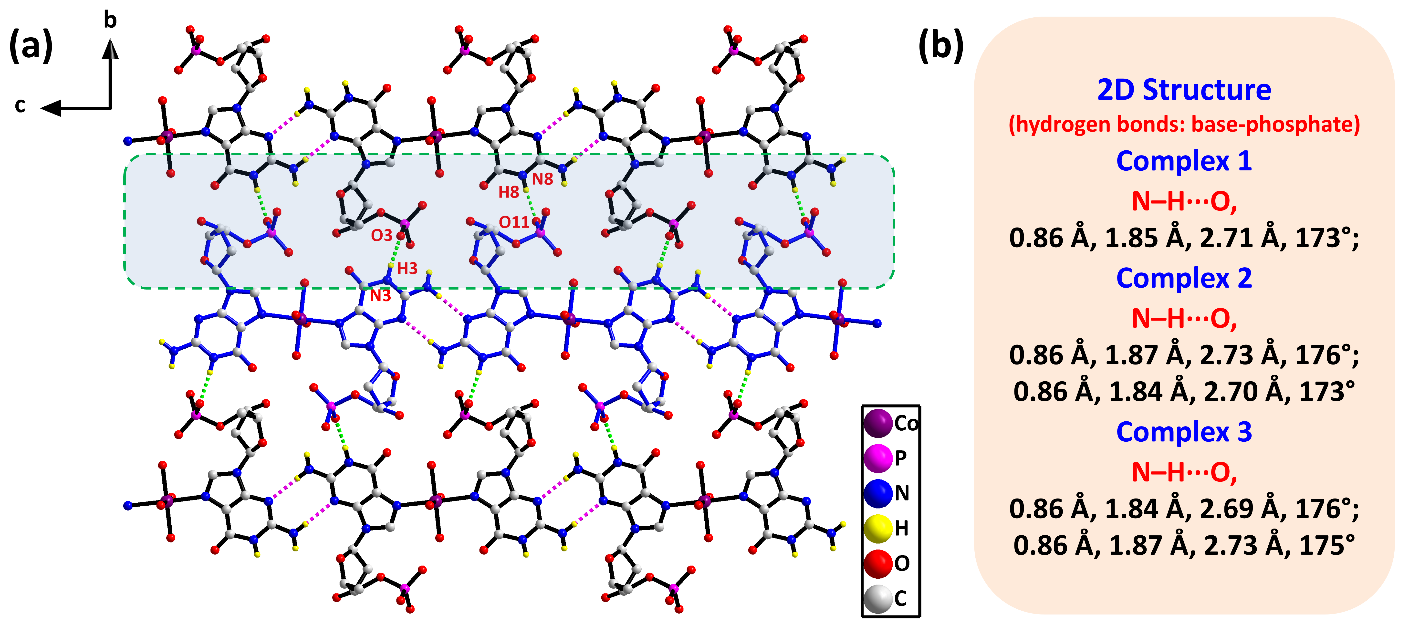
**

**Supplementary Figure 6** **(a)** The 2D supramolecular structure of Complex **2** viewed from *a* axis. **(b)** The related H–bonding informations of Complexes **1‒3**. The pale blue shaded parts are hydrogen bonds of different 1D chain bonds, which are represented by bright green. Free water molecules, auxiliary ligands and part of hydrogen atoms are omitted for clarity. (Cobalt: violet, carbon: gray, hydrogen: yellow, oxygen: red, nitrogen: blue and phosphorus: pink).

**
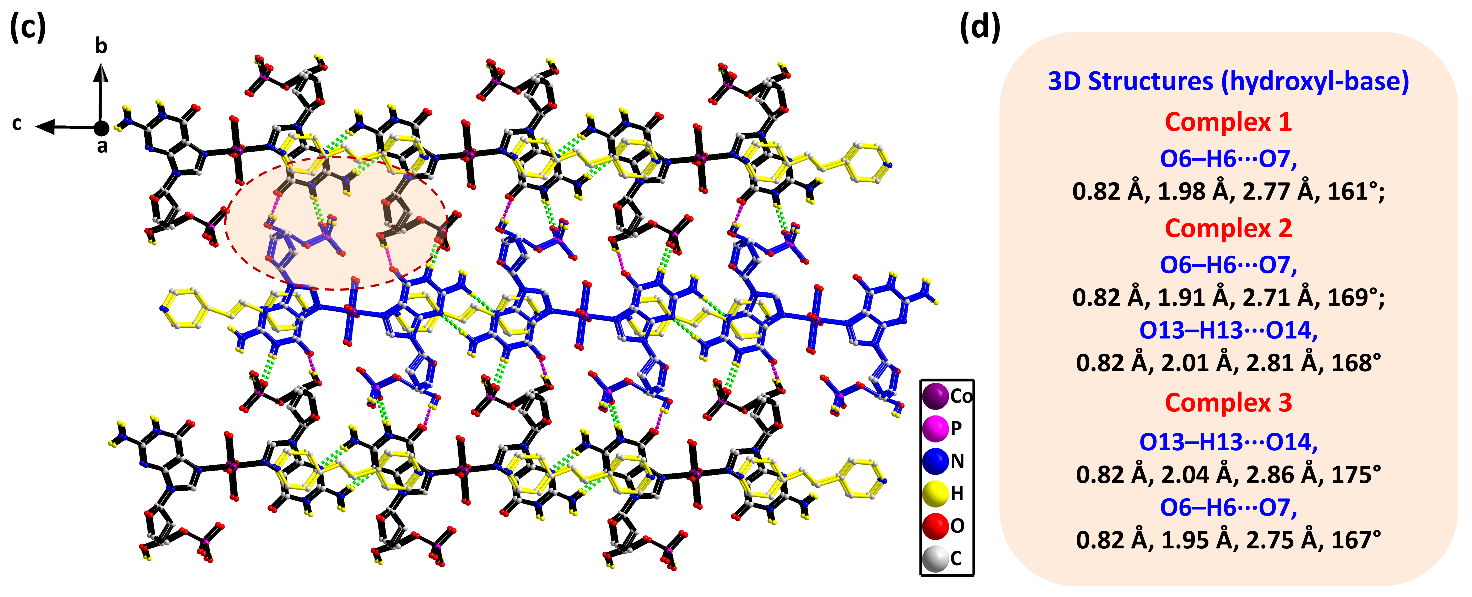
**

**Supplementary Figure 7** **(c)** The 3D supramolecular structure of Complex **2** viewed from *a* axis. **(d)** The related H–bonding informations of Complexes **1‒3**. The light orange shaded parts are hydrogen bonds between different 2D plans, which are represented by pink. The chemical bonds of the auxiliary ligand (bpe) are shown in yellow. Free water molecules and part of hydrogen atoms are omitted for clarity. (Cobalt: violet, carbon: gray, hydrogen: yellow, oxygen: red, nitrogen: blue and phosphorus: pink).

**
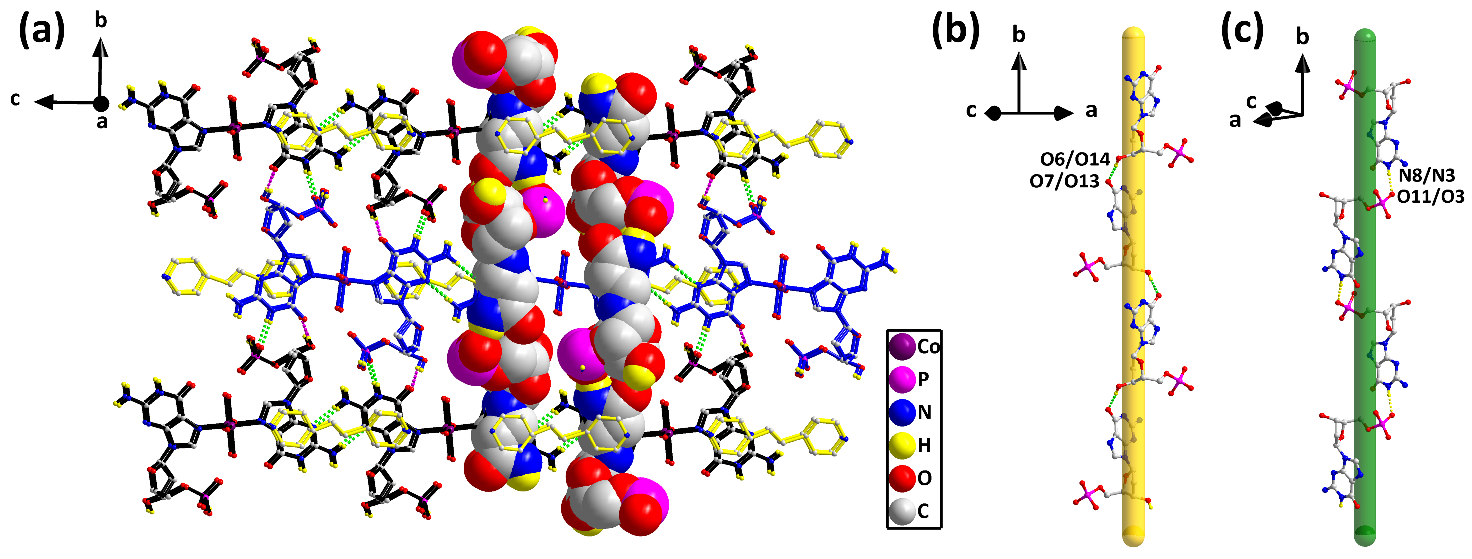
**

**Supplementary Figure 8** **(a)** 3D chiral supramolecular architecture of Complex **2** formed by π–π stacking and inter–layer H–bonding between sheets. Space–filling model used to express the *P* helix existing in *bc* plane. **(b and c)** Schematic presentations of two right–handed helix formed by two different types of hydrogen bonds in *bc* plane (O6–H6···O7, 1.91 Å, 2.71 Å, 169°; O13–H13···O14, 2.01 Å, 2.81 Å, 168° and; N3–H3···O3, 1.84 Å, 2.70 Å, 173°; N8–H8···O11, 1.87 Å, 2.73 Å, 176°; bright green and yellow dotted lines). Only atoms of nucleotide (dGMP.2Na) are shown. (Cobalt: violet, carbon: gray, hydrogen: yellow, oxygen: red, nitrogen: blue and phosphorus: pink).

# Supporting information of Complex 4

**
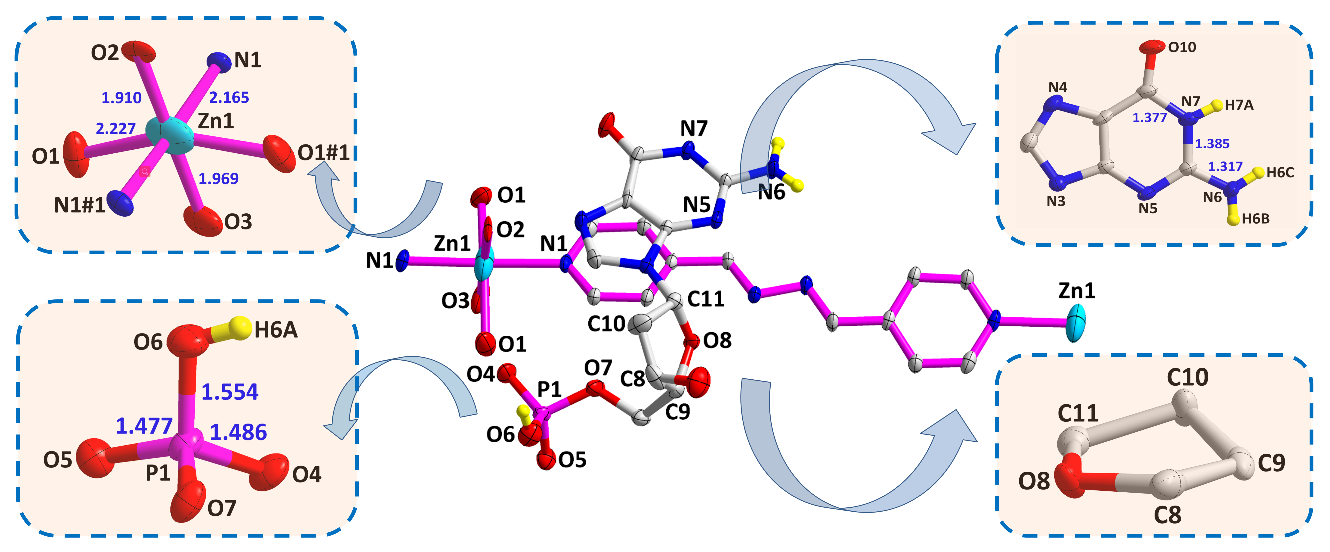
**

**Supplementary Figure 9** Molecular structure of Complex **4**. The hydrogen in Complex **4** is present on O6 on the phosphate. The pentose ring conformation is envelope conformation. The bonds of 1,4–*bis*(4–pyridyl)–2,3–diaza–1,3–butadiene (bpda) are represented by pink. The uncoordinated water molecules and part of hydrogen atoms are omitted for clarity. (zinc: sky blue, carbon: gray, hydrogen: yellow, oxygen: red, nitrogen: blue and phosphorus: pink)


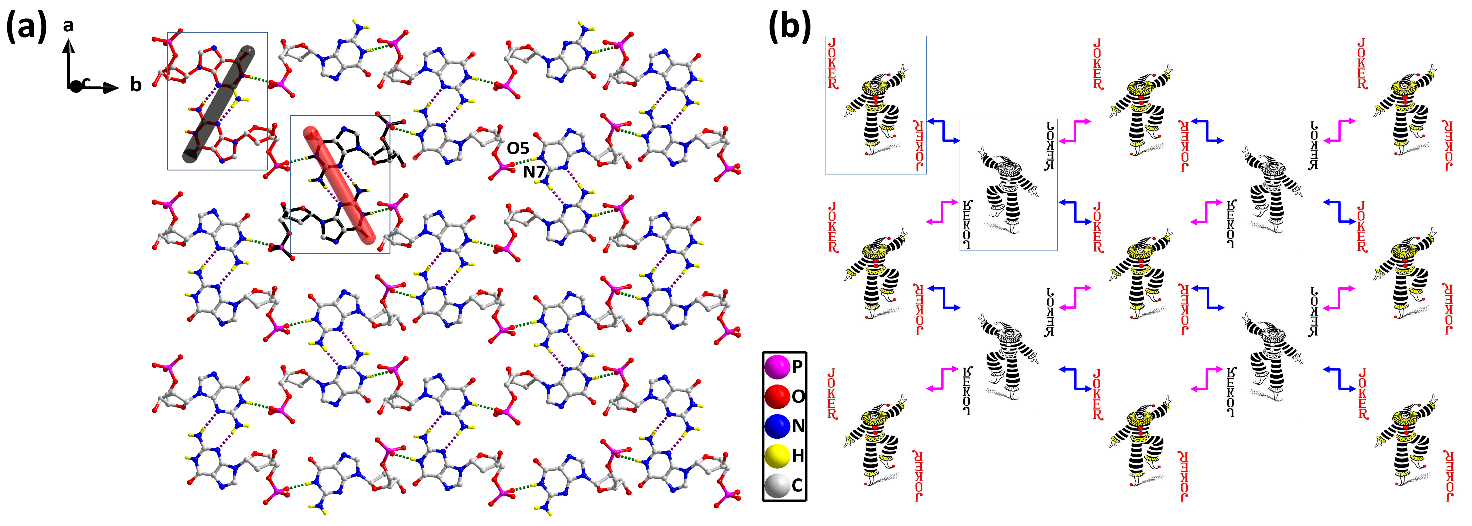


**Supplementary Figure 10** (a) The 2D supramolecular structure of Complex **4** viewed from *c* axis. (b) The cartoon picture of the 2D supramolecular structure of **4**. Free water molecules, auxiliary ligands and part of hydrogen atoms are omitted for clarity. (Carbon: gray, Hydrogen: yellow, Oxygen: red, Nitrogen: blue and Phosphorus: pink).

**
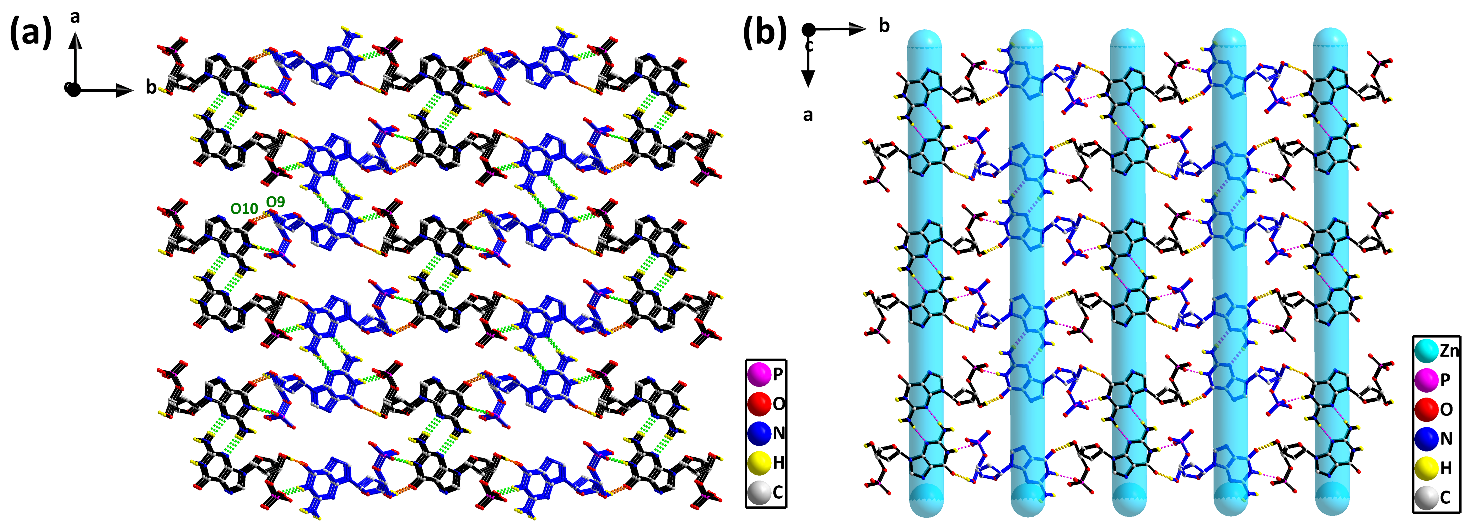
**

**Supplementary Figure 11** (a) The 3D supramolecular structure of Complex **4** viewed from *c* axis. (b) The electrostatic interaction between the 3D supramolecular structure and 1D cationic chains viewed from *c* axis. Free water molecules, auxiliary ligands and part of hydrogen atoms are omitted for clarity. (Zinc: turquoise, Carbon: gray, Hydrogen: yellow, Oxygen: red, Nitrogen: blue and Phosphorus: pink).

# Supporting information of Complex 5

Abundant hydrogen bonds are an important reason for chiral molecules of Complex **5** deliver to the 3D supramolecular architecture. The first is intramolecular hydrogen bonds, which were formed by coordinated water molecules with phosphate oxygen atoms and base oxygen atoms in the asymmetric unit (O9–H9C···O3, 1.91 Å, 2.76 Å, 177°; O11–H11C···O7, 1.86 Å, 2.69 Å, 164°) (Supplementary Figure **12a**). Then the separate [Mn(dGMP)(H_2_O)_5_]·3H_2_O molecules together into a 1D right–handed helical chains along *b* axis through the intermolecular hydrogen bond among coordination water molecules and donors of the phosphate oxygen atom and hydroxy of pentose (O9–H9D···O2, 1.79 Å, 2.62 Å,167°; O11–H11D···O6, 1.95 Å, 2.74 Å, 153°), with a pitch of 11.318 Å (Supplementary Figure **12e**). Followed by the 1D helical chains are linked *via* π–π stacking interactions among purine and anther purine of the dGMP^2–^ ligands (3.425‒3.926 Å) into a 2D hydrogen bonding layer (Supplementary Figure **12c**). Here, the π–π stacking interaction can not only stabilize the flexible conformation of nucleotide ligands, but also helpful for the chiral transmission. At last, these 2D sheets can be further assembled into a 3D supramolecular framework based on hydrogen bonds formed by the coordination water with phosphate oxygen atoms (O12–H12B···O2, 2.04 Å, 2.80 Å, 146°) (Supplementary Figure **12c**). Clearly, the complex **5** from 0D structure to 3D supramolecular structure can be simplified to the topological structure as shown in Supplementary Figure **12b**.


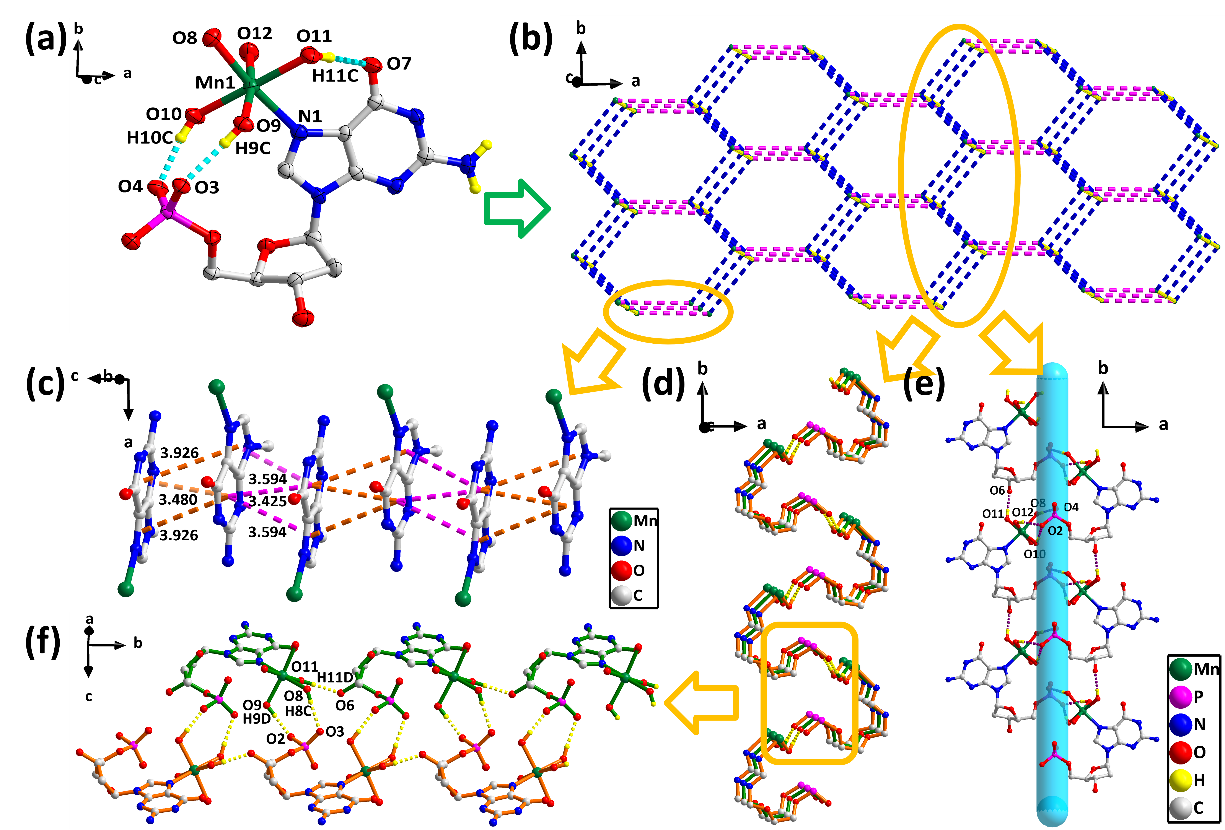


**Supplementary Figure 12** **(a)** Molecular structure of Complex **5**, the uncoordinated water molecules and part of hydrogen atoms are omitted. **(b)** The topological graph of **5**. **(c)** π–π interaction between 3D Planes. **(d)** The alternative neighbor right–handed helical chains connected by H–bonding in the crystal lattice of complex **5**. **(e)** 1D right–handed helical chain of **5**. **(f)** Hydrogen bonding between neighbor right–handed helical chains. (Manganese: green, carbon: gray, hydrogen: yellow, oxygen: red, nitrogen: blue and phosphorus: pink).


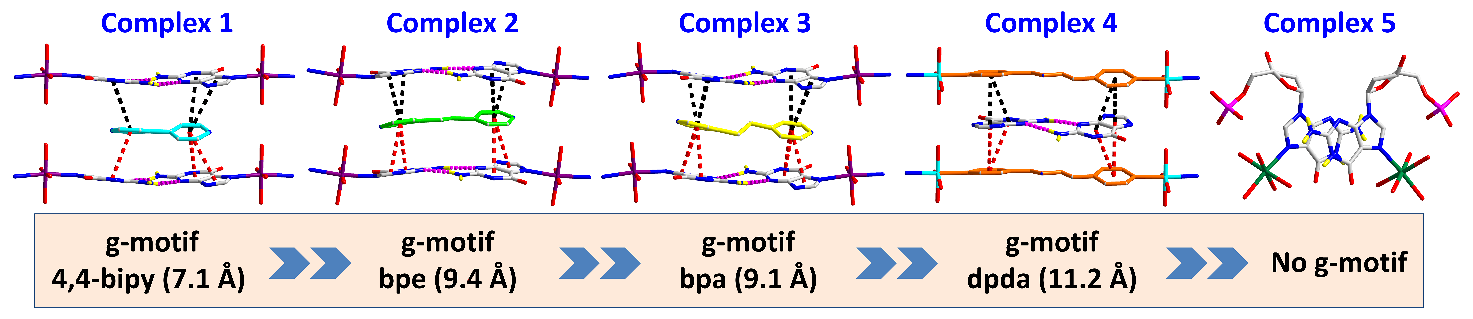


**Supplementary Figure 13** Summary of crystal structures presenting g–motif and π–π stacking interaction in Complexes **1–5**.

# Supporting information of nucleotide ligand


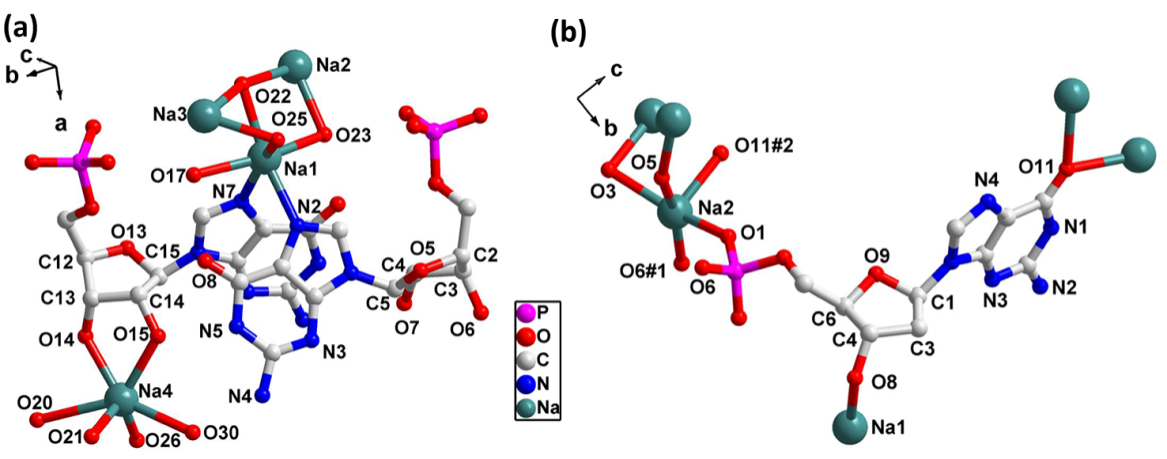


**Supplementary Figure 14.** View of the coordination environment of GMP.2Na **(a)** and dGMP.2Na **(b)**, cif data obtained from the ref. (Dračínský et al., 2014; Young et al., 1974 ) with permission.

In order to investigate the chirality of dGMP.2Na ligand and its complexes in more detail at solid state, we introduce GMP.2Na ligand as a comparison (Supplementary Figure **14**). The circular dichroism spectrums of the two chiral ligands, GMP.2Na and dGMP.2Na, is very similar in aqueous solution (Supplementary Figure **15**). Therefore, dGMP.2Na, with one less hydroxyl group of the sugar ring does not affect the chiral behavior in the solution state. From the solid CD spectra of this two ligands, we found that GMP.2Na has two characteristic peaks at 226 nm (+) and 270 nm (‒); dGMP.2Na has four characteristic peaks of 232 nm (+), 258 nm (‒), 283 nm(+) and 297 nm(‒) (Supplementary Figure **16** and Supplementary Table **14**). Relevant literatures indicate that non–covalent interactions, such as hydrogen bonds and π–π stacking interactions, will sensitively affect the solid CD spectrum of the compound (Sponer et al., 2001; Donald and Alexander, 1970; Qiu et al., 2018). Therefore, we analyzed the non–covalent interaction between GMP.2Na and dGMP.2Na in detail through the crystal structures (Supplementary Figure **17** and Supplementary Figure **18**). In both GMP.2Na and dGMP.2Na ligands, there were no π–π stacking effect, but the intra– and intermolecular hydrogen bonds formed by GMP.2Na are stronger and more than dGMP.2Na, which reduces the energy of the GMP.2Na system and causes absorption peak to shift to the long–wave direction (258→270 nm) (Supplementary Figure **16**). Furthermore, *P* is 173.2° and 167.98° for GMP.2Na molecular from the crystal structure, respectively, so that the conformation of the sugar is C(2’)–*endo*, which both prefer twist conformation (T). The crystal structure shows that the twist conformation of GMP.2Na can be induced and stabilized by the coordination of Na^+^, as well as the hydrogen bonds formed by coordination waters, phosphate groups, and hydroxyl groups of the sugar rings (O6–H9···O10, 1.70 Å, 2.69 Å, 174°; O18–H21···O6, 2.07 Å, 2.95 Å, 154°; O21–H27···O6, 2.20 Å, 3.07 Å, 149°; O26–H34···O7 , 1.68 Å, 2.80 Å, 162°) (Supplementary Table **15**); the envelope conformation of dGMP.2Na also can be controlled by the coordination of Na^+^ and the hydrogen bonds between the coordination waters and the hydroxyl group of the sugar ring (O8–H20···O2, 1.93 Å, 2.74 Å, 165°) (Supplementary Table **15**). Therefore, we have reason to suspect that the two more characteristic peaks at 283 nm (+) and 297 nm (‒) in the dGMP.2Na solid CD spectrum is probably caused by the different conformation of the pentose rings. Reviewing the crystal structure and solid CD spectra of pyrimidine monophosphate nucleotides, CMP.2Na and dCMP.2Na also exhibit twist conformation (T) and envelope conformation (E), respectively. As we expected, dCMP.2Na also has an extra CD signal.


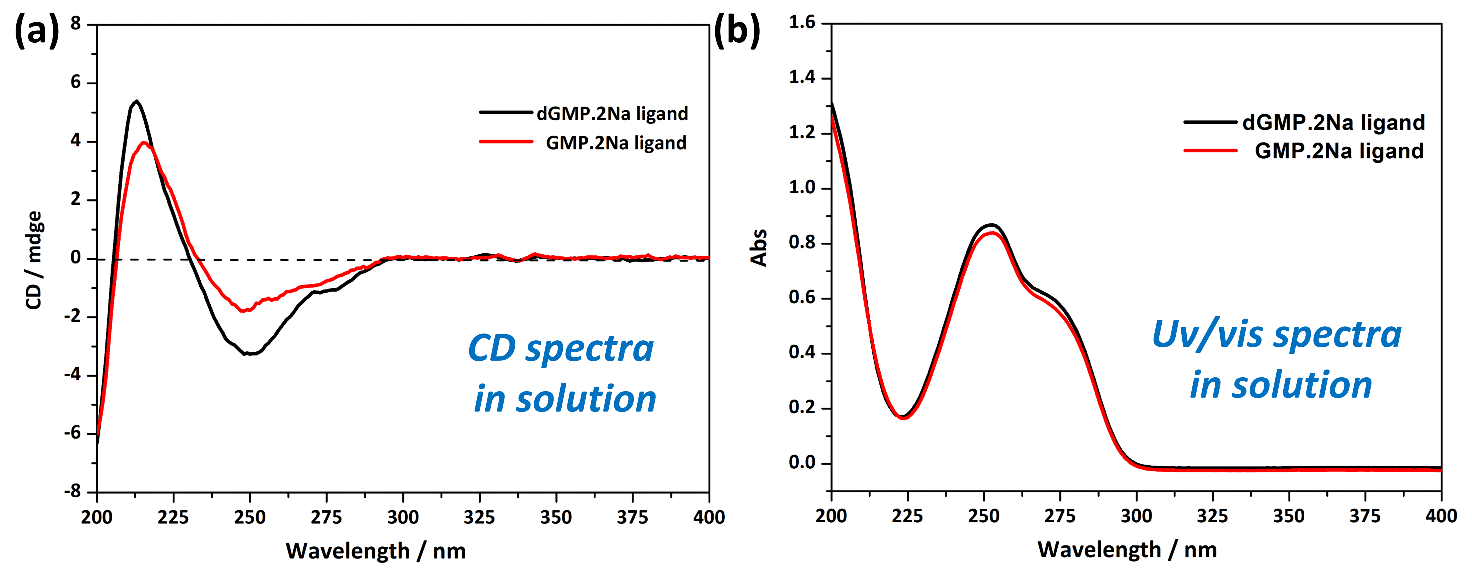


**Supplementary Figure 15 (a)** The CD spectra of the solution of GMP.2Na and dGMP.2Na. The sample is same as the UV/*vis* absorption spectra measurement. **(b)** UV/*vis* absorption spectra of GMP.2Na and dGMP.2Na in aqueous solution at room temperature, the solution spectra were obtained by measuring 3.25×10^–5^ mol**·**L^–1^ solution in a 1 cm cell.

**Supplementary Table 13.** H–bonding distances (Å) and angles (°) for GMP.2Na and dGMP.2Na.

| **D–H** | **d(H···A)** | **∠DHA** | **d(D···A)** | **A** | **Symmetry** |
| --- | --- | --- | --- | --- | --- |
| **GMP.2Na**  N5–H1 (n)  N10–H11 (n)  O6–H9 (h)  O19–H23 (cw)  O19–H24 (cw)  O20–H26 (cw)  O21–H28 (cw)  O22–H29 (cw)  O23–H30 (cw)  O18–H21 (cw)  O21–H27 (cw)  O26–H34 (cw)  N4–H3 (n)  O24–H31 (fw)  O24–H32 (fw)  O29–H36 (fw)  **dGMP.2Na**  N1–H1 (n)  N2–H12 (n)  O2–H2 (cw)  O2–H3 (cw)  O3–H4 (cw)  O3–H5 (cw)  O5–H8 (cw)  O4–H6 (cw)  O8–H20 (cw) | 1.51  1.83  1.70  1.85  1.99  2.02  1.62  2.14  1.78  2.07  2.20  1.68  1.78  1.70  2.02  1.60  1.87  2.09  2.11  1.96  2.12  2.12  2.09  2.05  1.93 | 163  158  174  145  145  156  159  163  149  154  149  162  153  170  145  149  169  170  149  172  174  153  166  168  165 | 2.69  2.75  2.69  2.74  2.84  2.79  2.69  3.01  2.75  2.95  3.07  2.80  2.95  2.84  2.98  2.73  2.76  2.96  2.73  2.77  2.86  2.76  2.78  2.78  2.74 | O3 (p )  O11 (p)  O10 (p)  O10 (p)  O1 (p)  O2 (p)  O1 (p)  O9 (p)  O16 (n)  O6 (h)  O6 (h)  O7 (h)  O29 (fw)  O9 (p)  O13 (o)  O26(cw)  O1 (p)  O10 (p)  O7 (p)  O6 (p)  O6 (p)  O7 (p)  O7 (p)  N4 (n)  O2 (cw) | [*x* + 2, *y* + 1/2, –*z* + 3/2]  [*x* + 1, *y* + 1/2, –*z* + 3/2]  [*x* + 1/2, *y* – 1/2, *z* + 1]  [*x* – 1/2, –*y* + 1/2, *z* + 1]  [*x* – 1, *y* – 1/2, –*z* + 3/2]  [*x* – 1/2, –*y* + 1/2, *z* + 2]  [*x* – 1, *y* , *z*]  [*x* – 1, *y* , *z*]  [*x* – 1, *y* , *z*]  [–*x* + 3/2, *y* + 1, *z* + 1/2]  [*x* – 1/2, –*y* + 1/2, *z* + 1]  [*x* + 1/2, *y* + 1, –*z* + 3/2]  [*x*, *y*, *z* – 1]  [–*x*, *y* + 1/2, –*z* + 1]  [–*x*, *y* + 1/2, –*z* + 1]  [–*x*, *y* – 1/2, –*z* + 1]  [*x*, *y*, *z* + 1]  [1 – *x*, *y* – 1/2, –*z*]  [–*x*, *y* – 1/2, –*z*]  [1 – *x*, *y* – 1/2, –*z*]  [*x* + 1, *y*, *z*] |

(cw) refers to coordinated water. (fw) refers to free water. (p) refers to phosphate group.

(n) refers to nucleobase. (h) refers to ribose hydroxyl. (o) refers to ribose oxygen


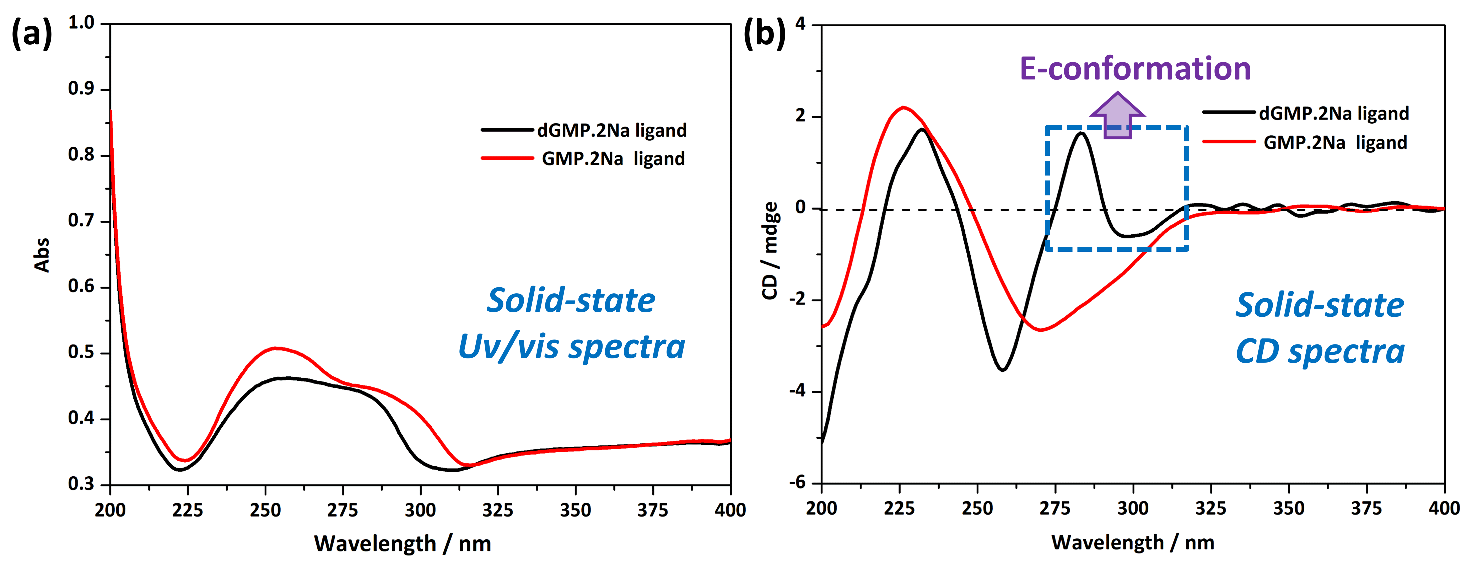


**Supplementary Figure 16 (a)** UV/*vis* absorption spectra of GMP.2Na and dGMP.2Na in solid–state at room temperature. **(b)** The solid–state CD spectra of GMP.2Na and dGMP.2Na (KBr : [sample] = 200 : 1).

**Supplementary Table 14.** The relative CD absorption peaks, π–π stacking interaction, H–bonding and pentose conformation of GMP.2Na and dGMP.2Na.

| **Peak (nm)** | **λ_1_** | **λ_2_** | **λ_3_** | **π–π**  **stacking**  **interaction** | **H–bonding** | **Pentose**  **Conformation** |
| --- | --- | --- | --- | --- | --- | --- |
| **GMP.2Na** | **226 (+)** | **270 (–)** |  | **No** | **Complicated**  **H–bonding** | **T** |
| **dGMP.2Na** | **232 (+)** | **258 (–)** | **283 (+)**  **298 (–)** | **No** | **H–bonding** | **E** |


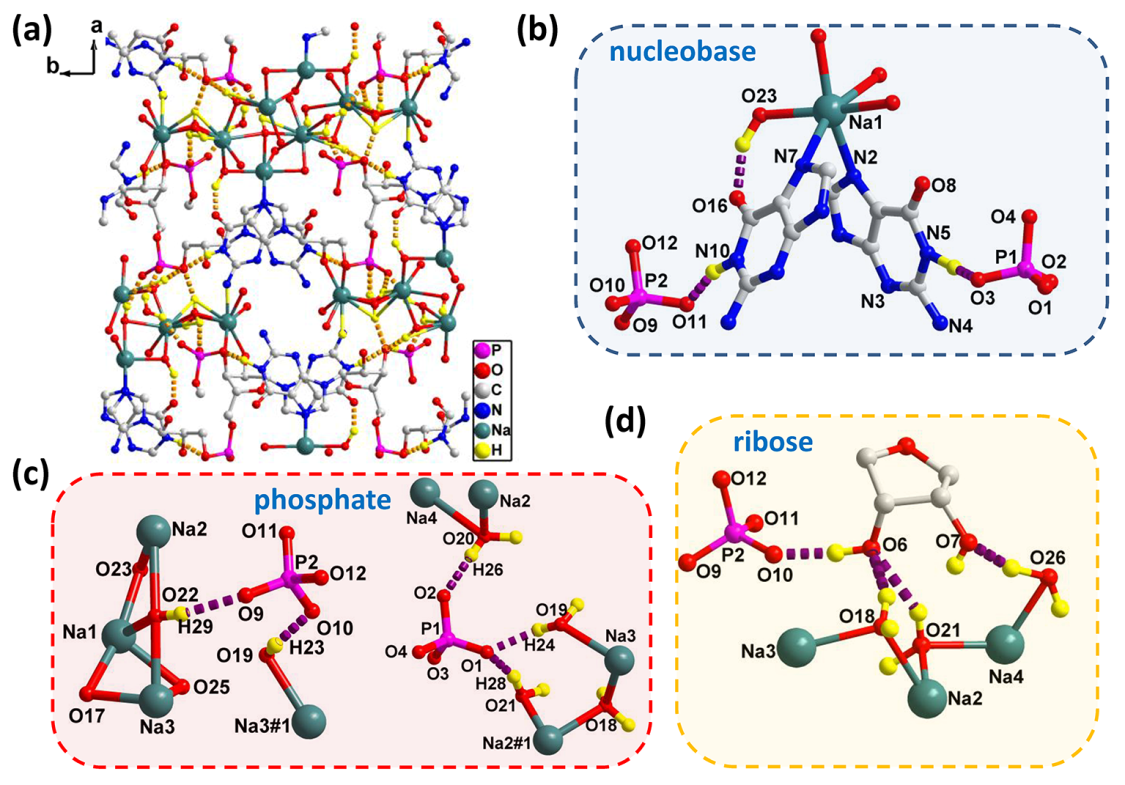


**Supplementary Figure 17** (a) The stacking structure of GMP.2Na along the *c* axis. (b) H–bonding among nucleobase, phosphate and coordinated water (O–H···O, 1.78 Å, 2.75 Å, 149°; N–H···O, 1.51–1.83 Å, 2.69–2.75 Å, 158–163°). (c) H–bonding between phosphate and coordinated water (O–H···O, 1.62–2.14 Å, 2.69–3.01 Å, 145–163°). (d) H–bonding among hydroxyl, phosphate and coordinated water (O–H···O, 1.68–2.20 Å, 2.69–3.07 Å, 149–174°).


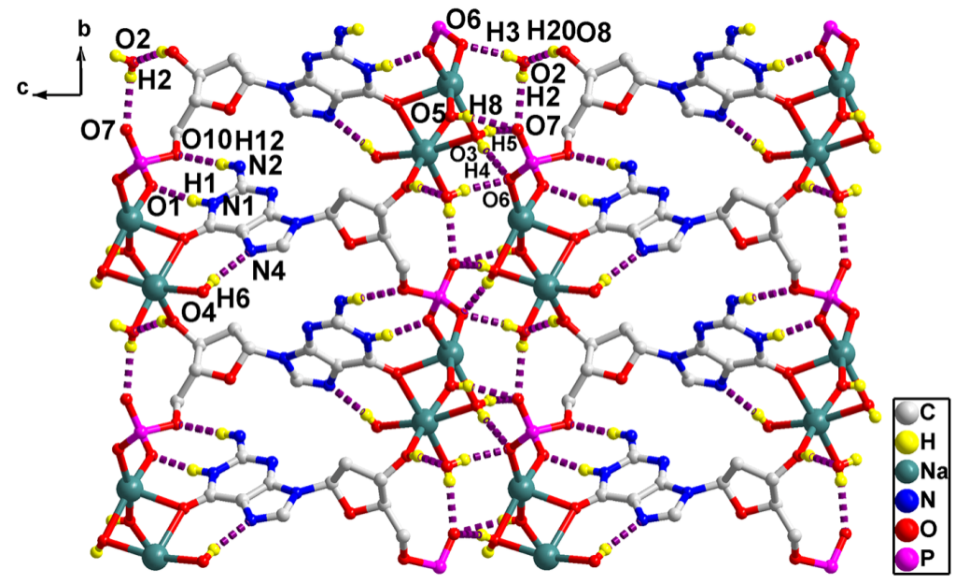


**Supplementary Figure 18** (a) The stacking structure of dGMP.2Na along *a* axis. (b) H–bonding among nucleobase, phosphate, hydroxyl and coordinated water (O–H···O, 1.93–2.12 Å, 2.73–2.86 Å, 149–174°; N–H···O, 1.87–2.09 Å, 2.76–2.96 Å, 169–170°).

# The UV/*vis* absorption spectrums of the complexes


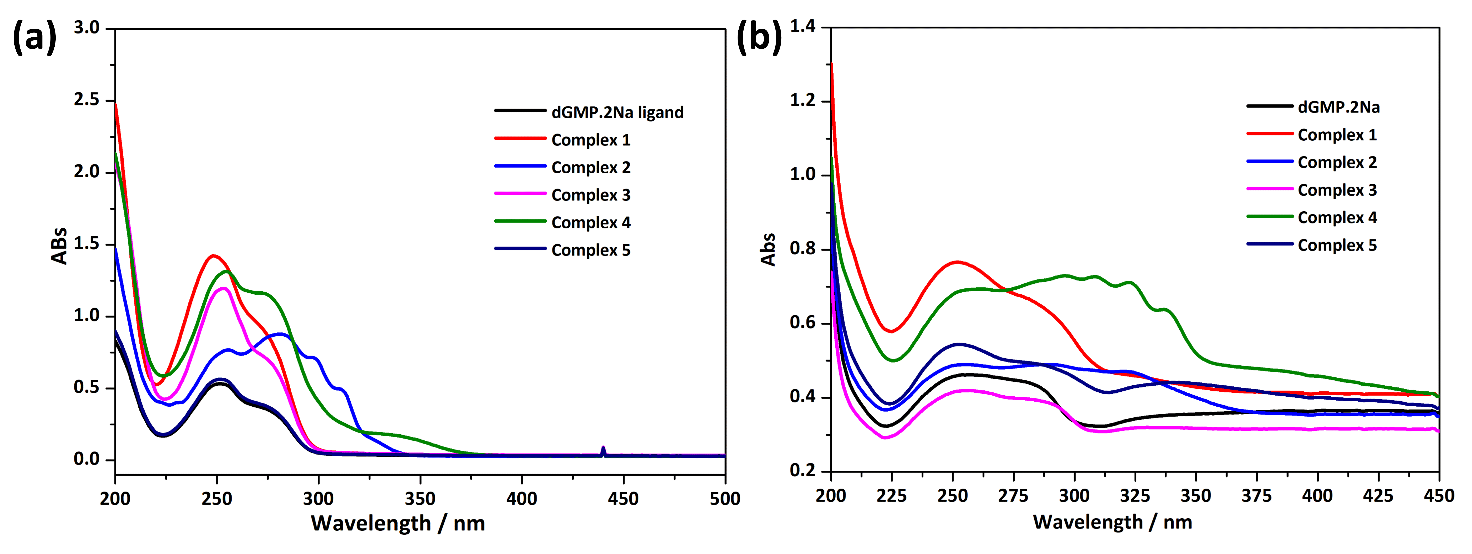


**Supplementary Figure 19 (a)** The liquid–state UV/*vis* absorption spectrum of dGMP.2Na and its complexes in aqueous solution at room temperature, the solution spectra were obtained by measuring 3.25×10^–5^ mol/L solution in a 1 cm cell. **(b)** The solid–state UV–*vis* spectrum of complexes **1–5** at room temperature. (KBr : [sample] = 200 : 1).

**Supplementary Table 15.** The relative CD absorption peaks, π–π stacking interaction, H–bonding and pentose conformation of dGMP.2Na and complex 1–5.

| **Peak (nm)** | ***λ_1_*** | ***λ_2_*** | ***λ_3_*** | ***λ_4_*** | **π–π stacking** | **H–bonding** |
| --- | --- | --- | --- | --- | --- | --- |
| **dGMP.2Na** | 231 (+) | 258 (–) | 283 (+)  300 (–) |  | No | No base–pair |
| **Complex 1** | 235(+) | 265(–) |  |  | Yes | base–pair |
| **Complex 2** | 224–285 (+) | |  | 318(–) | Yes | base–pair |
| **Complex 3** | 221–284 (+) | |  | 312(–) | Yes | base–pair |
| **Complex 4** | 235(+) | 263(–) |  |  | Yes | base–pair |
| **Complex 5** | 222(+) | 264 (–) | 281(+)  301 (–) |  | Yes | No base–pair |

**
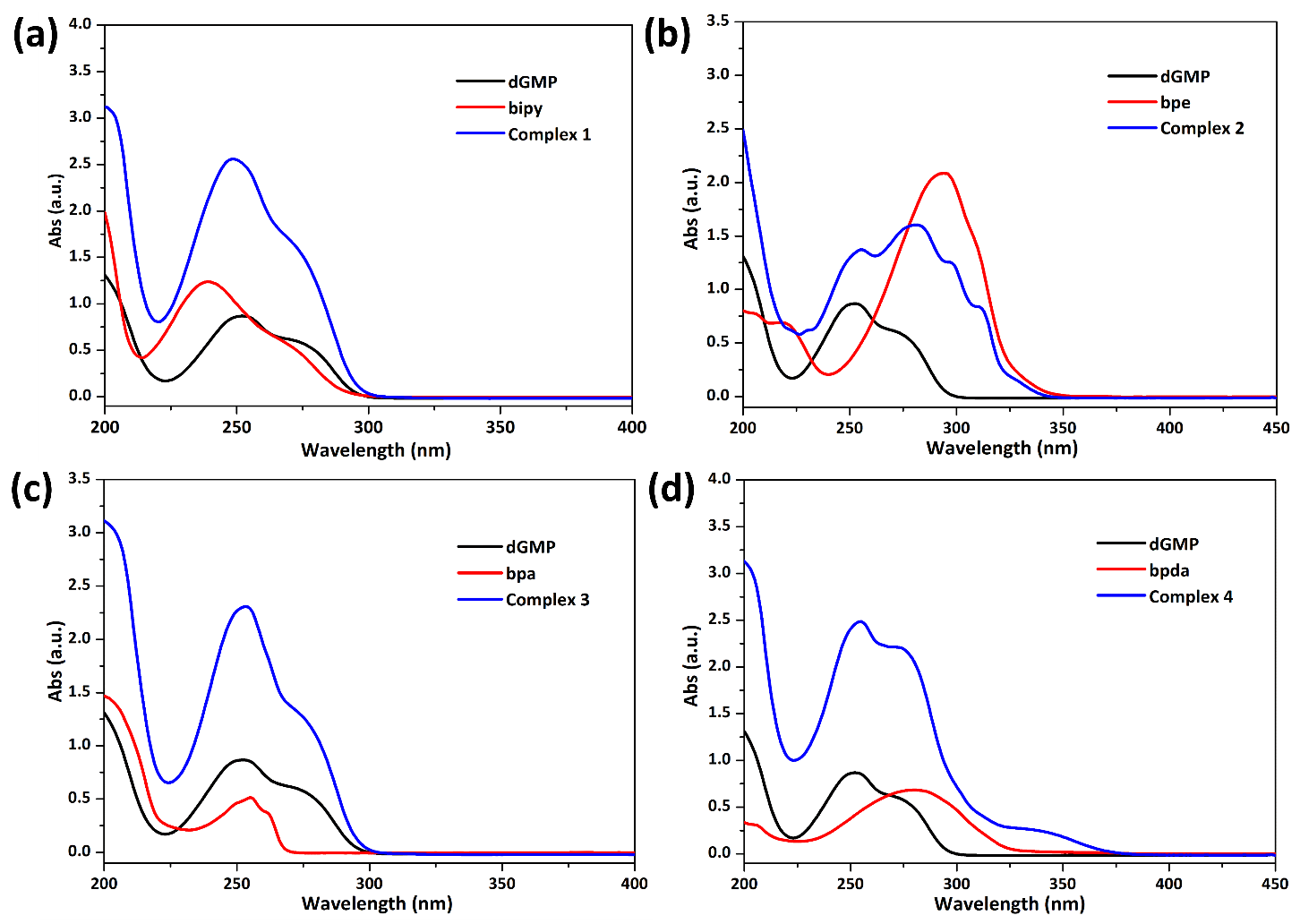
**

**Supplementary Figure 20** UV–*vis* absorption spectra of **(a)** dGMP, bipy and complex **1**; **(b)** dGMP, bpe and complex **2**; **(c)** dGMP, bpa and complex **3**; **(d)** dGMP, bpda and complex **4**; The spectrum are obtained by measuring 5 ×10^–5^ mol/L solution in a 1 cm cell.


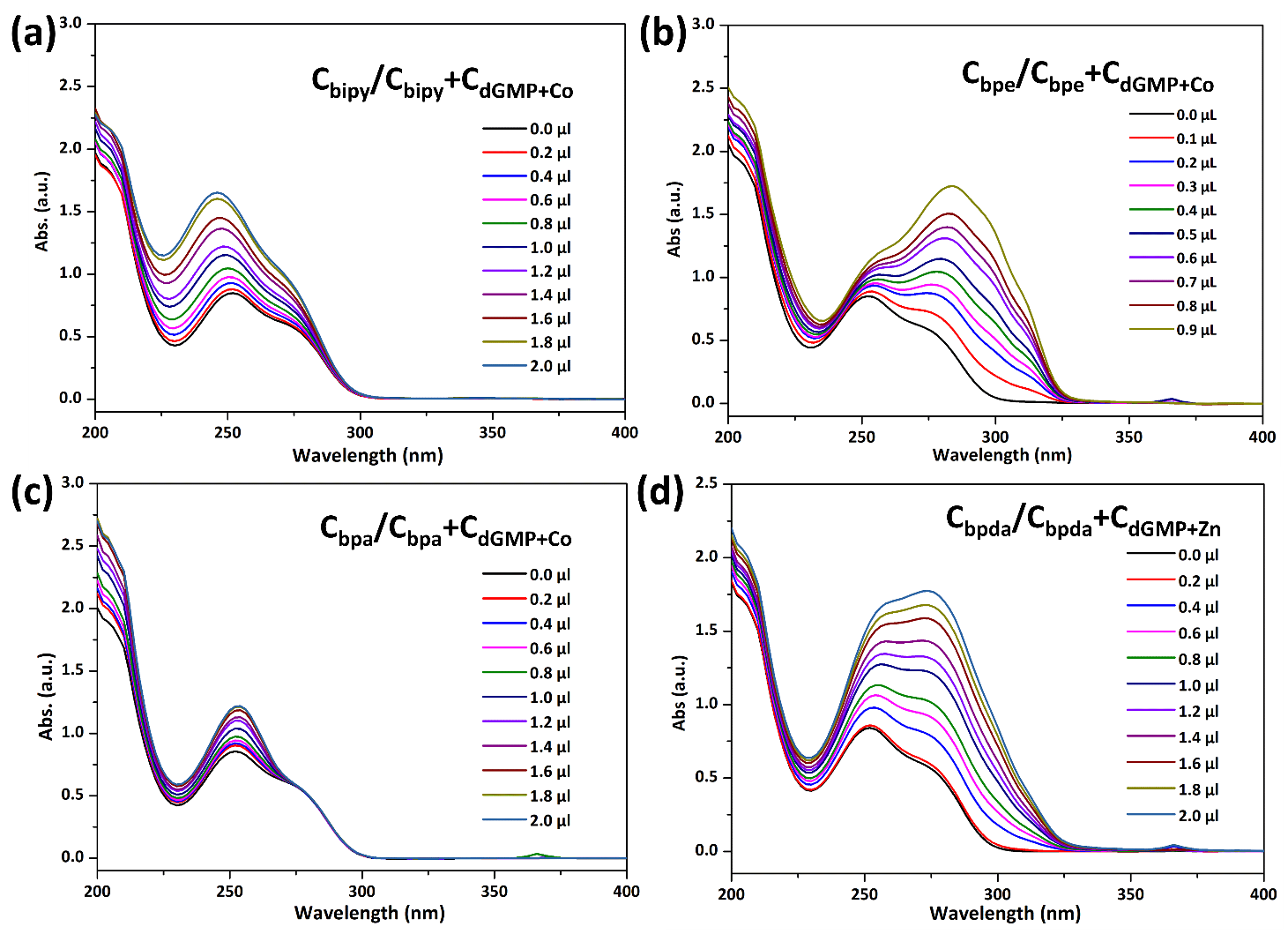


**Supplementary Figure 21** Electronic spectra of dGMP–Co/ dGMP–Zn (1:1) upon addition of bridging ligands (4,4–bipy, bpe, bpa and bpda). The total concentration of dGMP–Metal and bridging ligands is held fixed (5 × 10^−5^ M) varying the ratio of the components C*_bridging ligand_*/C*_dGMP–M_*. **(a)** C*_4,4–bipy_*/C*_4,4–bipy_*+C*_dGMP+Co_* **(b)** C*_bpe_*/C*_bpe_* +C*_dGMP+Co_* (c) C*_bpa_*/C*_bpa_* +C*_dGMP+Co_* (d) C*_bpda_*/C*_bpda_* +C*_dGMP+Zn_*.

# The ESI–MS Spectrum


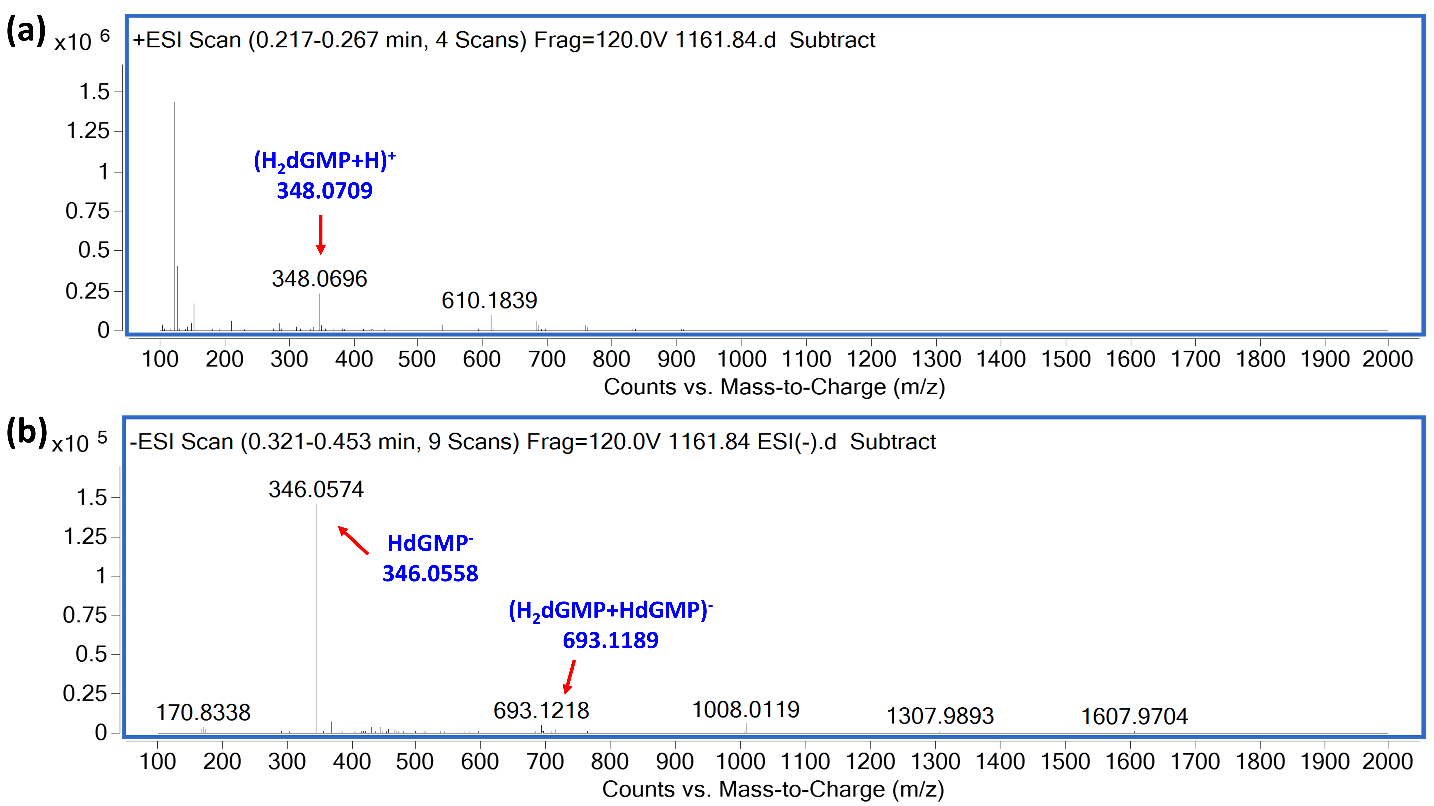


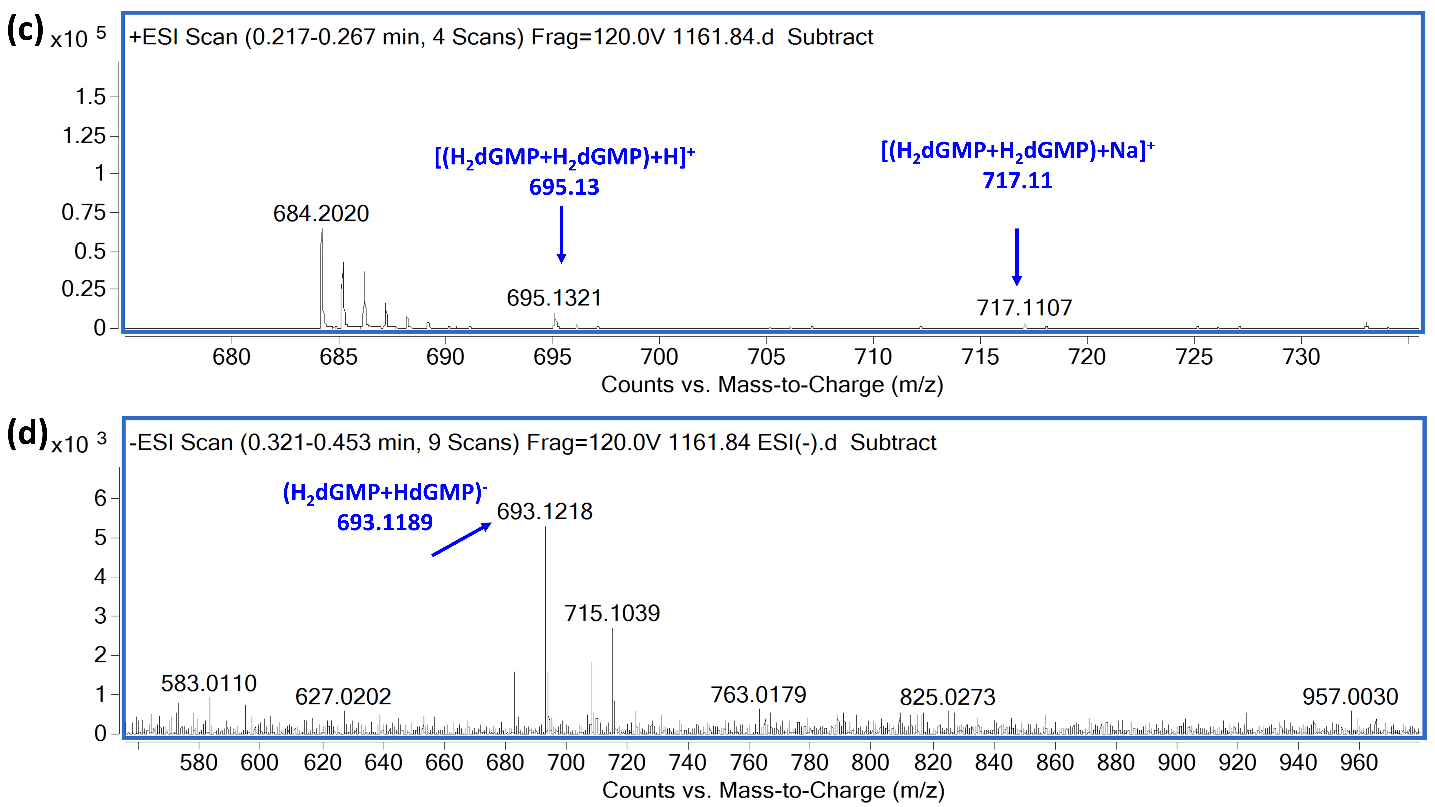


**Supplementary Figure 22** ESI–MS spectrum of Complex **4**.

# ^1^H NMR Spectroscopy


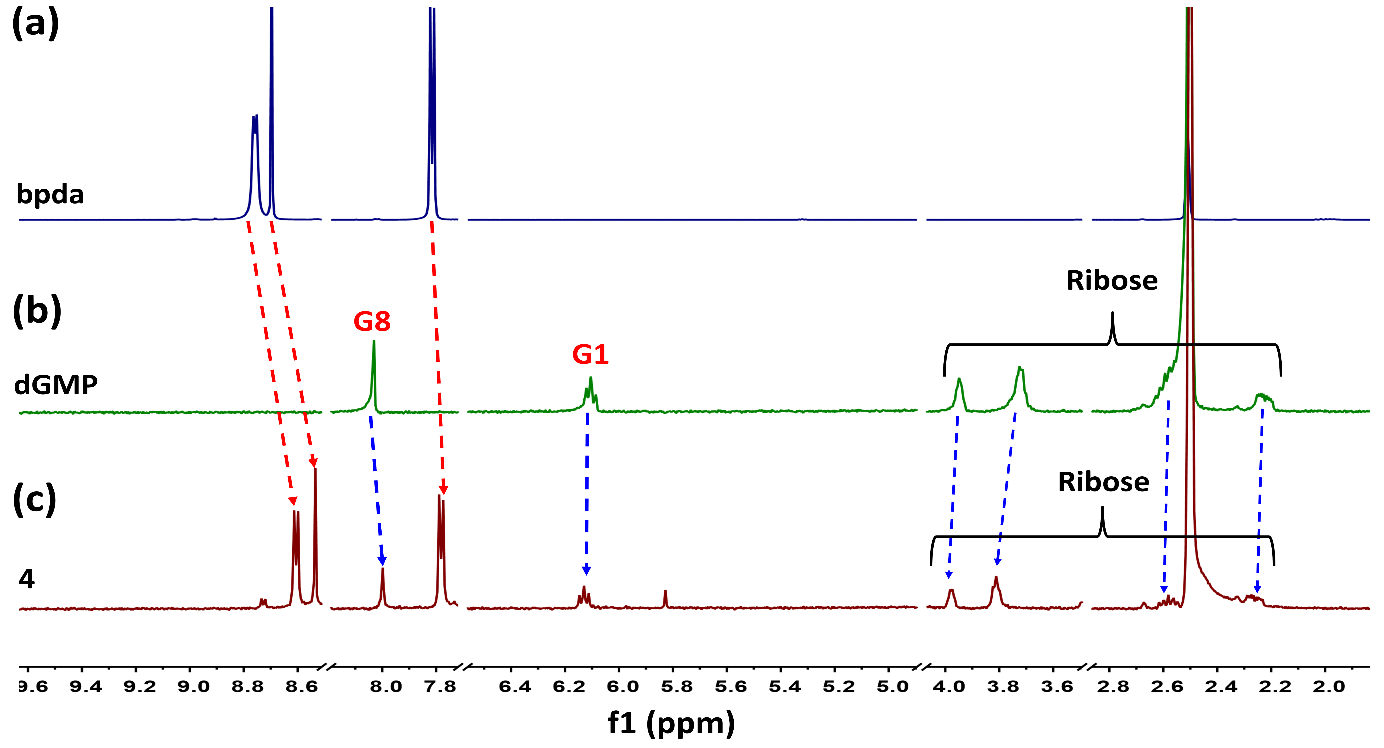


**Supplementary Figure 23** (a), (b) and (c) ^1^H NMR spectrum of bpda in solution (D_2_O : DMSO–*d_6_* = 2:1) at room temperature, respectively. The guanine H1 and H8 signals shift downfield and upfield, respectively, indicating formation of the g–motif structure. The ribose protons signal around 2.2–4.0 ppm shift only slightly, indicating the absence of major changes in the environment. The three aromatic signals derived from bpda (8.5, 8.6 and 7.8 ppm) also shifted significantly upfield, presumably owing to slow equilibration in the host–guest complex.


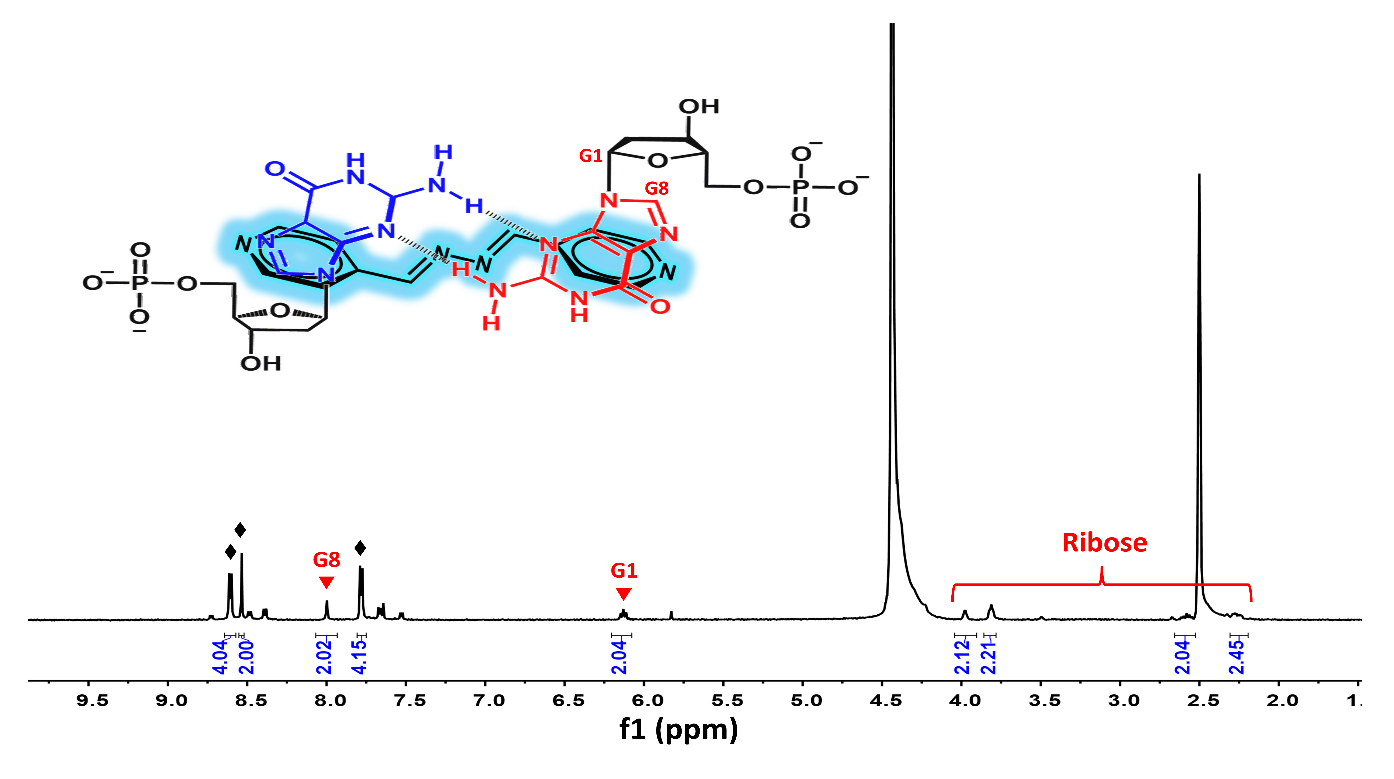


**Supplementary Figure 24** ^1^H–NMR spectroscopic observations for the formation of the G=G nucleotide base pair (g–motif). ^1^H NMR spectrum of **4** in solvent (D_2_O : DMSO–*d_6_* = 2:1 ) at room temperature (500 MHz, 300 K).

# XRPD Patterns


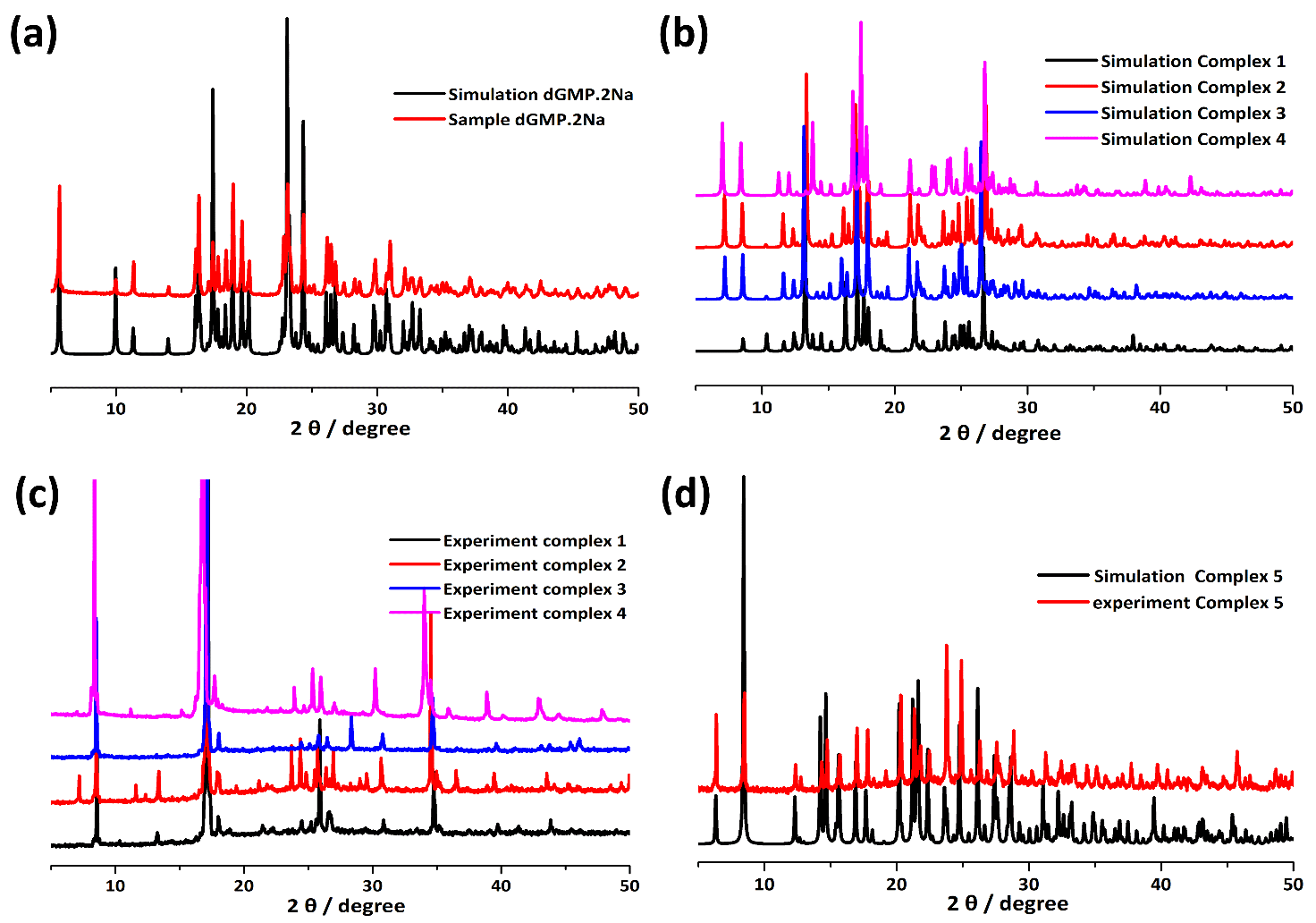


**Supplementary Figure 25** PXRD patterns show the comparison between the experimental values and calculated ones for **(a)** dGMP.2Na, **(b)** and **(c)** complexes **1–4**, and **(d)** complex **5**.

# IR Spectra

The infrared characteristic bands of the dGMP.2Na ligand include: *ν*(O–H) =3500–3000cm^–1^, *ν*(C=O)=1695cm^–1^, *ν*(C–N)=1490–1501cm^–1^, *δ*(N–H)=1653cm^–1^ in the purine base; *ν*_as_(P–O)=1077cm^–1^, *ν*_s_(P–O)=976cm^–1^ in the phosphoric group; *ν*(O–H)=3500–3200cm^–1^, *γ*(O–H)=781cm^–1^ and *ν*(C–O)=1261cm^–1^ in the Sugar ring. The characteristic bands of auxiliary ligands include *δ*(C–N)=1594 cm^–1^, *δ*(C–H)=1415 cm^–1^, *ν*(C–N)=980 cm^–1^, 990 cm^–1^, *ν*(C–C)=824 cm^–1^, *γ*(C–H)=545 cm^–1^. Comparing the infrared spectrum of complexes **1–5** with its ligands, we found that the infrared spectrum of the complexes includes not only the characteristic band of the dGMP.2Na ligand, but also the characteristic band of the corresponding auxiliary ligand. Therefore, we infer that the complexes **1–4** are all ternary complexes.

A strong band at 1695 cm^–1^ in the infrared spectrum of the dGMP.2Na ligand is assigned to the C(6)=O stretching vibration (Ghomi, and Taillandier, 1985). In the crystal structure of the dGMP.2Na ligand, the sodium ions are bonded to the carbonyl group with the Na–O distances of 2.55 to 2.35 Å. Since the C(6)=O stretching vibration of the dGMP.2Na salt at 1695 cm^–1^ showed no major shifting in the spectrum of the H_2_dGMP (at 1696 cm^–1^, the spectra are not shown), the Na–carbonyl interaction should be largely electrostatic (Emerson and Sundaralingam, 1980). Compared to the dGMP.2Na ligand, the carbonyl stretching vibration were observed at 1687cm^–1^ in complex **4, 5** and at 1664–1667 cm^–1^ in the complexes **1–3**. The major shifts of the carbonyl stretching vibration towards lower frequencies, which related to the indirect metal carbonyl interaction through an H_2_O molecule, and the C(6)=O group should be involved in the H–bonding network (Gellert et al., 1979).

At 1653cm^–1^ in the infrared spectrum of the dGMP.2Na ligand is assigned to the NH_2_ bending mode (Ghomi, and Taillandier, 1985). The shift of the NH_2_ bending mode in complexes, is due to the rearrangements of the NH_2_ hydrogen bonding network. It is worth noting that the bending vibration (1660–1630 cm^–1^) of the bonded H_2_O molecule is also appearing in this region. Since the dGMP.2Na and its complex all contain bonded water molecules, so it is difficult to determine the presence of the band at about 1653 cm^–1^ to the bending mode of the H_2_O or NH_2_ group.

The ring skeletal vibration of the dGMP.2Na ligand was observed as a sharp band at 1605 cm^–1^, which was assigned to the pyrimidine and imidazol ring vibrations (Ghomi, and Taillandier, 1985). This band was observed at 1593–1600 cm^–1^ in the complexes **1–3**, shifted towards a lower frequency. In contrast, the major upward shift of this band from 1605 to 1609 cm^–1^ and 1612 cm^–1^ in the spectrum of complex **4** and complex **5**, respectively. The different coordination modes of the N(7) group, can largely affect the electron distributions within the ring systems.

A sharp band at 1492 cm^–1^ of the dGMP.2Na, assigned to the imidazol C(8)–N(7) stretching and C(8)–H bending modes (Taboury et al., 1984). In the crystal structure of the dGMP.2Na, there is no direct interaction between the Na^+^ and the N(7) site, whereas a direct M–N(7) coordination was observed in the crystal structures of the Ni/Co–dGMP complexes. In the spectrum of the complex **5**, this band was split into two components at 1491 and 1501 cm^–1^, and this observed spectral changes is indicative of a direct M–N(7) binding in complex **5**. However, it is imrortant to note that the spectral features of the imidazol ring (1500–1480 cm^–1^) and the double–bond stretching region (1685–1612 cm^–1^) of the complex **4** spectrum showed major intensity changes and shifted towards a lower frequency, which different from complexes **1–3** and these are indicative of a different base binding mode in the complex **4**.

The two strong and broad bands at 1077 and 982cm^–1^, in the infrared spectrum of the dGMP.2Na, were assigned to the symmetric and antisymmetric PO_3_^2–^ and the bands at 1084 and 1095 cm^–1^ are coming from the sugar vibrational frequencies (Tajmir-Riahi, 1989). The PO_3_^2–^ antisymmetric and symmetric stretching vibrations of the complex **5** was also observed at 1077 and 982cm^–1^, which corresponds to the position of the dGMP.2Na ligand. However, the phosphate vibrations showed marked spectral changes with shifting towards lower frequencies, in the spectra of the complexes **1–4**, this may be caused by the rearrangement of the hydrogen bond network due to the protonation of the phosphate oxygen atoms.


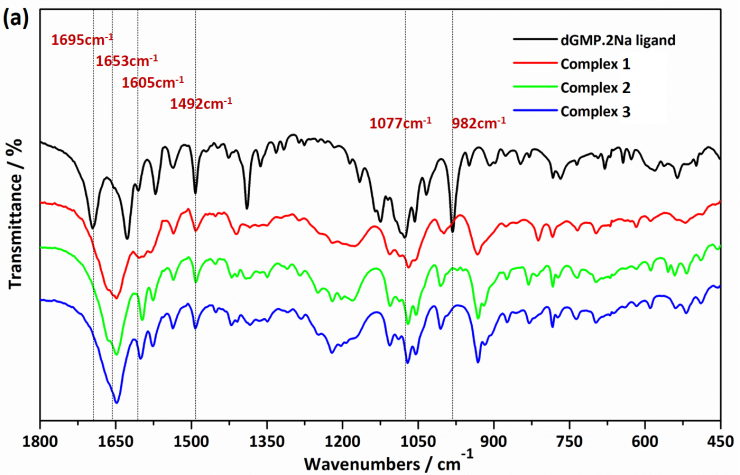

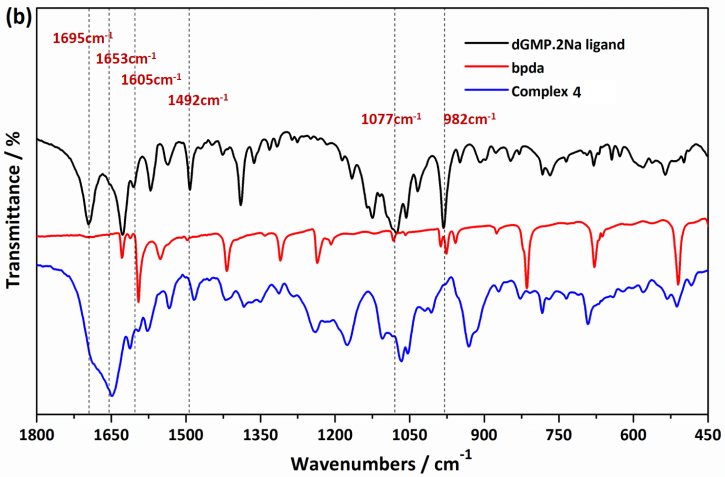


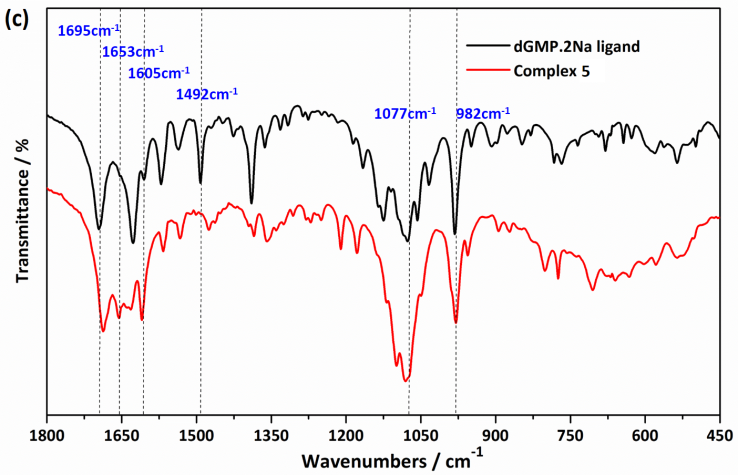

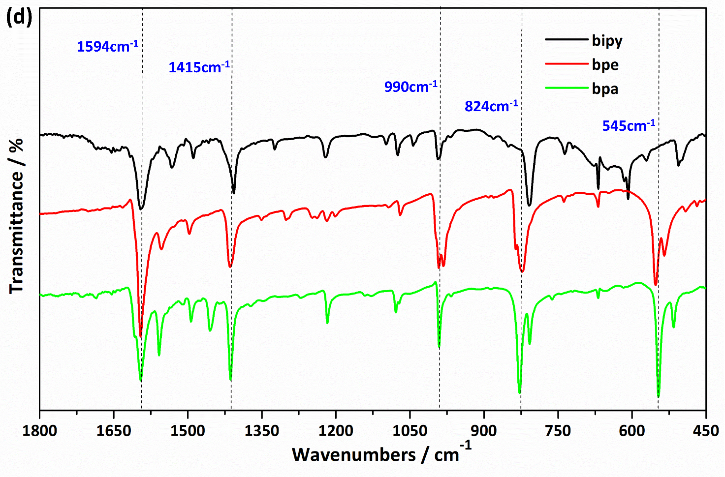


**Supplementary Figure 26** IR spectra of **(a)** dGMP.2Na ligand and complexes **1–3**; **(b)** dGMP.2Na ligand, bpda and complex **4**; **(c)** dGMP.2Na ligand and complex **5**; and **(d)** bipy, bpe and bpa.

# Thermo–Gravimetric Analysis


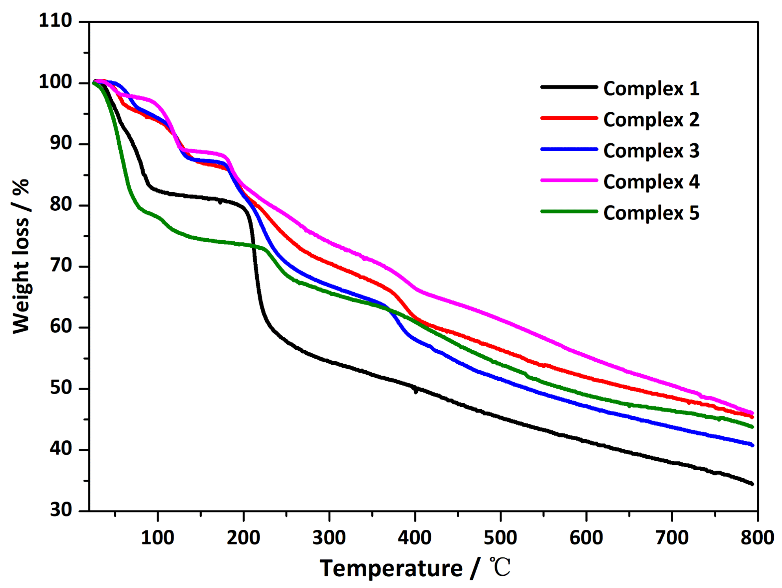


**Supplementary Figure 27** Thermo–Gravimetric Analysis (TGA) of **1** (black), **2** (red), **3** (blue), **4** (pink), and **5** (green) under dry N_2_ atmosphere. The weight loss stage of **1** is from 25°C to 120°C, and the mess loss (cal.18.2, calculated value 18.9%) is related to the loss of two guest water molecules and one 4,4’–bipyridine ligand. The collapse temperature is above 200°C. The first weight loss stage of **2** occurs at 70‒160°C, and the mass loss is 13.1%, corresponding to four coordinated water molecules and four free water molecules (calculated value 13.4%); The second stage of weight loss is from 200°C to 365°C, and the mess loss (cal.16.8%, calculated value 16.9%) is related to the loss of a free auxiliary ligand bpe, after which the complex skeleton began to collapse rapidly. The Thermo–Gravimetric Analysis curve of **3** is similar to that of **2**. Complex **4** loses 12.2% of its mass at 50‒140°C, corresponding to four coordinated water molecules and four free water molecules (calculated value 12.8%). When the temperature is higher than 190°C, the framework of Complex **4** begins to collapse. The mass loss of ca. 26.7 % from 25°C to 212°C is related to the loss of three guest water solvates and five coordinated water molecules (calculated value 26.9 %) for **5**. And it begins to collapse at above 212°C.

# References

1. Bisht, K. K., Suresh, E. (2013). Spontaneous resolution to absolute chiral induction: pseudo-Kagome type homochiral Zn(II)/Co(II) coordination polymers with achiral precursors. *J. Am. Chem. Soc.* 135, 15690-15693. doi: 10.1021/ja4075369
2. Dolomanov, O. V., Bourhis, L. J., Gildea, R. J., Howard, J. A. K., Puschmann, H. (2009). OLEX2: a complete structure solution, refinement and analysis program. *J. Appl. Crystallogr.* 42, 339-341. doi: 10.1107/S0021889808042726
3. Sheldrick, G. M. (2015). SHELXT - integrated space-group and crystal-structure determination. *Acta Crystallogr A Found Adv* 71, 3-8. doi: 10.1107/S2053273314026370
4. Kuo, L. Y., Kanatzidis, M. G., Sabat, M., Tipton, A. L., Marks, T. J. (1991). Metallocene antitumor agents. Solution and solid-state molybdenocene coordination chemistry of DNA constituents. *J. Am. Chem. Soc.* 113, 9027-9045. doi: 10.1021/ja00024a002
5. Dračínský, M., Šála, M., Hodgkinson, P. (2014). Dynamics of water molecules and sodium ions in solid hydrates of nucleotides. *CrystEngComm* 16, 6756-6764. doi: 10.1039/C4CE00727A
6. Young, D. W., Tollin, P., Wilson, H. R. (1974). The structure of disodium deoxyguanosine-5'-phosphate tetrahydrate. *Acta Crystallographica Section B Structural Crystallography and Crystal Chemistry* 30, 2012-2018. doi: 10.1107/S0567740874010892
7. Sponer, J., Leszczynski, J., Hobza, P. (2001). Electronic properties, hydrogen bonding, stacking, and cation binding of DNA and RNA bases. *Biopolymers* 61, 3-31. doi: 10.1002/1097-0282(2001)61:1<3::Aid-bip10048>3.0.Co;2-4; (b)Donald, V., Alexander, R. (1970). The Crystal Structures of Purines, Pyrimidines and Their Intermolecular Complexes. *Prog. Nucleic Acid Res. Mol. Biol.* 10. doi:
8. Qiu, Q. M., Gu, L., Ma, H., Yan, L., Liu, M., Li, H. (2018). Double layer zinc-UDP coordination polymers: structure and properties. *Dalton Trans.* 47, 14174-14178. doi: 10.1039/c8dt01537f
9. Ghomi, M., Taillandier, E. (1985). Normal coordinate analysis of 5'-dGMP and its deuterated derivatives. A calculated approach to designate the guanine-residue vibration modes in B and Z forms of DNA. *Eur. Biophys. J.* 12, 153-162. doi: 10.1007/BF00254073
10. Emerson, J., Sundaralingam, M. (1980). Zwitterionic character of guanosine 5'-monophosphate (5'-GMP): redetermination of the structure of 5'-GMP trihydrate. *Acta Crystallogr., Sect. B: Struct. Crystallogr. Cryst. Chem.* doi: 10.1016/S0079-6603(08)60565-6
11. Gellert, R. W., Shiba, J. K., Bau, R. (1979). X-Ray crystal and molecular structures of the Ni(II) and Co(II) complexes of 2′-deoxyguanosine-5′-monophosphate. *Biochem. Biophys. Res. Commun.* 88, 1449-1453. doi: 10.1016/0006-291X(79)91142-2
12. Taboury, J. A., Bourtayre, P., Liquier, J., Taillandier, E. (1984). Interaction of Z form poly(dG-dC).poly(dG-dC) with divalent metal ions: localization of the binding sites by I.R. spectroscopy. *Nucleic Acids Res.* 12, 4247-4258. doi: 10.1093/nar/12.10.4247
13. Tajmir-Riahi, H.-A. (1989). A comparative study of adenylic, guanylic and deoxyguanylic acids and their sodium salts as solid and in solution: structural information and conformational features. *Biochimica et Biophysica Acta (BBA) - Gene Structure and Expression* 1009, 168-176. doi: 10.1016/0167-4781(89)90097-3
